# Astrocyte glucocorticoid receptor signalling restricts neuronal plasticity

**DOI:** 10.1038/s41586-026-10512-9

**Published:** 2026-05-20

**Authors:** Bruno Gegenhuber, Takuma Sonoda, Lisa Traunmüller, Christopher P. Davis, Shon A. Koren, Eric C. Griffith, Chinfei Chen, Michael E. Greenberg

**Affiliations:** 1 Department of Neurobiology, Harvard Medical School, Boston, MA USA; 2 Department of Neurology, F. M. Kirby Neurobiology Center, Boston Children’s Hospital, Boston, MA USA

**Keywords:** Molecular neuroscience, Astrocyte, Glial development, Epigenetics and plasticity, Visual system

## Abstract

Sensory experience refines neural circuits during critical periods of postnatal development^1–3^. Although neuronal activity is known to orchestrate the circuit wiring that underlies this process^4,5^, the environmental cues that restrain developmental plasticity as animals mature are less clear. Here we examine the experience-dependent maturation of the mouse primary visual cortex across postnatal development using paired single-cell transcriptomic and chromatin accessibility sequencing. In addition to identifying the activity-dependent gene programs that emerge within each cortical cell type, we find that light exposure drives astrocyte maturation through cell-type-specific recruitment of the glucocorticoid receptor (encoded by *Nr3c1*) to chromatin. Astrocyte glucocorticoid receptor signalling activates an extensive gene regulatory program that is partially conserved in human brain development and promotes maturation processes that may regulate critical period closure. Collectively, these findings reveal that astrocyte glucocorticoid receptor signalling restricts neuronal plasticity. Glucocorticoid regulation of astrocyte maturation may also contribute to the effects of early-life stress across the brain, and the disruption of this process may increase susceptibility to neuropsychiatric disease.

## Abstract (web summary)

Combined single-cell transcriptomic and chromatin accessibility sequencing analysis of mouse primary visual cortex across postnatal development reveals that the glucocorticoid receptor drives astrocyte maturation to limit neuronal plasticity.

## Main

Sensory experience shapes the development of neural circuits during early-life windows of heightened plasticity, known as critical periods. This process has been extensively studied in mammalian primary visual cortex (V1), where temporarily blocking vision in one eye in early life results in persistent loss of cortical responsiveness to the deprived eye^1^. Fast-spiking parvalbumin (PV)-expressing interneurons, which densely innervate the soma of excitatory pyramidal neurons, are known to gate the onset of critical periods by enabling competitive plasticity within cortical networks^4–6^, and several secreted^7,8^ and cell-intrinsic molecules^9,10^ that facilitate PV interneuron maturation have been identified. By contrast, the specific factors that restrain early-life plasticity remain poorly understood, although recent studies indicate an essential role for astrocytes^11,12^ and oligodendrocytes^13,14^ in critical period closure.

Aberrant critical period timing has been implicated in the aetiology of neurodevelopmental disorders, such as autism spectrum disorder^15^ and schizophrenia^16^, and thus the possibility of modifying adult plasticity holds tremendous potential for restoring brain function in these disorders. Here, by characterizing how experience regulates gene expression at cell-type resolution across V1 postnatal development, we identified a gene regulatory mechanism that promotes visual system maturation and may contribute to critical period closure.

## Atlas of experience-dependent V1 development

**Fig. 1:**
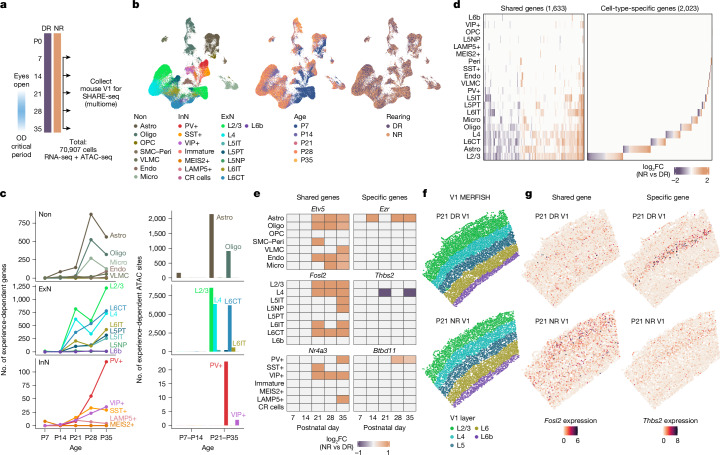
Molecular atlas of experience-dependent V1 development. **a**, Timeline for developmental mouse V1 SHARE-seq experiment. OD, ocular dominance. **b**, Uniform manifold approximation and projection (UMAP) visualization of V1 multiome snRNA-seq data, coloured by cell identity (left), age in postnatal days (middle) and rearing condition (right). Astro, astrocyte; CR cells, Cajal–Retzius cells; Endo, endothelial cell; ExN, excitatory neuron; InN, inhibitory neuron; L5IT, L5 intratelencephalic; L5NP, L5 near-projecting; L5PT, L5 pyramidal tract; L6CT, L6 corticothalamic; Micro, microglia; Oligo, oligodendrocyte; Non, non-neuronal cell types; OPC, oligodendrocyte progenitor cell; Peri, pericyte; SMC, smooth muscle cell; VLMC, vascular leptomeningeal cell. **c**, Left, number of experience-dependent genes per cell type and age, grouped by non-neuronal cell types (top), excitatory neuron cell types (middle) and inhibitory neuron cell types (bottom) (pseudobulk edgeR QLFTest, two-sided, adjusted *P* value (*P*_adj_) < 0.05). Right, number of experience-dependent chromatin regions per cell type and age window, grouped by non-neuronal cell types (top), excitatory neuron cell types (middle), and inhibitory neuron cell types (bottom) (pseudobulk edgeR QLFTest, two-sided, *P*_adj_ < 0.1). **d**, Heat map of log_2_(fold change (FC)) values of all shared (at least 2 cell types) and cell-type-specific experience-dependent genes detected in any cell type and age during postnatal development (pseudobulk edgeR QLFTest, two-sided, *P*_adj_ < 0.05). Positive fold change, NR bias; negative fold change, DR bias. **e**, Heat map of example shared and cell-type-specific experience-dependent genes. **f**, P21 MERFISH data, coloured by V1 layer identity. **g**, Number of transcripts per cell of *Fosl2* (left) and *Thbs2* (right) in V1 from P21 DR and NR mice.

**Extended Data Fig. 1:**
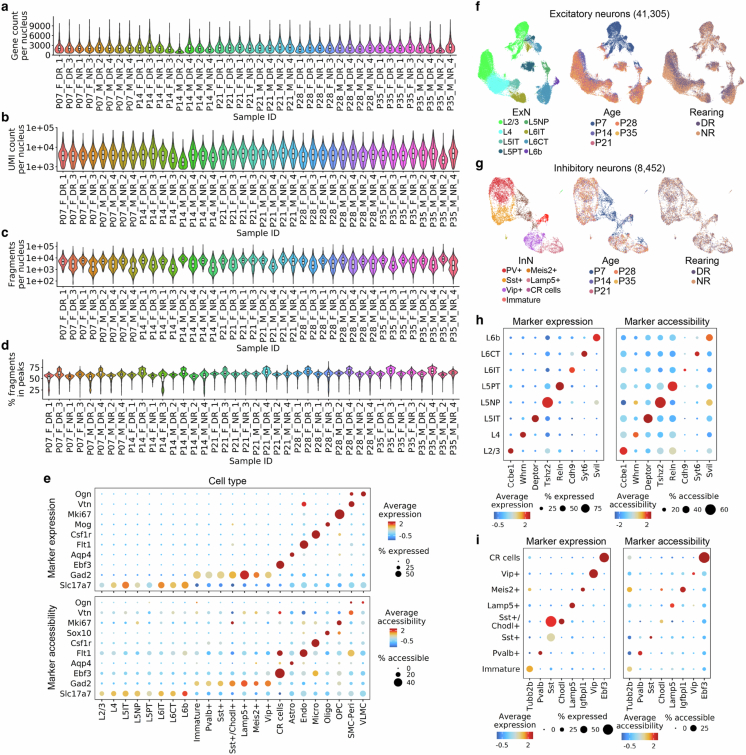
Additional analysis of postnatal V1 single-nucleus multiome dataset. **a-d**, RNA (**a**-**b**) and ATAC (**c**-**d**) quality control metrics (**a**, gene count per nucleus, **b**, UMIs per nucleus, **c**, ATAC fragments per nucleus, **d**, percentage of fragments in peaks) for V1 SHARE-seq multiome experiment, colored by sample ID. Nuclei number: P07_F_DR_1 *n* = 525, P07_F_DR_3 *n* = 1,619, P07_F_NR_1 *n* = 1,221, P07_F_NR_3 *n* = 4,120, P07_M_DR_2 *n* = 1,009, P07_M_DR_4 *n* = 2,308, P07_M_NR_2 *n* = 483, P07_M_NR_4 *n* = 3,007, P14_F_DR_1 *n* = 846, P14_F_DR_3 *n* = 2,241, P14_F_NR_1 *n* = 397, P14_F_NR_3 *n* = 1,659, P14_M_DR_2 *n* = 982, P14_M_DR_4 *n* = 3,123, P14_M_NR_2 *n* = 617, P14_M_NR_4 *n* = 2,008, P21_F_DR_1 *n* = 735, P21_F_DR_3 *n* = 2,599, P21_F_NR_1 *n* = 1,221, P21_F_NR_3 *n* = 3,016, P21_M_DR_2 *n* = 1,185, P21_M_DR_4 *n* = 2,687, P21_M_NR_2 *n* = 1,150, P21_M_NR_4 *n* = 3,299, P28_F_DR_1 *n* = 988, P28_F_DR_3 *n* = 1,998, P28_F_NR_1 *n* = 858, P28_F_NR_3 *n* = 2,433, P28_M_DR_2 *n* = 966, P28_M_DR_4 *n* = 2,175, P28_M_NR_2 *n* = 941, P28_M_NR_4 *n* = 2,763, P35_F_DR_1 *n* = 1,276, P35_F_DR_3 *n* = 1,975, P35_F_NR_1 *n* = 1,285, P35_F_NR_3 *n* = 2,703, P35_M_DR_2 *n* = 764, P35_M_DR_4 *n* = 2,539, P35_M_NR_2 *n* = 1,206, P35_M_NR_4 *n* = 3,980. Boxplot centre, median; box boundaries, first and third quartiles; whiskers, 1.5 × IQR from boundaries. **e**, Dotplot of marker gene expression (top) and gene body accessibility (bottom) across V1 cell types. **f-g**, UMAPs of multiome snRNA V1 excitatory neuron (**f**) and inhibitory neuron (**g**) subtypes, colored by cell identity (left), age in postnatal days (middle), and rearing condition (right). **h-i**, Dotplot of marker gene expression (left) and gene body accessibility (right) across V1 excitatory neuron (**h**) and inhibitory neuron (**i**) subtypes.

We performed simultaneous high-throughput assay for transposase-accessible chromatin (ATAC) and RNA expression with sequencing (SHARE-seq)^17^ of V1 of normal-reared (NR) and dark-reared (DR) mice at five ages, capturing key developmental milestones, including pre-eye opening (postnatal day 7 (P7)), eye opening (P14), ocular dominance critical period (P21–P28) and critical period closure (P35) (Fig. 1a and Extended Data Fig. 1a–d). De novo clustering of 70,907 cells revealed principal cortical cell classes and subtypes with matched correspondence of marker gene expression and gene body accessibility (Fig. 1b and Extended Data Fig. 1e–i). To identify responses to visual experience, we performed differential expression and accessibility analysis between NR and DR within each cell type and at each time point. Visual experience generated widespread changes in gene expression and chromatin state across cortical excitatory neuron types, particularly within layer 2/3 (L2/3) excitatory neurons, which require vision for cell fate specification^18^, as well as non-neuronal cell types, including astrocytes (Fig. 1c and Supplementary Tables 1–3). Further analysis revealed that the timing of these experience-dependent effects aligned to key stages in V1 development, with excitatory and inhibitory neurons responding most to visual deprivation at P21 and P35, ages that correspond to critical period onset and closure, respectively (Fig. 1c and Supplementary Table 2). By contrast, the glial response to visual deprivation peaked at P28 (Fig. 1c and Supplementary Table 2) and was driven largely by experience-dependent regulation of genes involved in cell-type-specific maturational processes that are important for circuit refinement, including myelination (*Myrf* and *Mobp*) in oligodendrocytes^13,14^ and surveillance (*Csf1r* and *Tgfb1*) in microglia^19^.

**Extended Data Fig. 2:**
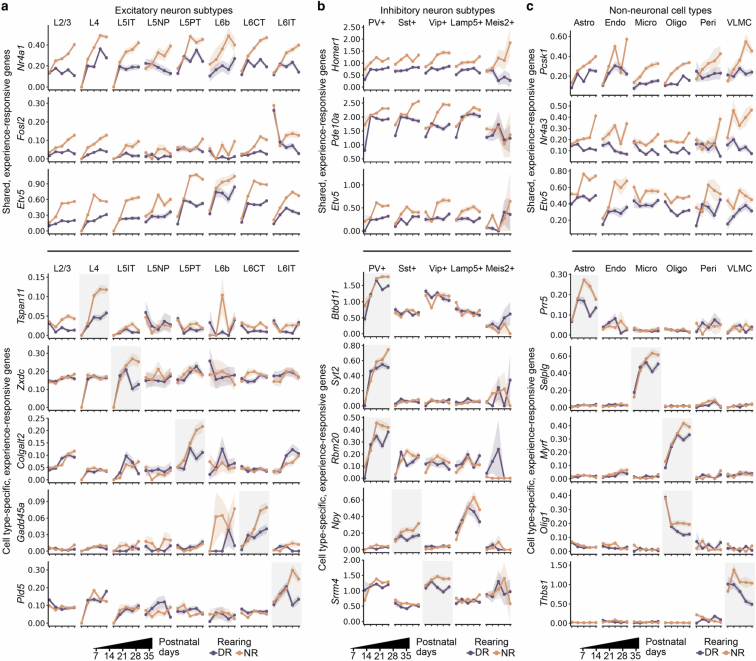
Cell type-specific experience-responsive genes across V1 development. **a**, Mean normalized expression ± s.e.m. of example shared (*Nr4a1*, *Fosl2*, and *Etv5*) and cell type-specific (*Tspan11*, *Zxdc*, *Colgalt2*, *Gadd45a*, and *Pld5*) experience-responsive genes in excitatory neuron subtypes across postnatal development. **b**, Mean normalized expression ± s.e.m. of example shared (*Homer1*, *Pde10a*, and *Etv5*) and cell type-specific (*Btbd11*, *Syt2*, *Rbm20*, *Npy*, and *Srrm4*) experience-responsive genes in inhibitory neuron subtypes across postnatal development. **c**, Mean normalized expression ± s.e.m. of example shared (*Pcsk1*, *Nr4a3*, and *Etv5*) and cell type-specific (*Prr5*, *Selplg*, *Myrf*, *Olig1*, and *Thbs1*) experience-responsive genes in non-neuronal cell types across postnatal development.

**Extended Data Fig. 3:**
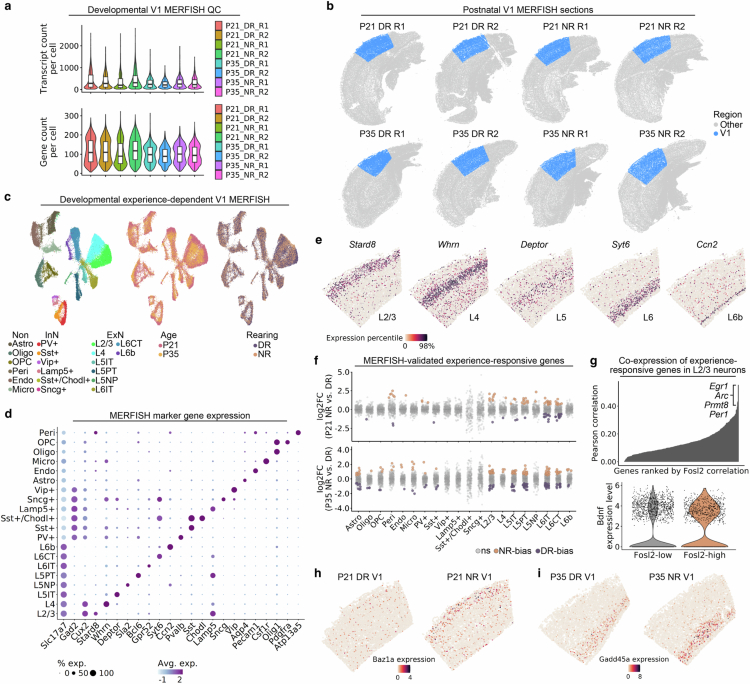
Additional analysis of V1 MERFISH dataset. **a**, MERFISH QC metrics (top: transcripts per nucleus, bottom: genes per nucleus). Cell number: P21_DR_R1 *n* = 7,836, P21_DR_R2 *n* = 5,398, P21_NR_R1 *n* = 8,280, P21_NR_R2 *n* = 5,657, P35_DR_R1 *n* = 6,087, P35_DR_R2 *n* = 5,523, P35_NR_R1 *n* = 5,170, P35_NR_R2 *n* = 5,152. Boxplot centre, median; box boundaries, first and third quartiles; whiskers, 1.5 × IQR from boundaries. **b**, Aligned MERFISH sections colored by V1 ROI. **c**, UMAPs of developmental V1 MERFISH dataset, colored by cell identity (left), age in postnatal days (middle), and rearing condition (right). **d**, Dotplot of marker gene expression across V1 cell types. **e**, Normalized expression of V1 excitatory neuron layer markers (L2/3: *Stard8*, L4: *Whrn*, L5: *Deptor*, L6: *Syt6*, L6b: *Ccn2*) in P35 NR R1 MERFISH sample. **f**, Number and log2(Fold change) of experience-dependent genes in each cell type at P21 (top) and P35 (bottom) (pseudobulk edgeR LRT test, two-sided, *P*_adj_ < 0.1). **g**, (top) Pearson correlation between expression of *Fosl2* and all other genes in NR L2/3 neurons across P21 and P35. Top genes correlated with *Fosl2* are shown. (Bottom) *Bdnf* expression in *Fosl2*- and *Fosl2*+ NR L2/3 neurons. **h-i**, Additional examples of MERFISH-validated cell type-specific, experience-responsive genes: *Baz1a* in L2/3 neurons (**h**) and *Gadd45a* in L6CT neurons (**i**).

**Extended Data Fig. 4:**
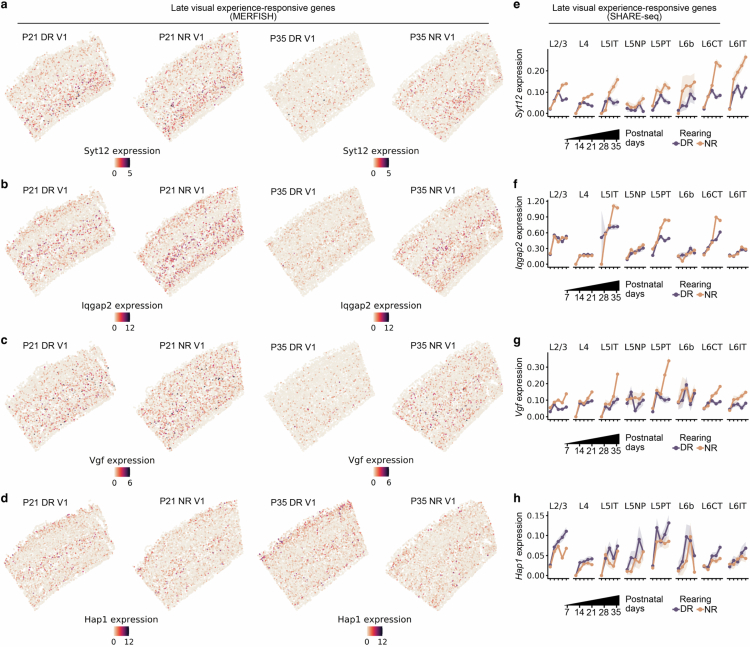
Representative late visual experience-responsive genes across MERFISH and SHARE-seq datasets. **a-d**, Example visual experience-responsive genes in excitatory neurons at P35 and not P21 (pseudobulk edgeR LRT test, two-sided, *P*_adj_ < 0.1) detected by MERFISH: *Syt12* (**a**), *Iqgap2* (**b**), *Vgf* (**c**), and *Hap1* (**d**). **e-h**, Example mean expression ± s.e.m. of visual experience-responsive genes in excitatory neurons at P35 and not P21 (pseudobulk edgeR QLFTest, two-sided, *P*_adj_ < 0.05) detected by SHARE-seq: *Syt12* (**e**), *Iqgap2* (**f**), *Vgf* (**g**), and *Hap1* (**h**).

To determine the extent to which light exposure-dependent gene expression differs across V1 cell types, we performed hierarchical clustering of differentially expressed genes across all time points and found that approximately 40% of gene changes occur within multiple cell types and include classic immediate early genes, such as *Fosl2*, *Egr1* and *Nr4a1*, whereas the remaining visual experience-dependent changes in gene expression occur within individual cell types (Fig. 1d,e and Extended Data Fig. 2a–c). These cell-type-specific, experience-dependent genes predominantly encode cell surface and secreted proteins that may coordinate cell-type-specific wiring of V1 circuitry, such as *Thbs2* in L4 neurons and *Btbd11* in PV interneurons (Fig. 1e and Extended Data Fig. 2a–c). To validate our results from the SHARE-seq dataset, we performed multiplexed error-robust fluorescence in situ hybridization (MERFISH) on V1 sections from NR and DR mice at P21 and P35, using a 500-gene panel enriched for the cell-type-specific, experience-responsive genes identified by SHARE-seq (Fig. 1f,g, Extended Data Fig. 3a–i and Supplementary Table 4). Comparison of the SHARE-seq and MERFISH modalities validated several experience-responsive genes, and using MERFISH, we found that many experience-responsive genes span the medial–lateral axis of V1, including monocular and binocular regions (Extended Data Figs. 3f–i and 4a–h). Further analyses across postnatal ages revealed several genes regulated by experience at P35 and not at P21 in excitatory neurons, including those coordinating synaptic wiring (*Cdh13*, *Nrxn3*, *Syt12* and *Vgf*) and stabilization (*Hap1*, *Iqgap2*, *Mpp3* and *Tspan13*) (Extended Data Fig. 4a–h).

**Fig. 2:**
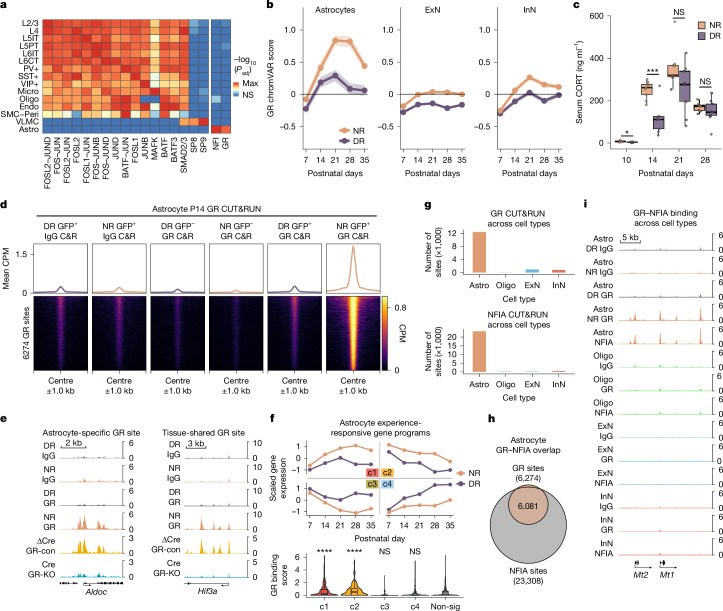
Experience-dependent genomic targets of GR in astrocytes. **a**, Heat map of −log10(*P*_adj_) values (row-scaled) for top experience-induced chromVAR transcription factor motif scores (using MAST, *P*_adj_ < 0.05). Max, maximum. **b**, GR chromVAR score (mean ± s.e.m.) in NR and DR astrocytes (left), excitatory neurons (middle) and inhibitory neurons (right). **c**, ELISA for CORT in serum from NR and DR mice. Two-sided *t*-test with Benjamini–Hochberg correction; *n* = 9 per condition. P10: *P*_adj_ = 0.04; P14: *P*_adj_ = 6.2 × 10^−4^. **d**, Line plots (top) and heat maps (bottom) of mean IgG and GR CUT&RUN (C&R) counts per million (CPM) ±1 kb around experience-dependent GR sites in GFP^−^ and GFP^+^ nuclei (DiffBind edgeR QLFTest, two-sided, *P*_adj_ < 0.01). *n* = 1 for GFP^+^ IgG, *n* = 2 for GFP^−^ GR, *n* = 3 for GFP^+^ GR. Colour scale indicates CPM. **e**, IgG and GR CUT&RUN signal at *Aldoc* (left) and *Hif3a* (right). GR-con, Astro-ΔCre AAV in *GR*^*fl/fl*^; GR-KO, Astro-Cre AAV in *GR*^*fl/fl*^. **f**, Top, scaled expression (mean ± s.e.m.) of astrocyte experience-dependent genes clustered using hierarchical clustering (edgeR QLFTest, two-sided, *P*_adj_ < 0.05). c1, 630 genes; c2, 236 genes; c3, 219 genes; c4, 170 genes. Bottom, GR binding enrichment score for c1, c2, c3 and c4 gene clusters and non-differentially expressed genes (non-sig). One-sided *t*-test comparison of scores for each cluster to scores for non-differential genes. c1: *P* = 1.3 × 10^−15^; c2: *P* = 3.1 × 10^−11^; c3: *P* = 1; c4: *P* = 0.98. **g**, P14 GR (top) or NFIA (bottom) sites in astrocytes, oligodendrocytes, excitatory neurons and inhibitory neurons (MACS2, *q* < 0.01). **h**, Overlap of astrocyte GR and NFIA sites. **i**, Astrocyte-specific GR and NFIA co-binding at the *Mt1*/*Mt2* locus. In all box plots, the centre line is the median, box boundaries delineate first and third quartiles and whiskers extends to 1.5× interquartile range from the box boundaries. *P < 0.05, **P < 0.01, ***P < 0.001, ****P < 0.0001; NS, not significant (*P* ≥ 0.05). Source data

**Extended Data Fig. 5:**
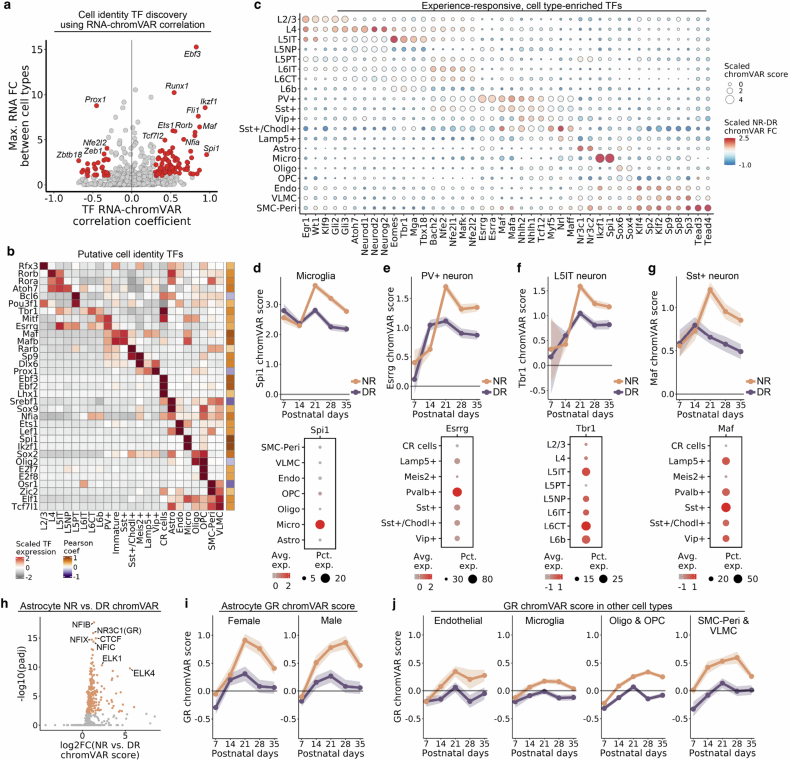
Identification of cell type-specific, experience-responsive TF motifs across V1 development. **a**, Pearson correlation coefficient (PCC) between TF expression and chromVAR motif score across V1 cells plotted against the maximum pairwise FC of TF expression between cell types. Putative cell identity TFs (PCC > 0.3 & maximum RNA FC > 1) indicated in red. Negative PCC values indicate predicted repressors, and positive PCC values indicate predicted activators. **b**, Heat map of scaled RNA expression of top putative cell identity TFs across V1 cell types. PCC between TF expression and chromVAR motif score shown on the right. **c**, Top experience-responsive, cell type-enriched TFs across V1 cell types, sized by chromVAR score (scaled across cell types) and colored by NR-DR chromVAR FC (scaled across cell types). **d-g**, Example experience-responsive, cell type-enriched TFs in microglia (*Spi1*/PU.1) (**d**), PV interneurons (*Esrrg*) (**e**), deep-layer excitatory neurons (*Tbr1*) (**f**), and SST interneurons (*Maf*) (**g**). For each TF and cell type, mean chromVAR score ± s.e.m. in NR and DR cells across postnatal development is shown (top), beside mean and per cent TF expression across cell types within each cell class (bottom). **h**, Volcano plot of experience-dependent chromVAR TF motif scores (MAST zlm likelihood-ratio test, two-sided, *P*_adj_ < 0.05) (see also Fig. 2a) in V1 astrocytes at P21. **i**, Mean GR chromVAR score ± s.e.m. (see also Fig. 2b) across postnatal development in NR and DR astrocytes from female (left), and male (right) mice. **j**, Mean GR chromVAR score ± s.e.m. across postnatal development in NR and DR endothelial cells (left), microglia (middle left), oligodendrocytes and oligodendrocyte precursor cells (middle right), and SMC-pericytes and VLMC cells (right).

To characterize the transcription factors that mediate experience-dependent gene expression across V1 development, we compared the genome-wide chromatin accessibility of all transcription factor motifs between NR and DR mice using chromVAR^20^ and found the consensus AP-1 (FOS–JUN) binding motif as the top experience-induced motif across nearly every cell type (Fig. 2a). In addition, we identified transcription factor motifs that increase accessibility with experience and are enriched within specific cell types (Extended Data Fig. 5a–g). This analysis revealed transcription factors that may mediate cell-type-specific, experience-responsive gene programs^21^, such as SPI1 (also known as PU.1) in microglia (Extended Data Fig. 5d), ESRRG in PV interneurons^10^ (Extended Data Fig. 5e), TBR1 in deep-layer excitatory neurons (Extended Data Fig. 5f) and MAF in somatostatin-expressing (SST) interneurons^22^ (Extended Data Fig. 5g). Collectively, these results demonstrate that early-life visual experience drives widespread, neuronal subtype-specific gene regulatory programs that are likely to coordinate V1 development.

## Light-dependent GR genomic recruitment in astrocytes

In contrast to all other cell types, the binding motifs for the glucocorticoid receptor (GR) and mineralocorticoid receptor, as well as nuclear factor I (NFI), emerged as the top visual experience-induced regulatory sites in astrocytes (Fig. 2a,b and Extended Data Fig. 5c,h–j). Consistent with the possibility that changes in chromatin state and gene expression in astrocytes are regulated by a mechanism that is distinct from that operating in neurons, we found that these responses to light exposure emerged earlier during postnatal development (Fig. 1c and Supplementary Tables 2 and 3).

In contrast to intermediate early gene transcription factors, which undergo rapid transcriptional induction in response to diverse stimuli^23^, GR is constitutively expressed but is retained in an inactive state in the cytoplasm until glucocorticoid binding drives its translocation to the nucleus^24^. We hypothesized that astrocytes, which ensheath parenchymal blood vessels^25^, might be uniquely poised to take up peripheral hormones such as glucocorticoids in response to light exposure. Previous studies have demonstrated that light increases circulating CORT in adult mice^26^ and cortisol in humans^27^, and that in mice, light-evoked CORT release requires activation of retinal ganglion neuron projections to the suprachiasmatic nucleus^26^. However, it was not known whether exposure to natural light around the time of eye opening (around P14) influences CORT levels.

**Extended Data Fig. 6:**
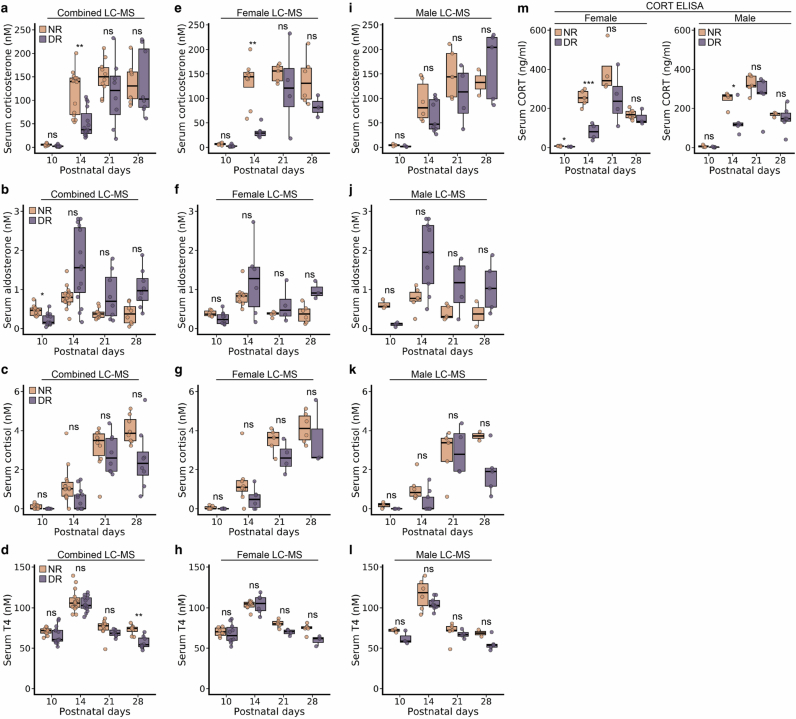
Additional hormone quantification across postnatal development using LC-MS. **a**-**l**, Serum concentration (nM) of corticosterone (**a**, **e**, **i**), aldosterone (**b**, **f**, **j**), cortisol (**c**, **g**, **k**), and thyroxine (T4) (**d**, **h**, **l**) detected by LC-MS in NR and DR mice at P10, P14, P21, and P28 across sexes (**a-d**), in females (**e-h**), and in males (**i-l**). Two-tailed *t*-test, adjusted for multiple hypothesis testing using Bonferroni method; Number of animals: NR P10 *n* = 9 (6 females/3 males), DR P10 *n* = 13 (10 females/3 males), NR P14 *n* = 14 (8 females/6 males), DR P14 *n* = 15 (6 females/9 males), NR P21 *n* = 10 (5 females/5 males), DR P21 *n* = 8 (4 females/4 males), NR P28 *n* = 8 (6 females/2 males), DR P28 *n* = 8 (3 females/5 males); ns, not significant; *P_adj_ < 0.05; **P_adj_ < 0.01. P14 combined sex corticosterone: *P*_adj_ = 4.0 × 10^−3^; P10 combined sex aldosterone: *P*_adj_ = 0.02; P28 combined sex T4: *P*_adj_ = 6.9 × 10^−3^; P14 female corticosterone: *P*_adj_ = 4.7 × 10^−3^. Boxplot centre, median; box boundaries, first and third quartiles; whiskers, 1.5 × IQR from boundaries. **m**, CORT ELISA on serum from NR and DR female (left) or male (right) mice at P10, P14, P21, and P28. Two-tailed *t*-test; NR P10 *n* = 5 females/4 males, DR P10 *n* = 4 females/5 males, NR P14 *n* = 5 females/4 males, DR P14 *n* = 4 females/5 males, NR P21 *n* = 4 females/5 males, DR P21 *n* = 4 females/5 males, NR P28 *n* = 6 females/3 males, DR P28 *n* = 3 females/6 males; ns, not significant; *P < 0.05; ***P < 0.001. P10 female: *P* = 0.023; P14 female: *P* = 6.5 × 10^−4^; P14 male: *P* = 0.036. Boxplot centre, median; box boundaries, first and third quartiles; whiskers, 1.5 × IQR from boundaries.

We measured serum CORT levels in DR and NR mice on postnatal days 10, 14, 21 and 28 by enzyme-linked immunosorbent assay (ELISA) and found that CORT levels increase at the time of eye opening (P14) in NR mice, whereas DR attenuates this increase (Fig. 2c). By contrast, DR has minimal effects on the levels of other circulating glucocorticoids or thyroxine (T4) as measured by liquid chromatography–mass spectrometry (LC–MS) (Extended Data Fig. 6a–d and Supplementary Table 5). The influence of light on the levels of these hormones did not differ qualitatively between female and male mice (Extended Data Fig. 6e–m). Together, these results indicate that light exposure around eye opening enhances systemic CORT production, which could lead to GR activation in astrocytes.

**Extended Data Fig. 7:**
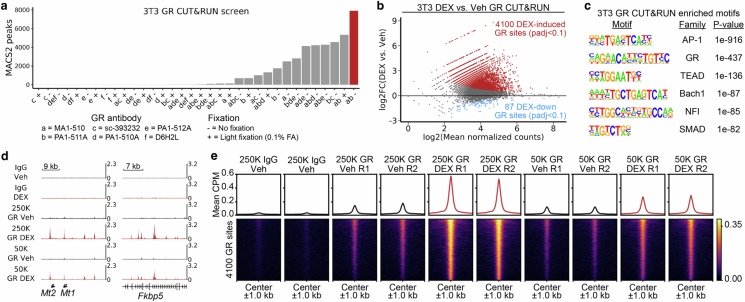
Validation of GR CUT&RUN in 3T3 cells. To optimize GR CUT&RUN, we tested a panel of commercial GR antibodies (single vs. cocktail) with or without light fixation (0.1% formaldehyde, 2 min), comprising a total of 34 conditions, on 250,000 hormone-starved 3T3 cells stimulated for 2 hr with 200 nM dexamethasone (DEX). **a**, Number of GR binding sites (MACS2, *q* < 0.01) detected in 3T3 cells for each GR CUT&RUN condition. Red indicates the condition used for all subsequent in vitro and in vivo GR CUT&RUN experiments. **b**, MA plot of DEX-dependent GR binding sites (DiffBind edgeR QLFTest, two-sided, *P*_adj_ < 0.1) (*n* = 2 replicates per condition). **c**, Top unique enriched motifs (HOMER hypergeometric test, one-sided, *P* < 0.05) in DEX-induced GR binding sites. **d**, Example DEX-induced GR binding to canonical GR targets *Mt1*/*Mt2* (left) and *Fkbp5* (right) in 250,000 3T3 cells and 50,000 3T3 cells. **e**, Line plots (top) and heat maps (bottom) of mean IgG and GR CUT&RUN CPM ± 1 kb around DEX-induced GR peaks in 250,000 or 50,000 3T3 cells. *n* = 1 per condition for IgG samples; *n* = 2 per condition for GR samples. Color scale indicates CPM.

**Extended Data Fig. 8:**
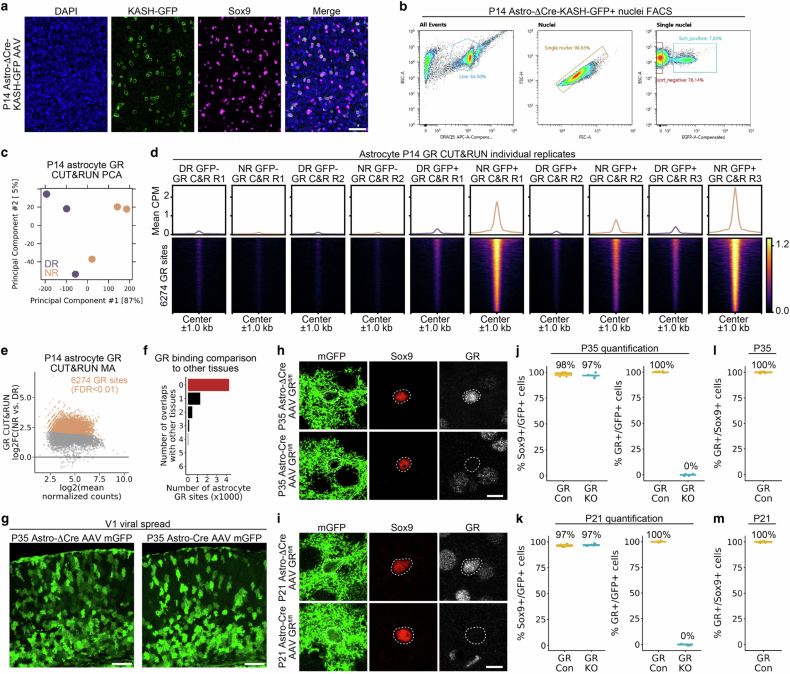
Additional analysis of astrocyte GR CUT&RUN and validation of GR-KO. **a**, Representative immunofluorescent images of DAPI (left), KASH-GFP (middle left), Sox9 (middle right), and merged channels (right) in Astro-ΔCre-T2A-KASH-GFP-infected V1 at P14. Scale, 50 µm. Experiment was performed once. **b**, Fluorescence-activated nuclei sorting (FANS) gating strategy to isolate GFP+ singlet nuclei from P14 V1 astrocytes with Astro-ΔCre-T2A-KASH-GFP AAV. left, DRAQ5+ nuclei gate; middle, singlet gate; right, GFP+ nuclei gate. **c**, Principal component analysis (PCA) of astrocyte GR CUT&RUN samples (*n* = 3 per condition). **d**, Line plots (top) and heat maps (bottom) of mean GR CUT&RUN CPM ± 1 kb around 6,274 experience-dependent GR sites for individual biological replicates (see also Fig. 2d). Color scale indicates CPM. **e**, MA plot of P14 astrocyte experience-dependent GR binding sites (DiffBind edgeR QLFTest, two-sided, *P*_adj_ < 0.01). **f**, Comparison of astrocyte GR sites to GR sites detected in mouse kidney^83^, liver^84^, primary brown preadipocytes^85^, embryonic fibroblasts^86^, mammary gland^87^, and primary bone marrow-derived macrophages^88^, accessed from the ReMAP2022 database^97^. **g**, Representative immunofluorescent images of V1 astrocyte membrane-GFP (mGFP) in Astro-ΔCre AAV *GR*^*fl/fl*^ (left) and Astro-Cre AAV *GR*^*fl/fl*^ (right) animals at P35. Scale, 200 µm. **h-i**, Representative immunofluorescent images of V1 astrocyte mGFP (left), Sox9 (middle), and GR (right) in Astro-ΔCre AAV *GR*^*fl/fl*^ (GR-Con) (top row) and Astro-Cre AAV *GR*^*fl/fl*^ (GR-KO) (bottom row) animals at P35 (**h**) and P21 (**i**). Dotted line indicates astrocyte nucleus. Scale, 10 µm. **j-k**, Percentage of V1 GFP+ cells labeled with Sox9 (left) or GR (right) in GR-Con and GR-KO animals at P35 (**j**) (GR-Con *n* = 6, GR-KO *n* = 6) and P21 (**k**) (GR-Con *n* = 6, GR-KO *n* = 5). **l-m**, Percentage of V1 Sox9+ cells labeled with GR in GR-Con animals at P35 (**l**) (*n* = 6) and P21 (**m**) (*n* = 6).

**Extended Data Fig. 9:**
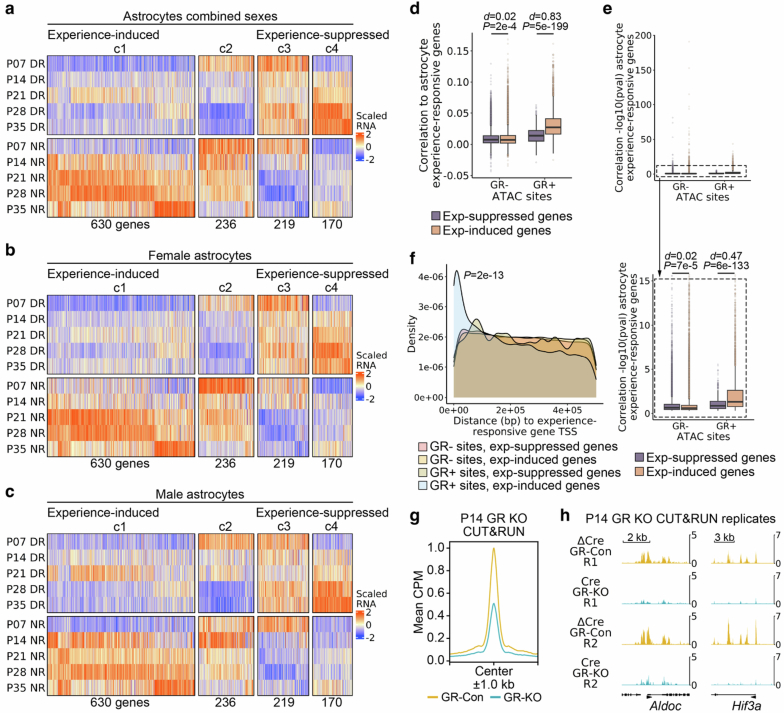
Additional analysis of experience-responsive genes and GR binding in astrocytes. **a-c**, Mean, scaled expression of astrocyte experience-dependent genes (edgeR QLFTest, two-sided, *P*_adj_ < 0.05) detected at any time point and clustered into 4 groups using hierarchical clustering (see also Fig. 2f) across sexes (**a**) and in females (**b**) and males (**c**) separately. **d-e**, Pearson correlation coefficient (**d**) or -log10(*P*-value) (**e**) between astrocyte experience-dependent genes (separated into experience-induced or experience-suppressed) and nearby ATAC sites (±500 Kb) that either exhibit (GR +) or lack (GR-) GR binding. *P*-values from Two-tailed *t*-test; Cohen’s *d* value estimate of effect size. Dotted box in (**e**) indicates inset panel. GR- ATAC sites near experience-suppressed genes *n* = 45,265 sites, GR- ATAC sites near experience-induced genes *n* = 112,776 sites, GR + ATAC sites near experience-suppressed genes *n* = 1,072 sites, GR + ATAC sites near experience-induced genes *n* = 4,369 sites. Boxplot centre, median; box boundaries, first and third quartiles; whiskers, 1.5 × IQR from boundaries. **f**, Distance between astrocyte experience-dependent gene transcription start sites (TSS) and nearby GR+ or GR- ATAC sites. GR + ATAC sites are significantly closer to experience-induced genes than to experience-suppressed genes. *P*-value from two-sided Kolmogorov-Smirnov (K-S) test. **g**, Line plots of mean GR CUT&RUN CPM in P14 GR-Con and GR-KO astrocytes (*n* = 2 per condition) at experience-induced astrocyte GR sites ±1 kb. **h**, GR CUT&RUN for individual astrocyte GR-Con and GR-KO replicates at *Aldoc* (left) and *Hif3a* (right) loci (see also Fig. 2e).

To determine whether light promotes GR recruitment to astrocyte chromatin to regulate gene expression, we labelled V1 astrocytes at birth with an astrocyte-specific adeno-associated virus (AAV)^28^ expressing a nuclear membrane-targeting GFP (eGFP-KASH), reared the mice in DR or NR conditions, and isolated GFP-labelled nuclei at P14 to profile GR genomic binding by CUT&RUN, a low-input transcription factor profiling method that we validated in vitro (Extended Data Figs. 7a–e and  8a,b). With this approach, we detected 6,274 light-induced GR binding sites (Fig. 2d, Extended Data Fig. 8c–e and Supplementary Table 6), which included recruitment to canonical GR target genes, such as the metallothioneins *Mt1* and *Mt2*^29^, as well as loci specific to astrocytes that are not bound by GR in peripheral mouse tissues, such as *Aldoc* and *Cables1* (Fig. 2e and Extended Data Fig. 8f). Comparison of these light-induced GR binding sites to our V1 multiome dataset revealed preferential GR recruitment to light-induced genes and chromatin regions that are activated in astrocytes between P14 and P21 (Fig. 2f and Extended Data Fig. 9a–f), suggesting a direct role for GR in visual experience-dependent astrocytic gene regulation. To validate the specificity of GR binding to these sites, we delivered astrocyte-specific AAVs expressing either Cre (Cre-T2A-KASH-GFP (GR-KO)) or a catalytically-inactive ΔCre control (ΔCre-T2A-KASH-GFP (GR-con)) into V1 of neonatal floxed GR mice (*Nr3c1*^*fl/fl*^, hereafter *GR*^*fl/fl*^) and repeated GR CUT&RUN on GFP-labelled astrocyte nuclei at P14. We found that eliminating GR in astrocytes substantially attenuated the recruitment of GR to these sites, indicating that our CUT&RUN analysis identified bona fide GR binding within astrocyte DNA (Fig. 2e and Extended Data Figs. 8g–m and 9g,h).

**Extended Data Fig. 10:**
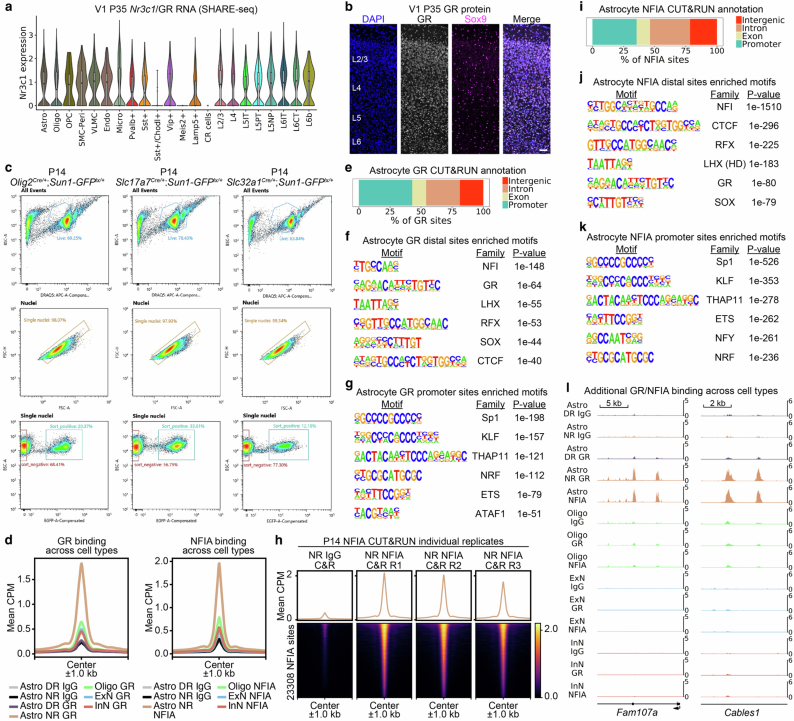
Comparison of GR and NFIA genomic binding across V1 cortical cell classes. **a**, P35 *Nr3c1*/GR expression in SHARE-seq experiment. Cell number: Astrocytes *n* = 1,518, Oligo *n* = 1741, OPCs *n* = 43, SMC-Peri *n* = 116, VLMCs *n* = 303, Endo *n* = 214, Micro *n* = 876, Pvalb+ *n* = 732, Sst+ *n* = 417, Sst + /Chodl+ *n* = 13, Vip+ *n* = 309, Meis2+ *n* = 9, Lamp5+ *n* = 223, CR cells *n* = 1, L2/3 *n* = 2744, L4 *n* = 2,186, L5IT *n* = 500, L5PT *n* = 686, L5NP *n* = 264, L6IT *n* = 899, L6CT *n* = 1,777, L6b *n* = 157. Boxplot centre, median; box boundaries, first and third quartiles; whiskers, 1.5 × IQR from boundaries. **b**, Representative immunofluorescent images of DAPI (left), GR (middle left), Sox9 (middle right), and merged channels (right) in V1 at P35. Scale, 50 µm. Experiment was performed once. **c**, FACS gating strategy to isolate GFP+ singlet nuclei from oligodendrocytes (left, *Olig2*^*Cre/+*^; *Sun1-GFP*^*lx/+*^), excitatory neurons (middle, *Slc17a7*^*Cre/+*^; *Sun1-GFP*^*lx/+*^), and inhibitory neurons (right, *Slc32a1*^*Cre/+*^*; Sun1-GFP*^*lx/+*^) for CUT&RUN. Top, DRAQ5+ nuclei gate; middle, singlet gate; bottom, GFP+ nuclei gate. **d**, Line plots of GR (left) or NFIA (right) mean CUT&RUN CPM in astrocytes (Astro), oligodendrocytes (Oligo), excitatory neurons (ExN), and inhibitory neurons (InN) at astrocyte GR (6,274) or NFIA (23,308) sites ±1 kb. **e**, Genomic location of astrocyte GR sites. **f-g**, Top unique enriched motifs (HOMER hypergeometric test, one-sided, *P* < 0.05) in distal (**f**) and promoter (**g**) astrocyte GR sites. **h**, Line plots (top) and heat maps (bottom) of mean NR IgG and NFIA CUT&RUN CPM ± 1 kb around consensus astrocyte NFIA peaks (MACS2, *q* < 0.01, in 2 of 3 replicates) in GFP+ nuclei. Color scale indicates CPM. **i**, Genomic location of astrocyte NFIA sites. **j-k**, Top unique enriched motifs (HOMER hypergeometric test, one-sided, *P* < 0.05) in distal (**j**) and promoter (**k**) astrocyte NFIA sites. **l**, Example GR and NFIA co-bound loci.

Although most cortical cell types express GR mRNA and protein (Extended Data Fig. 10a,b), whether GR preferentially binds DNA within specific cell types in the brain is not known. To assess the extent of GR genomic recruitment in astrocytes relative to other V1 cell types, we profiled GR binding in the non-astrocyte, GFP-negative fraction, as well as in excitatory neurons, inhibitory neurons and oligodendrocytes that were isolated from three separate transgenic mouse lines in which these cell types were labelled with a nuclear membrane-localized GFP (Extended Data Fig. 10c). In each case, we detected a markedly reduced number of GR-bound loci compared to the number of sites identified in astrocytes (Fig. 2g and Extended Data Fig. 10d). Together, these findings indicate that light recruits GR to DNA predominantly in astrocytes relative to other cortical cell classes, although GR may still bind chromatin in sparse cell subtypes that were not detected in our analyses.

**Extended Data Fig. 11:**
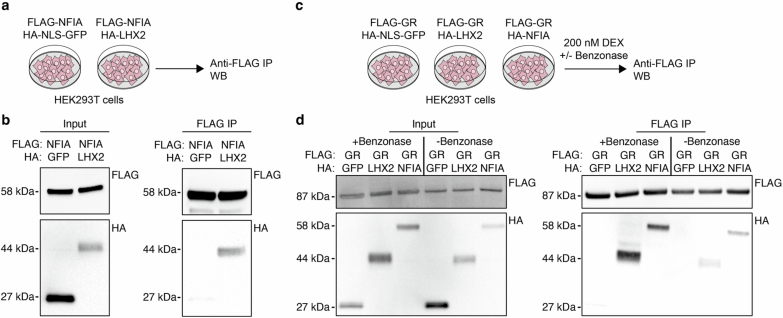
Immunoprecipitation of GR with NFIA and LHX2 in HEK293T cells. **a**, Experimental strategy for co-immunoprecipitation of FLAG-NFIA and HA-NLS-GFP or HA-LHX2 from co-transfected HEK293T cells. **b**, Input (left) or FLAG immunoprecipitation (IP) (right) fractions from cells over-expressing FLAG-NFIA/HA-NLS-GFP or FLAG-NFIA/HA-LHX2 immunoblotted for FLAG (top) or HA (bottom). Immunoprecipitation experiment was performed once. For gel source, see Supplementary Fig. 1. **c**, Experimental strategy for co-immunoprecipitation of FLAG-GR and HA-NLS-GFP, FLAG-GR and HA-LHX2, or FLAG-GR and HA-NFIA from co-transfected HEK293T cells. Cells were stimulated with 200 nM DEX for 2 hr, and protein lysates were extracted with or without benzonase. **d**, Input (left) or FLAG IP (right) fractions extracted with (+) or without (−) benzonase from cells over-expressing FLAG-GR/HA-NLS-GFP, FLAG-GR/HA-LHX2, or FLAG-GR/HA-NFIA immunoblotted for FLAG (top) or HA (bottom). Immunoprecipitation experiment was performed once. For gel source, see Supplementary Fig. 2.

GR is known to interact with chromatin through binding modes that often involve tethering or cooperativity with other transcription factors^24^. Such interactions enable GR, which is ubiquitously expressed, to drive distinct transcriptional responses across different cell types^30^. To predict whether GR binds with other transcription factors in astrocytes, we performed transcription factor motif analysis of GR-bound loci and identified enrichment of the binding motifs for gliogenic transcription factors belonging to the NFI and LIM-homeobox (LHX) families as well as the canonical GR motif (Extended Data Fig. 10e–g). Prior work has identified a transcriptional complex composed of NFIA and LHX2 that controls the morphogenesis of cortical astrocytes^31^. To investigate whether GR binds chromatin near components of this complex, we profiled NFIA binding in astrocytes at P14 and identified extensive genomic co-localization of GR and NFIA (Fig. 2h,i, Extended Data Fig. 10h–l and Supplementary Table 7) that was largely absent from other cell types (Fig. 2g and Extended Data Fig. 10d). In addition, GR co-immunoprecipitates with NFIA and LHX2 expressed in HEK293T cells (Extended Data Fig. 11a–d and Supplementary Figs. 1 and 2). Collectively, these results support a model in which GR primarily binds astrocyte chromatin together with the lineage-defining transcription factor NFIA.

## GR regulates postnatal astrocyte maturation

**Fig. 3:**
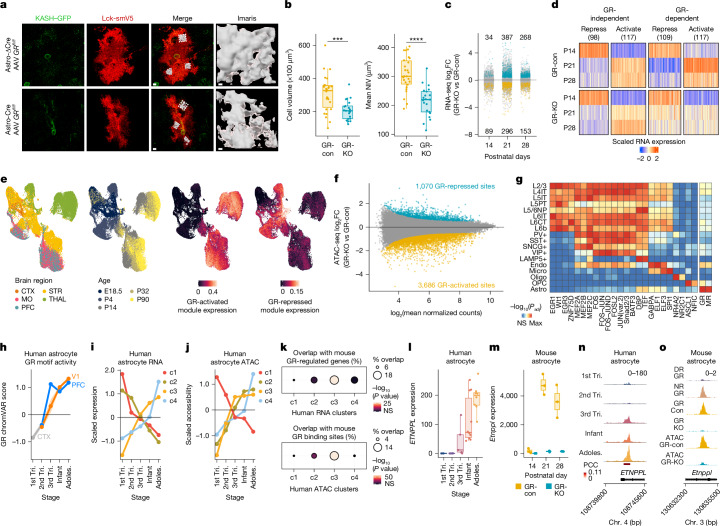
GR regulates astrocyte maturation. **a**, Representative immunofluorescent images of KASH–GFP, Lck-smV5 and merged channels for GR-con (Astro-ΔCre *GR*^*fl/fl*^) and GR-KO (Astro-Cre *GR*^*fl/fl*^) astrocytes. Right, Imaris reconstruction of outlined region. Scale bars: 5 µm (left), 1 µm (right). **b**, Astrocyte cell volume (left) and neuropil infiltration volume (NIV) (right) of GR-con (*n* = 26 cells, 3 mice) and GR-KO (*n* = 21 cells, 3 mice) astrocytes. Two-sided *t*-test. Cell volume: *P* = 3.5 × 10^−4^; NIV: *P* = 5.6 × 10^−7^. **c**, Number of differentially expressed genes between GR-con and GR-KO astrocytes (DESeq2 Wald test, two-sided, *P*_adj_ < 0.05). *n* = 3 replicates per condition. **d**, Heat map of average expression (column-scaled) of V1 astrocyte developmentally regulated genes (P21 GR-con versus P14 GR-con, DESeq2 Wald test, two-sided, *P*_adj_ < 0.05) grouped with hierarchical clustering. **e**, UMAPs of astrocytes from mouse cortex (CTX), motor cortex (MO), PFC, striatum (STR) and thalamus (THAL)^39^, coloured by (left to right): brain region; age; GR-activated gene module expression; and GR-repressed gene module expression. **f**, P21 astrocyte GR-dependent ATAC sites (DiffBind edgeR QLFTest, two-sided, *P*_adj_ < 0.05). **g**, Heat map of −log_10_(*P*_adj_) values (row-scaled) for top differentially accessible chromVAR transcription factor motif scores between human adolescence and first trimester cell types^42^ (MAST zlm likelihood ratio test, two-sided, *P*_adj_ < 0.05). **h**, GR chromVAR score (mean ± s.e.m.) in human whole cortex, PFC (blue) and V1 (orange) astrocytes across development. Adoles., adolescence; Tri., trimester. **i**,**j**, Scaled expression (**i**) or accessibility (**j**) (mean ± s.e.m.) of human astrocyte developmentally regulated genes (devRNA) or ATAC sites (devATAC), respectively, grouped by hierarchical clustering (pseudobulk DESeq2 Wald test, two-sided; RNA: *P*_adj_ < 0.01, ATAC: *P*_adj_ < 0.05). **k**, Top, per cent overlap between human devRNA clusters and mouse astrocyte GR-dependent genes. Bottom, per cent overlap between human devATAC clusters and mouse GR sites. One-sided Fisher’s exact test, coloured by −log_10_(*P* value). **l**, *ETNPPL* expression in human cortical astrocytes. 1st trimester *n* = 4 samples, 2nd trimester *n* = 11 samples, 3rd trimester *n* = 5 samples, infant *n* = 10 samples, adolescence *n* = 8 samples. **m**, *Etnppl* expression in mouse V1 GR-con and GR-KO astrocytes. *n* = 3 samples per condition. **n**, Human astrocyte ATAC signal at *ETNPPL*. Peak colour indicates Pearson’s correlation coefficient (PCC) between peak accessibility and gene expression. **o**, Mouse astrocyte GR binding and ATAC signal at conserved *Etnppl* site. Source data

**Extended Data Fig. 12:**
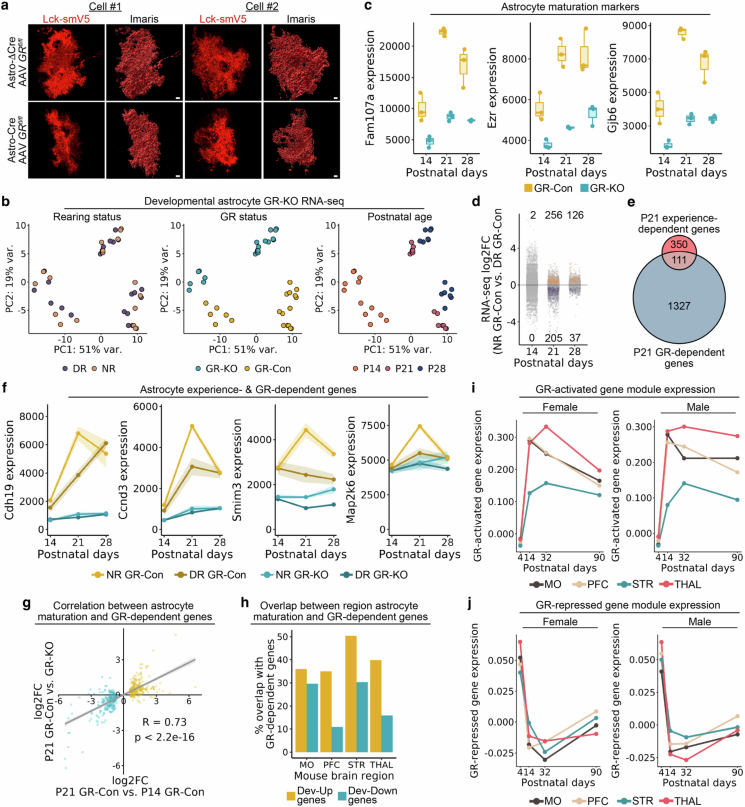
Additional analysis of astrocyte volume and characterization of GR-dependent gene expression across development and brain regions. **a**, Representative immunofluorescent images of Lck-smV5 (left) and Imaris 3D reconstruction (right) for two example GR-Con (Astro-ΔCre AAV *GR*^*fl/fl*^; top) and GR-KO (Astro-Cre AAV *GR*^*fl/fl*^; bottom) astrocytes. Cell #1 is the same as in Fig. 3a. Scale, 5 µm. **b**, PCA of RNA-seq samples (P14 NR GR-Con *n* = 3, P14 NR GR-KO *n* = 3, P14 DR GR-Con *n* = 3, P14 DR GR-KO *n* = 3, P21 NR GR-Con *n* = 3, P21 NR GR-KO *n* = 3, P21 DR GR-Con *n* = 4, P21 DR GR-KO *n* = 4, P28 NR GR-Con *n* = 3, P28 NR GR-KO *n* = 3, P28 DR GR-Con *n* = 4, P28 DR GR-KO *n* = 4). **c**, Normalized expression of astrocyte markers downregulated by GR-KO (DESeq2 Wald test, two-sided, *P*_adj_ < 0.05). *n* = 3 samples per condition. Boxplot centre, median; box boundaries, first and third quartiles; whiskers, 1.5 × IQR from boundaries. **d**, Differentially expressed genes (DESeq2 Wald test, two-sided, *P*_adj_ < 0.05) between NR and DR V1 GR-Con astrocytes at P14, P21, and P28. **e**, Overlap between P21 astrocyte GR-dependent genes and experience-dependent genes. **f**, Mean expression ± s.e.m. of select experience- and GR-dependent genes in astrocytes. **g**, Two-sided Pearson correlation ± 95% confidence interval of log2FC values for age-dependent genes (P21 vs. P14 GR-Con) and log2FC values for GR-dependent genes (P21 GR-KO vs.GR-Con). *P* = 2.16 × 10^−74^. **h**, Per cent overlap between developmentally upregulated (devUp) and developmentally downregulated (devDown) genes (P32 vs. P4 astrocytes, DESeq2 Wald test, two-sided, *P*_adj_ < 0.05) from different brain areas and GR-dependent genes detected in V1. **i-j**, Mean module score expression ± s.e.m. of V1 GR-activated genes (**i**) and GR-repressed genes (**j**) in astrocytes from the thalamus (THAL), PFC, motor cortex (MO), and striatum (STR)^39^ of female (left) and male (right) mice.

In early postnatal life, astrocytes undergo a series of morphological, electrophysiological and metabolic adaptations that sustain the optimal functioning of adult neural circuits^32^. Although the initial gene regulatory cascades underlying embryonic gliogenesis have been identified^33,34^, the specific factors that control postnatal astrocyte maturation remain poorly defined^35^. Considering the timing of CORT production and the widespread co-binding of GR and NFIA to DNA—and noting the ubiquitous expression of GR in V1 astrocytes (Extended Data Fig. 8l,m)—we reasoned that GR activation might specify the terminal maturation of astrocytes. To begin to address this possibility, we explored whether GR regulates the elaboration of astrocyte processes, which occurs after eye opening^36^ and is a hallmark of a mature astrocyte identity^31^. We co-delivered astrocyte-specific AAVs expressing either Cre (GR-KO) or ΔCre (GR-con) along with a second astrocyte-specific AAV expressing a membrane-targeted V5 reporter^28^ into V1 of *GR*^*fl/fl*^ mice at birth, enabling subsequent 3D reconstruction of astrocyte membranes in the presence or absence of GR. We found that eliminating GR from astrocytes reduces the volume of astrocyte territories as well as the density of processes infiltrating the neuropil at P21 (Fig. 3a,b and Extended Data Fig. 12a), consistent with a role for GR signalling in the morphological maturation of astrocytes.

We next tested whether GR mediates the widespread gene expression changes that emerge in astrocytes across early postnatal life^35,37^. As described above, we generated GR-KO and GR-con V1 astrocytes through stereotactic delivery of Cre- or ΔCre-expressing AAVs into neonatal *GR*^*fl/fl*^ mice, raised mice in NR or DR conditions, and measured the gene expression profiles of astrocyte nuclei at P14, P21 and P28 in the presence or absence of GR (Extended Data Fig. 12b). This analysis identified hundreds of GR-regulated genes that increase or decrease expression across postnatal development following the postnatal rise in circulating CORT (Fig. 3c, Extended Data Fig. 12c and Supplementary Table 8), including classic markers of adult astrocytes, such as *Aldh1a1*, *Ezr* and *Fam107a*^31,35,38^, as well as *Gjb6*, which has previously been shown to specify astrocyte maturation^12^. Consistent with our multiome dataset, GR-KO also occluded the induction of a subset of light-dependent genes at P21 (Extended Data Fig. 12d–f), demonstrating GR contributes to the effects of light on astrocyte gene expression.

We used hierarchical clustering to quantify the extent to which GR regulates astrocyte maturation and detected two programs, comprising approximately 50% of all developmentally regulated genes, that require GR for either their induction or repression (Fig. 3d and Extended Data Fig. 12g). An additional comparison of the GR-regulated genes identified in V1 astrocytes to astrocyte maturation genes detected in other regions of the mouse brain^39^, including striatum, thalamus, prefrontal cortex (PFC) and motor cortex, revealed a high degree of overlap that was shared across female and male mice (Fig. 3e and Extended Data Fig. 12h–j), suggesting that GR signalling may coordinate astrocyte maturation throughout the brain.

**Extended Data Fig. 13:**
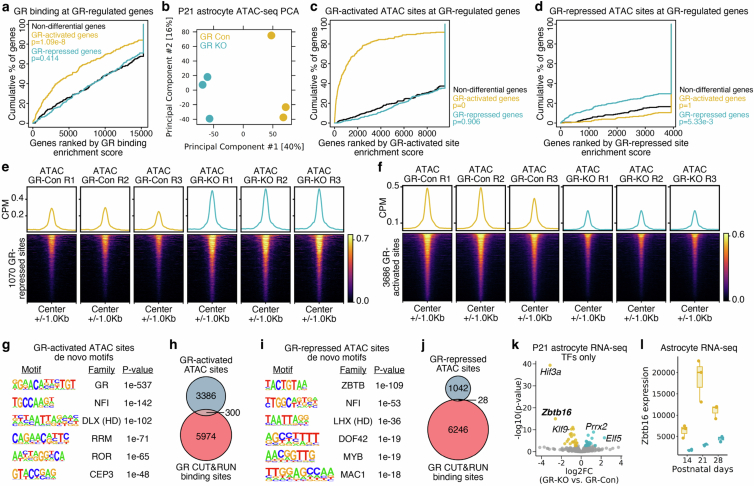
Influence of GR signalling on astrocyte chromatin accessibility. **a**, BETA enrichment of astrocyte GR binding sites ±200 kb around P21 astrocyte GR-activated and GR-repressed genes identified by RNA-seq relative to a background of non-differential, expressed genes. *P*-value from two-sided K-S test. **b**, PCA of astrocyte GR-Con and GR-KO P21 ATAC samples (*n* = 3 per condition). **c-d**, BETA enrichment of astrocyte GR-activated (**c**) and GR-repressed (**d**) ATAC sites ±200 kb around P21 astrocyte GR-activated and GR-repressed genes identified by RNA-seq relative to a background of non-differential, expressed genes. *P*-value from two-sided K-S test. **e-f**, Line plots (top) and heat maps (bottom) of mean ATAC counts per million (CPM) ± 1 kb around GR-activated ATAC sites (**c**) and GR-repressed ATAC sites (**d**) (see also Fig. 3f). **g**, Top de novo motifs enriched in GR-activated sites (HOMER hypergeometric test, one-sided, *P* < 0.05). **h**, Overlap between GR-activated ATAC sites and astrocyte GR binding sites. **i**, Top de novo motifs enriched in GR-repressed sites (HOMER hypergeometric test, one-sided, *P* < 0.05). **j**, Overlap between GR-repressed ATAC sites and astrocyte GR binding sites. **k**, Volcano plot of GR-dependent TFs (see also Fig. 3c) in P21 V1 astrocytes (DESeq2 Wald test, two-sided, *P*_adj_ < 0.05). **l**, Mean, normalized expression of *Zbtb16* in GR-Con and GR-KO astrocytes across postnatal development. *n* = 3 samples per condition. Boxplot centre, median; box boundaries, first and third quartiles; whiskers, 1.5 × IQR from boundaries.

Examining the relationship between astrocyte GR-regulated genes and the location of GR binding sites identified here, we noted that GR preferentially localizes near GR-activated genes (that is, those that are downregulated with GR-KO) rather than GR-repressed genes (those that are upregulated with GR-KO) (Extended Data Fig. 13a). This finding suggests that GR may regulate the expression of other transcription factors that mediate gene repression in astrocytes. To understand how GR broadly affects chromatin state, and to identify potential transcriptional repressors that are regulated by GR signalling, we performed ATAC with sequencing (ATAC-seq) on GR-KO and GR-con astrocytes at P21 and identified regions of chromatin that either open or close in the absence of GR (Fig. 3f, Extended Data Fig. 13b and Supplementary Table 9). Consistent with our CUT&RUN and RNA sequencing (RNA-seq) data, GR-activated (that is, closed with GR-KO) sites primarily localized to GR-activated genes and contained the GR binding motif, whereas GR-repressed (opened with GR-KO) sites occurred near GR-repressed genes but lacked GR binding (Extended Data Fig. 13c–j). Instead, GR-repressed sites were enriched for DNA binding motifs for zinc-finger and BTB domain-containing (ZBTB) transcription factors (Extended Data Fig. 13i). Notably, the transcriptional repressor *Zbtb16* emerged as one of the most downregulated transcription factors upon GR elimination (Extended Data Fig. 13k,l), and it has been previously identified as a canonical GR target gene outside of astrocytes^40^. Together, these findings suggest a model in which GR directly activates transcription through DNA binding yet indirectly inhibits transcription by activating repressors that inactivate a set of genes in maturing astrocytes.

**Extended Data Fig. 14:**
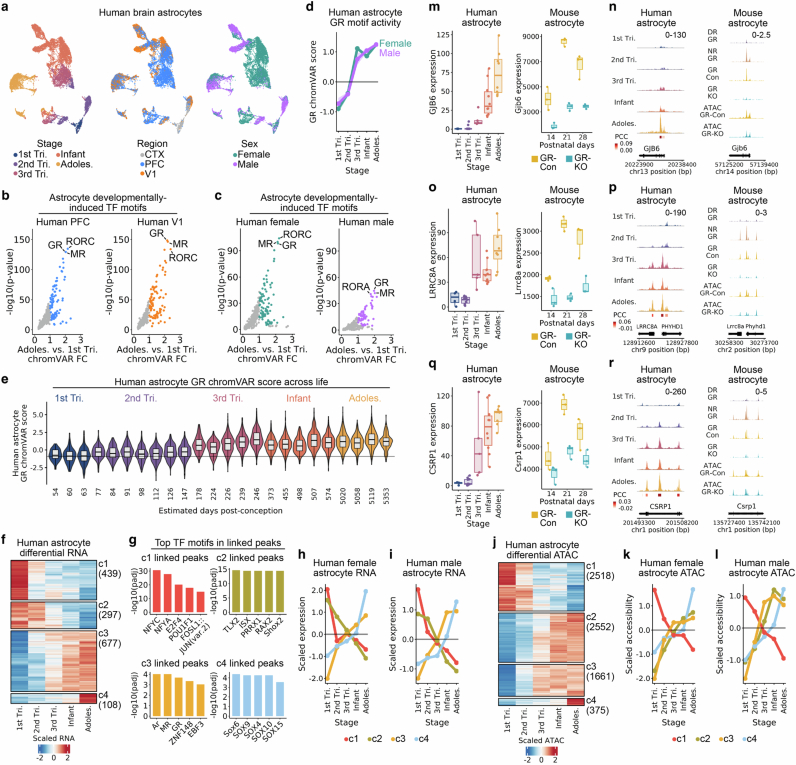
Identification of GR-dependent gene regulatory program across human astrocyte maturation. **a**, UMAPs of human cortical astrocyte multiome data^42^, colored by age (left), brain region (middle), and sex (right). **b-c**, Volcano plots of chromVAR TF motif scores between adolescent and first trimester astrocytes (MAST zlm likelihood-ratio test, *P*_adj_ < 0.05) from (**b**) PFC (left) and V1 (right) (**c**) and females (left) and males (right). **d**, Mean GR chromVAR score ± s.e.m. in human astrocytes from females (teal) and males (purple). **e**, GR chromVAR score in human astrocytes across development. Nuclei number for each estimated day post-conception: 54 *n* = 30, 60 *n* = 110, 63 *n* = 238, 77 *n* = 106, 84 *n* = 162, 91 *n* = 130, 98 *n* = 206, 112 *n* = 222, 126 *n* = 275, 147 *n* = 345, 178 *n* = 124, 224 *n* = 74, 226 *n* = 752, 239 *n* = 908, 246 *n* = 539, 373 *n* = 535, 455 *n* = 2,342, 498 *n* = 254, 507 *n* = 428, 574 *n* = 1,737, 5,020 *n* = 832, 5,058 *n* = 726, 5,119 *n* = 707, 5,353 *n* = 203. Boxplot centre, median; box boundaries, first and third quartiles; whiskers, 1.5 × IQR from boundaries. **f**, Scaled, normalized expression of human astrocyte developmentally regulated genes (devRNA) (adolescent vs. first trimester, pseudobulk DESeq2 Wald test, two-sided, *P*_adj_ < 0.05) grouped by hierarchical clustering. Cluster c2 genes significantly overlap mouse GR-dependent genes (see also Fig. 3i). **g**, Top enriched motifs (Hypergeometric test, two-sided, *P*_adj_ < 0.05) within human astrocyte ATAC peaks correlated (Pearson correlation, two-sided*, P* < 0.001) with genes belonging to each devRNA cluster. **h-i**, Mean, scaled expression ± s.e.m. of human astrocyte devRNA gene clusters in females (**h**) and males (**i**). **j**, Scaled, normalized accessibility of human astrocyte developmentally regulated ATAC sites (devATAC) (adolescent vs. first trimester, pseudobulk DESeq2 Wald test, two-sided, *P*_adj_ < 0.01) grouped by hierarchical clustering (see also Fig. 3j). **k-l**, Mean, scaled accessibility ± s.e.m. of human astrocyte devRNA gene clusters in females (**k**) and males (**l**). **m-r**, Additional developmentally induced genes and regulatory elements conserved between human and mouse astrocytes (see also Fig. 3l–o). Human astrocyte 1st Tri. *n* = 4 samples, 2nd Tri. *n* = 11 samples, 3rd Tri. *n* = 5 samples, Infant *n* = 10 samples, Adoles. *n* = 8 samples; mouse astrocyte *n* = 3 samples per condition. Boxplot centre, median; box boundaries, first and third quartiles; whiskers, 1.5 × IQR from boundaries. Peak color indicates PCC between ATAC peak accessibility and gene expression.

Human astrocytes mature progressively during late gestation and early postnatal life, although throughout this process, they acquire more extensive and complex morphologies than mouse astrocytes, which may support higher cognitive functions^37,41^. To assess whether the relationship between GR signalling and astrocyte maturation may extend to humans, we reanalysed a single-nucleus multiome dataset of the human neocortex across five developmental stages spanning gestation, infancy and adolescence^42^ (Extended Data Fig. 14a). As is the case for the developing postnatal mouse, we found that the transcription factor binding motifs that gain accessibility in V1 and PFC during human astrocyte maturation differed from those that gained accessibility in other cell types. In all human cortical neuronal subtypes and most non-neuronal cell types, binding motifs for activity-regulated transcription factors, including AP-1, MEF2 and EGR proteins, increased accessibility the most between the first trimester in utero and in adolescence, whereas in astrocytes, the binding motif for GR gained greater accessibility compared to other transcription factors in both female and male individuals (Fig. 3g and Extended Data Fig. 14b,c). Further analysis revealed a progressive opening of the GR motif that initiates during the third trimester in utero (Fig. 3h and Extended Data Fig. 14d,e), consistent with the proposed role of glucocorticoids in preparing the mammalian fetus for extrauterine life^43^.

To determine whether human astrocyte maturation is accompanied by the induction of GR-regulated genes and chromatin regions, we identified genes and chromatin regions that change expression or accessibility, respectively, across human astrocyte development and categorized them separately using hierarchical clustering. This analysis revealed a gene expression program that progressively increases across human development, peaks in adolescence, and includes many genes that are regulated by GR in mouse V1 astrocytes (Fig. 3i,k and Extended Data Fig. 14f–i). We also independently identified a group of chromatin regions that gain accessibility across development (Fig. 3j,k and Extended Data Fig. 14j–l) and contain conserved GR binding sites found in mouse V1 astrocytes, including putative enhancers for the mature astrocyte markers *ETNPPL* and *GJB6* (Fig. 3l–o and Extended Data Fig. 14m–r). Collectively, our findings indicate that GR regulates the maturation of mouse astrocytes and may likewise have an important role in the maturation of human astrocytes.

## Astrocyte GR promotes neural circuit maturation

**Extended Data Fig. 15:**
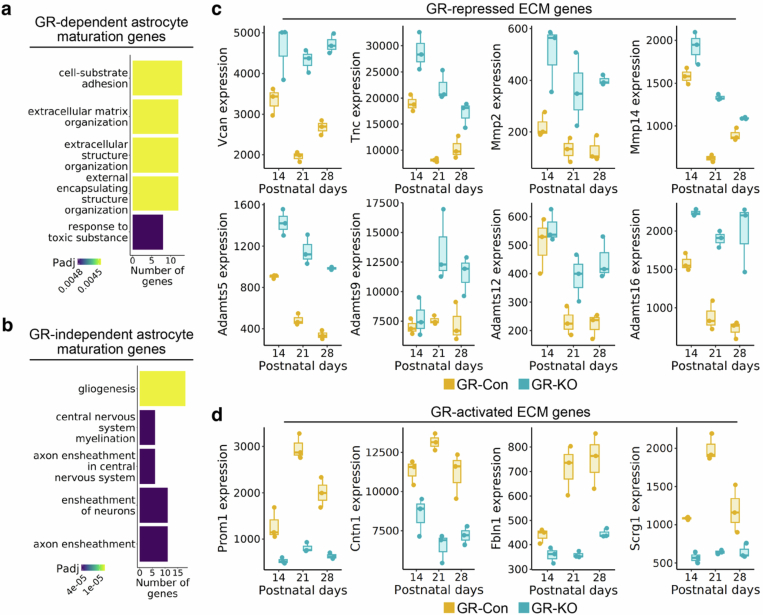
Astrocyte GR signalling regulates an ECM gene program. **a-b**, Top GO terms (clusterProfiler enrichGO hypergeometric test, one-sided, *P*_adj_ < 0.05) enriched in (**a**) astrocyte GR-dependent maturation genes and (**b**) astrocyte GR-independent maturation genes (see also Fig. 3d). **c**, Normalized expression of immature glycoproteins (*Vcan*, *Tnc*) and metalloproteinases (*Mmp2*, *Mmp14*, *Adamts5*, *Adamts9*, *Adamts12*, *Adamts16*) induced upon GR-KO in V1 astrocytes across postnatal development. *n* = 3 samples per condition. Boxplot centre, median; box boundaries, first and third quartiles; whiskers, 1.5 × IQR from boundaries. **d**, Normalized expression of mature cell-surface and secreted ECM proteins (*Prom1*, *Cntn1*, *Fbln1*, *Scrg1*) downregulated upon GR-KO in V1 astrocytes across postnatal development. *n* = 3 samples per condition. Boxplot centre, median; box boundaries, first and third quartiles; whiskers, 1.5 × IQR from boundaries.

To determine how astrocyte GR signalling might affect neighbouring cell types during V1 development, we conducted a Gene Ontology search of GR-dependent astrocyte maturation genes in mouse V1. This analysis revealed a significant number of GR-regulated genes in astrocytes that encode proteins involved in extracellular matrix (ECM) organization (Extended Data Fig. 15a,b). Specifically, GR disruption in astrocytes led to a bidirectional transcriptional re-programming of ECM proteins, a process that is known to occur during astrocyte maturation^32^, upregulating immature glycoprotein genes (*Tnc* and *Vcan*) and ECM-degrading metalloproteinase genes (*Adamts5*, *Adamts9*, *Adamts12*, *Adamts16*, *Mmp2* and *Mmp14*), and downregulating factors associated with a mature ECM (*Cntn1*, *Fbln1*, *Prom1* and *Scrg1*) (Extended Data Fig. 15c,d). These findings suggest that astrocyte GR signalling may control the timing of ECM formation during postnatal development.

**Fig. 4:**
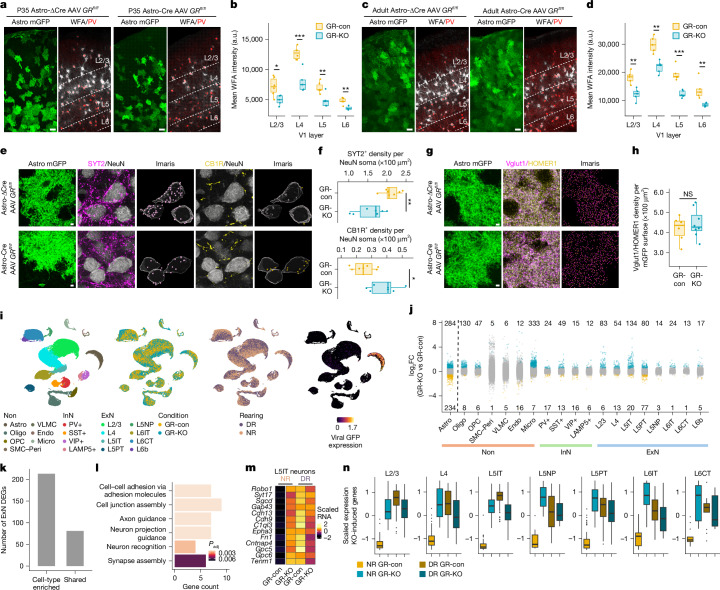
Astrocyte GR signalling promotes cortical maturation. **a**–**d**, Representative immunofluorescent images of astrocyte mGFP, and *Wisteria floribunda* lectin (WFA) and PV in P35 (**a**) and adult (**c**) Astro-ΔCre AAV *GR*^*fl/fl*^ (GR-con) (left) and Astro-Cre AAV *GR*^*fl/fl*^ (GR-KO) (right) mice, and mean WFA intensity on PV somas in P35 (**b**) and adult (**d**) GR-con (P35: *n* = 6, adult *n* = 5) and GR-KO (P35: *n* = 5, adult: *n* = 5) mice. Two-sided *t*-test. P35 L2/3: *P* = 0.017; P35 L4: *P* = 3.5 × 10^−4^; P35 L5: *P* = 7.7 × 10^−3^; P35 L6: *P* = 1.8 × 10^−3^; adult L2/3: *P* = 2.2 × 10^−3^; adult L4: *P* = 1.1 × 10^−3^; adult L5: *P* = 4.9 × 10^−4^; adult L6: *P* = 8.8 × 10^−3^. Scale bars, 50 µm. a.u., arbitrary units. **e**, Representative immunofluorescent images of L5 astrocytes in P35 GR-con (top) and GR-KO (bottom) mice. Left to right: mGFP; SYT2 and NeuN; SYT2 and NeuN Imaris reconstruction; CB1R and NeuN; and CB1R and NeuN Imaris reconstruction. Scale bars, 3 µm. **f**, Median densities of SYT2 (top) and CB1R (bottom) puncta on L5 NeuN somas in P35 GR-con (*n* = 6) and GR-KO (*n* = 7) mice. Two-sided *t*-test; SYT2: *P* = 8.5 × 10^−3^; CB1R: *P* = 0.01. **g**, Representative immunofluorescent images of astrocyte mGFP (left), Vglut1 and HOMER1 (middle) and Vglut1 and HOMER1 Imaris reconstruction (right) in P35 GR-con (top) and GR-KO (bottom) mice. Scale bars, 3 µm. **h**, Median densities of Vglut1^+^HOMER1^+^ puncta on mGFP surface in P35 GR-con (*n* = 6) and GR-KO (*n* = 8) mice. Two-sided *t*-test. **i**, UMAPs of P21 V1 astrocyte snRNA-seq (*n* = 2 mice per condition) coloured by (left to right): cell identity; astrocyte GR status; rearing condition; and GFP expression. **j**, Astrocyte GR-dependent genes across cell types (pseudobulk edgeR, likelihood ratio test, two-sided, *P*_adj_ < 0.05). **k**, Number of differentially expressed genes (DEGs) in excitatory neuron types following GR knockout in astrocytes, grouped as cell-type-specific or shared (at least two cell types). **l**, Top Gene Ontology terms (enrichGO hypergeometric test, one-sided, *P*_adj_ < 0.05) enriched among astrocyte GR-KO-induced genes shared across excitatory neurons. **m**, Row-scaled expression of axonal wiring genes in L5IT neurons across conditions (*n* = 2 per condition). **n**, Scaled mean expression of NR astrocyte GR-induced genes (pseudobulk edgeR, likelihood ratio test, two-sided, *P*_adj_ < 0.05) across NR GR-con, NR GR-KO, DR GR-con and DR GR-KO conditions by excitatory neuron type. Number of genes: L2/3: *n* = 83, L4: *n* = 54, L5IT: *n* = 134, L5NP: *n* = 14, L5PT: *n* = 80, L6IT: *n* = 24, L6CT: *n* = 13. Source data

**Extended Data Fig. 16:**
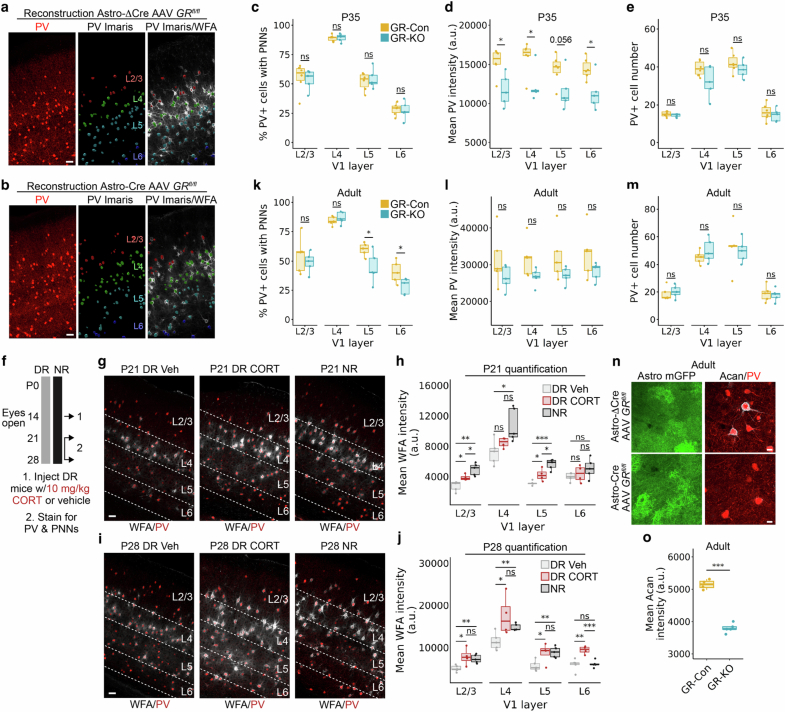
Effects of astrocyte GR signalling or CORT administration on PNNs. **a-b**, Representative immunofluorescent images of PV (left), PV Imaris reconstruction (middle), and PV Imaris reconstruction merged with WFA (right) in adult Astro-ΔCre AAV *GR*^*fl/fl*^ (GR-Con) (**a**) and Astro-Cre AAV *GR*^*fl/fl*^ (GR-KO) (**b**) mice. PV colored by V1 layer. Scale, 50 µm. **c-e**, Proportion of PV+ cells containing PNNs (**c**), mean PV intensity (a.u.) (**d**), and number of PV+ cells (**e**) in P35 astrocyte GR-Con (*n* = 6) and GR-KO (*n* = 5) animals. Two-sided *t*-test; *P < 0.05; ns, not significant. PV intensity: P35 L2/3 *P* = 0.011; P35 L4 *P* = 0.020; P35 L5 *P* = 0.056; P35 L6 *P* = 0.019. Boxplot centre, median; box boundaries, first and third quartiles; whiskers, 1.5 × IQR from boundaries. **f**, Experimental strategy for systemic delivery of CORT (10 mg/kg, i.p.) or vehicle (corn oil) to DR mice at P14. **g**, Representative immunofluorescent images of WFA (white) and PV (red) in P21 DR Veh (left), DR CORT (middle), and NR (right) animals. Scale, 50 µm. **h**, Mean WFA intensity (a.u.) on PV somas in P21 DR Veh (*n* = 5), P21 DR CORT (*n* = 4), and P21 NR (*n* = 5) animals. Two-sided *t*-test; ns, not significant; *P < 0.05, **P < 0.01, ***P < 0.001. L2/3 DR Veh vs. DR CORT *P* = 0.020; L2/3 DR CORT vs. NR *P* = 0.042; L2/3 DR Veh vs. NR *P* = 0.001; L4 DR Veh vs. DR CORT *P* = 0.190; L4 DR CORT vs. NR *P* = 0.087; L4 DR Veh vs. NR *P* = 0.019; L5 DR Veh vs. DR CORT *P* = 0.026; L5 DR CORT vs. NR *P* = 0.027; L5 DR Veh vs. NR *P* = 7.37 × 10^−5^; L6 DR Veh vs. DR CORT *P* = 0.488; L6 DR CORT vs. NR *P* = 0.401; L6 DR Veh vs. NR *P* = 0.106. Boxplot centre, median; box boundaries, first and third quartiles; whiskers, 1.5 × IQR from boundaries. **i**, Representative immunofluorescent images of WFA (white) and PV (red) in P28 DR Veh (left), DR CORT (middle), and NR (right) animals. Scale, 50 µm. **j**, Mean WFA intensity (a.u.) on PV somas in P28 DR Veh (*n* = 5), P28 DR CORT (*n* = 4), and P28 NR (*n* = 5) animals. Two-sided *t*-test; ns, not significant; *P < 0.05, **P < 0.01, ***P < 0.001. L2/3 DR Veh vs. DR CORT *P* = 0.025; L2/3 DR CORT vs. NR *P* = 0.640; L2/3 DR Veh vs. NR *P* = 0.004; L4 DR Veh vs. DR CORT *P* = 0.035; L4 DR CORT vs. NR *P* = 0.243; L4 DR Veh vs. NR *P* = 0.009; L5 DR Veh vs. DR CORT *P* = 0.046; L5 DR CORT vs. NR *P* = 0.850; L5 DR Veh vs. NR *P* = 0.004; L6 DR Veh vs. DR CORT *P* = 0.005; L6 DR CORT vs. NR *P* = 0.0008; L6 DR Veh vs. NR *P* = 0.806. Boxplot centre, median; box boundaries, first and third quartiles; whiskers, 1.5 × IQR from boundaries. **k-m**, Proportion of PV+ cells containing PNNs (**k**), mean PV intensity (a.u.) (**l**), and number of PV+ cells (**m**) in adult astrocyte GR-Con (*n* = 5) and GR-KO (*n* = 5) animals. Two-sided *t*-test; ns, not significant. *P < 0.05, L5 % PV+ cells with PNNs *P* = 0.044; L6 % PV+ cells with PNNs *P* = 0.049. Boxplot centre, median; box boundaries, first and third quartiles; whiskers, 1.5 × IQR from boundaries. **n**, Representative immunofluorescent images of L5 V1 astrocyte membrane-GFP (mGFP) (left) and aggrecan (Acan) and PV (right) in adult astrocyte GR-Con (top) and GR-KO (bottom) mice. Scale, 10 µm. **o**, Mean Acan intensity (a.u.) on L5 PV somas in adult GR-Con (*n* = 4) and GR-KO (*n* = 5) animals. Two-sided *t*-test; ***P < 0.01. *P* = 2.5 × 10^−6^. Boxplot centre, median; box boundaries, first and third quartiles; whiskers, 1.5 × IQR from boundaries.

Given the known role of the ECM in restraining cortical plasticity^44^, we next investigated the effect of astrocyte GR elimination on neural circuit maturation. We focused on the chondroitin sulfate proteoglycan (CSPG)-containing perineuronal nets (PNNs), which assemble on PV interneurons across postnatal development and function as a structural brake on ocular dominance plasticity (ODP)^45^. Eliminating GR in astrocytes following birth reduced the density of PNNs on PV interneurons across V1 cortical layers at P35 (Fig. 4a,b and Extended Data Fig. 16a,b), with the largest decreases occurring in L4 and L5. GR knockout in astrocytes also reduced PV expression without affecting the number of PV interneurons (Extended Data Fig. 16c–e).

We further investigated whether CORT itself regulates PNN development, and found that systemic CORT delivery around eye opening (P14) increased the density of PNNs in DR mice at P21 and P28, partially restoring levels to those observed in NR mice (Extended Data Fig. 16f–j). Because CORT levels cycle daily throughout adulthood, we further speculated that astrocyte GR signalling might be required to maintain PNNs in the mature brain. To test this idea, we deleted GR in adult V1 astrocytes and measured PNN density three weeks after AAV delivery. We found that levels of PNNs and aggrecan, a PV interneuron-specific CSPG that is required for PNN assembly^46^, are reduced in the absence of astrocyte GR (Fig. 4c,d and Extended Data Fig. 16k–o). Collectively, these findings indicate that astrocyte GR signalling contributes to the formation and maintenance of a mature ECM.

**Extended Data Fig. 17:**
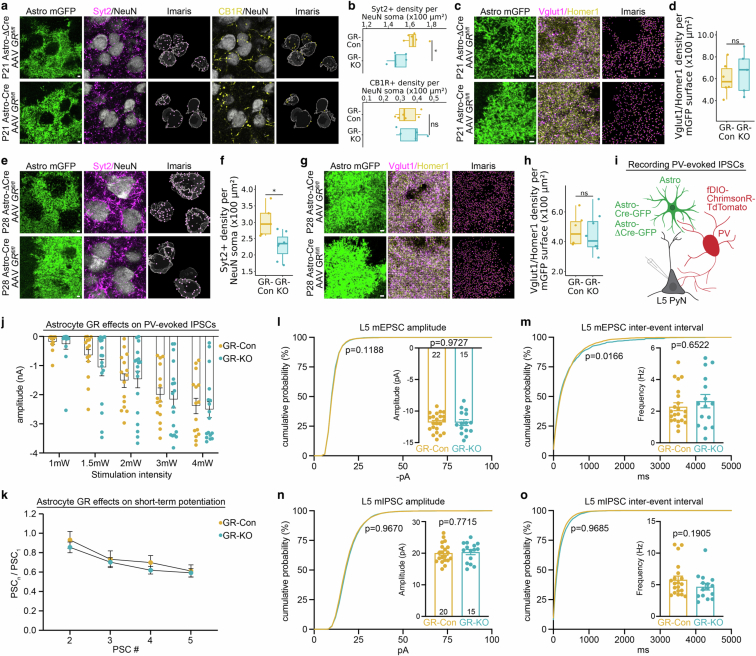
Effects of astrocyte GR signalling on excitatory and inhibitory synapses. **a**, Representative immunofluorescent images of V1 L5 astrocyte membrane-GFP (mGFP) (left), Syt2 and NeuN (middle-left), Syt2 and NeuN Imaris reconstruction (middle), CB1R and NeuN (middle-right), and CB1R and NeuN Imaris reconstruction (right) in P21 Astro-ΔCre AAV *GR*^*fl/fl*^ (GR-Con, top) and Astro-Cre AAV *GR*^*fl/fl*^ (GR-KO, bottom) animals. Scale, 3 µm. **b**, Median Syt2 (top) and CB1R (bottom) puncta density on L5 NeuN somas (x100µm^2^) in P21 GR-Con (*n* = 6) and GR-KO (*n* = 5) animals. Two-sided *t*-test; *P < 0.05; **P < 0.01. Syt2 *P* = 0.01; CB1R *P* = 0.56. Boxplot centre, median; box boundaries, first and third quartiles; whiskers, 1.5 × IQR from boundaries. **c**, Representative immunofluorescent images of V1 L5 astrocyte mGFP (left), Vglut1 and Homer1 (middle), and Vglut1 and Homer1 Imaris reconstruction (right) in P21 GR-Con (top) and GR-KO (bottom) animals. Scale, 3 µm. **d**, Median Vglut1 + /Homer1+ puncta density on mGFP surface (x100µm^2^) in P21 GR-Con (*n* = 6) and GR-KO (*n* = 5) animals. Two-sided *t*-test; ns, not significant. Boxplot centre, median; box boundaries, first and third quartiles; whiskers, 1.5 × IQR from boundaries. **e**, Representative immunofluorescent images of V1 L5 astrocyte mGFP (left), Syt2 and NeuN (middle), and Syt2 and NeuN Imaris 3D reconstruction (right) in P28 GR-Con (top row) and GR-KO (bottom row) animals. Scale, 3 µm. **f**, Quantification of median Syt2 puncta density on L5 NeuN somas (x100µm^2^) in P28 GR-Con (*n* = 5 animals) and GR-KO (*n* = 7 animals) mice. Two-tailed *t*-test; *P < 0.05. *P* = 0.02. Boxplot centre, median; box boundaries, first and third quartiles; whiskers, 1.5 × IQR from boundaries. **g**, Representative immunofluorescent images of V1 L5 astrocyte mGFP (left), Vglut1 and Homer1 (middle), and Vglut1 and Homer1 Imaris 3D reconstruction (right) in P28 GR-Con (top) and GR-KO (bottom) animals. Scale, 3 µm. **h**, Median Vglut1 + /Homer1+ puncta density on mGFP surface (x100µm^2^) in P28 GR-Con (*n* = 6) and GR-KO (*n* = 8) animals. Two-sided *t*-test; ns, not significant. Boxplot centre, median; box boundaries, first and third quartiles; whiskers, 1.5 × IQR from boundaries. **i**, Astro-ΔCre or Astro-Cre AAVs are co-injected with Flp-dependent ChrimsonR AAV into *PV*^*flp/flp*^; *GR*^*fl/fl*^ mice at birth to measure PV-evoked IPSCs on L5 pyramidal neurons (PyNs) in the presence or absence of GR signalling. **j**, PV-evoked IPSCs ± s.e.m. on L5 PyNs at different light stimulation intensities in P35 GR-Con (*n* = 15 cells/4 animals) and GR-KO (*n* = 17 cells/4 animals) animals. Two-way ANOVA test with Bonferroni correction; ns, not significant. **k**, Short-term potentiation (STP) ± SE of PV-evoked currents onto L5 pyramidal neurons using 5 stimulation pulses. PSC, post-synaptic current. Two-way ANOVA test with Bonferroni correction; P35 GR-Con *n* = 15 cells/4 animals, P35 GR-KO *n* = 17 cells/4 animals; not significant. **l-o**, Cumulative distribution plots for L5 pyramidal neuron mEPSC amplitude (**l**), mEPSC inter-event interval (**m**), mIPSC amplitude (**n**), and mIPSC inter-event interval (**o**) in P35 GR-Con (*n* = 22 cells/3 animals for mEPSCs, 20 cells/3 animals for mIPSCs) and GR-KO (*n* = 15 cells/3 animals for mEPSCs, 15 cells/3 animals for mIPSCs) mice. Insets show mean amplitude (pA) ± s.e.m. (**l**, **n**) or mean frequency (Hz) ± s.e.m. (**m**, **o**) per group. Cumulative distribution *P*-values from two-sided K-S test; inset p-values from two-sided *t*-test.

In addition to regulating ECM maturation, astrocytes instruct synapse formation and connectivity during brain development^36,47^. To determine whether astrocyte GR signalling regulates inhibitory or excitatory synapse number, we used immunofluorescent staining with antibodies to markers of presynaptic terminals derived from two major sources of cortical perisomatic inhibition—PV interneurons and cholecystokinin (CCK)-expressing interneurons^9,48^—and also stained with antibodies that recognize presynaptic and postsynaptic markers of excitatory synapses. We found that eliminating GR in astrocytes led to opposing effects on the density of PV and CCK perisomatic synapses formed onto neighbouring L5 neurons. Astrocyte GR disruption resulted in a decrease in PV synapse number (SYT2^+^ puncta) and an increase in CCK synapse number (CB1R^+^ puncta) onto L5 neurons (Fig. 4e,f) but had no effect on the density of intracortical excitatory synapses within the neuropil (Fig. 4g,h) at P35. The effect on PV synapse number preceded the increase in CCK synapse number, emerging at P21, when both CCK and excitatory synapse numbers were unaffected (Extended Data Fig. 17a–h). To further assess whether astrocyte GR regulates inhibitory currents specifically derived from PV neurons, we co-delivered astrocyte-specific Cre- or ΔCre-expressing AAVs containing a GFP reporter with a Flp-dependent red-shifted channelrhodopsin (ChrimsonR) into neonatal *PV*^*Flp/Flp*^*;GR*^*fl/fl*^ (*PV* is also known as *Pvalb*) mice and recorded PV-evoked inhibitory postsynaptic currents (IPSCs) onto neighbouring L5 pyramidal neurons at P35 within the astrocyte GFP-expressing region (Extended Data Fig. 17i). Consistent with prior work measuring the effects of PNN removal on synaptic physiology^49^, astrocyte GR elimination did not affect PV-evoked IPSCs (Extended Data Fig. 17j), PV-evoked paired-pulse ratio (Extended Data Fig. 17k), or the frequency or amplitude of miniature IPSCs or miniature excitatory postsynaptic currents (mEPSCs) onto pyramidal neurons (Extended Data Fig. 17l–o). Together, these findings suggest that astrocyte GR signalling regulates the balance of specific perisomatic inhibitory synaptic contacts during V1 development, although such presynaptic changes may be compensated by homeostatic strengthening at the postsynaptic membrane.

**Extended Data Fig. 18:**
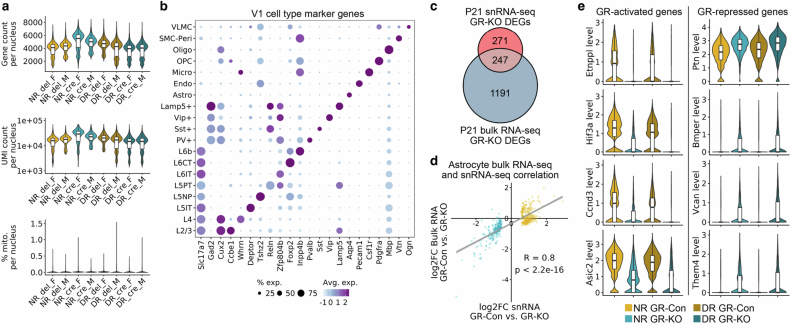
Additional analysis of astrocyte GR-KO snRNA-seq dataset. **a**, RNA QC for P21 astrocyte GR-KO and GR-Con snRNA-seq dataset; genes per nucleus (top), UMIs per nucleus (middle), and per cent mitochondrial UMIs per nucleus (bottom), colored by sample ID. Number of nuclei: NR_del_F *n* = 13,311, NR_del_M *n* = 13,144, NR_cre_F *n* = 9,517, NR_cre_M *n* = 12,126, DR_del_F *n* = 13,813, DR_del_M *n* = 15,161, DR_cre_F *n* = 13,852, DR_cre_M *n* = 11,406. Boxplot centre, median; box boundaries, first and third quartiles; whiskers, 1.5 × IQR from boundaries. **b**, Dotplot of marker gene expression across V1 cell types. **c**, Overlap between GR-dependent genes identified in P21 V1 snRNA-seq dataset (pseudobulk edgeR, LRT test, two-sided, *P*_adj_ < 0.05) (see also Fig. 4j) and GR-dependent genes identified in P21 V1 astrocyte bulk RNA-seq dataset (DESeq2 Wald test, two-sided, *P*_adj_ < 0.05). **d**, Two-sided Pearson correlation ± 95% confidence interval of log2FC values for P21 astrocyte GR-dependent genes detected by snRNA-seq and log2FC values for P21 astrocyte GR-dependent genes detected by bulk RNA-seq. *P* = 9.80 × 10^−114^. **e**, Normalized expression of GR-activated genes (left panels; *Etnppl*, *Hif3a*, *Ccnd3*, *Asic2*) and GR-repressed genes (right panels; *Ptn*, *Bmper*, *Vcan*, *Them4*) in P21 snRNA-seq dataset. Number of nuclei: NR GR-Con *n* = 2,337, NR GR-KO *n* = 1,904, DR GR-Con *n* = 2,787, DR GR-KO *n* = 2,458. Boxplot centre, median; box boundaries, first and third quartiles; whiskers, 1.5 × IQR from boundaries.

To further characterize the effects of astrocyte GR signalling on neural circuit maturation, we generated GR-KO and GR-con V1 astrocytes at birth by viral delivery of astrocyte-specific Cre or ΔCre-expressing AAVs into *GR*^*fl/fl*^ mice. We then raised mice under NR or DR conditions and isolated V1 nuclei at P21 for single-nucleus RNA-seq (snRNA-seq) (Fig. 4i and Extended Data Fig. 18a,b). Notably, analysis of AAV-derived *eGFP* expression across cortical cell types validated the astrocyte specificity of our AAVs (Fig. 4i), and comparison of GR-KO and GR-con astrocytes recapitulated many of the GR-dependent genes identified in our bulk astrocyte RNA-seq dataset (Extended Data Fig. 18c–e).

We next examined the effect of disrupting GR function in astrocytes on gene expression in other cortical cell types and detected hundreds of altered genes, primarily in excitatory neurons and other non-neuronal cell types (Fig. 4j and Supplementary Table 10), with the majority of genes being induced, rather than repressed, upon astrocyte GR disruption. Among the upregulated genes in neurons, we identified a core program involved in axonal sprouting that includes *Epha3*, *Gap43* and *Robo1* (Fig. 4k–m). This finding suggests that GR regulates genes in astrocytes that, in turn, signal to neurons to suppress axonal plasticity genes. This astrocyte-dependent suppression of neuronal gene expression might be critical for circuit maturation and the closure of critical periods, although additional functional evidence is needed to support this possibility. Consistent with this prediction, we find that a similar set of genes is upregulated in wild-type cortical excitatory neurons following DR (Fig. 4m,n), which has been shown to delay the physiological, transcriptional and structural maturation of V1^50–52^.

## Astrocyte GR restricts neuronal plasticity

**Fig. 5:**
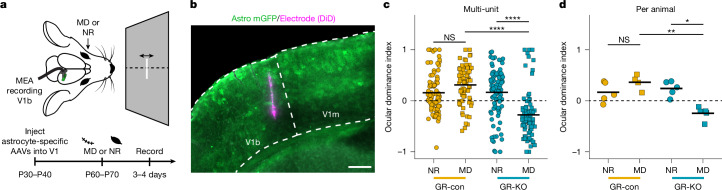
Astrocyte GR signalling restricts neuronal plasticity. **a**, Experimental strategy and timeline for ODP assay. MEA, microelectrode array. Adapted from BioRender; Gegenhuber, B. https://BioRender.com/245jg9r (2026). **b**, Representative immunofluorescent image of mGFP in V1 astrocytes and DiD-labelled electrode placement in an adult Astro-ΔCre AAV *GR*^*fl/fl*^ mouse. Scale bar, 200 µm. **c**,**d**, Ocular dominance index [(*R*_contra_ – *R*_ipsi_)/(*R*_contra_ + *R*_ipsi_)], where *R*_contra_ and *R*_ipsi_ indicate evoked visual responses (in Hz) for the contralateral and ipsilateral eyes, respectively, for individual multi-unit recordings (**c**) and averaged per mouse (**d**) in Astro-ΔCre AAV *GR*^*fl/fl*^ (GR-con) or Astro-Cre AAV *GR*^*fl/fl*^ (GR-KO) mice following NR or 3–4 days of MD (GR-con NR, *n* = 5 mice; GR-con MD, *n* = 4 mice; GR-KO NR, *n* = 5 mice; GR-KO MD, *n* = 5 mice). Kruskal–Wallis test with Dunn’s multiple comparisons correction; *P_adj_ < 0.05; **P_adj_ < 0.01; ***P_adj_ < 0.001; ****P_adj_ < 0.0001; multi-unit GR-con NR versus GR-con MD: *P*_adj_ = 0.0966; multi-unit GR-con MD versus GR-KO MD: *P*_adj_ ≤ 0.0001; multi-unit GR-KO NR versus GR-KO MD: *P*_adj_ ≤ 0.0001; animal GR-con NR versus GR-con MD *P*_adj_ ≥ 0.9999; animal GR-con MD versus GR-KO MD: *P*_adj_ = 0.0070; animal GR-KO NR versus GR-KO MD: *P*_adj_ = 0.0496. Source data

**Extended Data Fig. 19:**
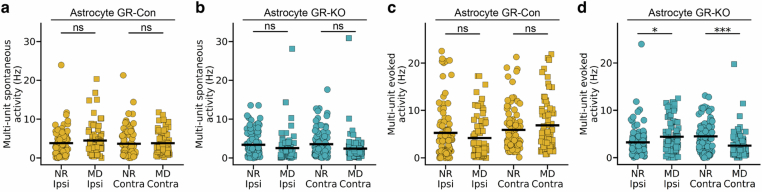
Effects of astrocyte GR signalling on spontaneous and evoked neuronal activity from individual eyes. **a-b**, Multi-unit spontaneous activity (Hz) of contralateral- or ipsilateral-responsive V1b units in adult Astro-ΔCre AAV *GR*^*fl/fl*^ (GR-Con, **a**) or Astro-Cre AAV *GR*^*fl/fl*^ (GR-KO, **b**) animals following NR or 3–4 days of MD. One-way ANOVA test with Tukey’s multiple comparisons correction; ns, not significant. Number of units: GR-Con NR Ipsi *n* = 88, GR-Con MD Ipsi *n* = 74, GR-Con NR Contra *n* = 88, GR-Con MD Contra *n* = 74, GR-KO NR Ipsi *n* = 96, GR-KO MD Ipsi *n* = 81, GR-KO NR Contra *n* = 96, GR-KO MD Contra *n* = 81. **c-d**, Multi-unit evoked activity (Hz) of contralateral- or ipsilateral-responsive V1b units in adult Astro-ΔCre AAV *GR*^*fl/fl*^ (GR-Con, **c**) or Astro-Cre AAV *GR*^*fl/fl*^ (GR-KO, **d**) animals following NR or 3–4 days of MD. One-way ANOVA test with Tukey’s multiple comparisons correction; ns, not significant; *P_adj_ < 0.05; ***P_adj_ < 0.001; multi-unit evoked GR-KO NR ipsi vs. GR-KO MD ipsi *P*_adj_ = 0.0486; multi-unit evoked GR-KO NR contra vs. GR-KO MD contra *P*_adj_ = 0.0002. Number of units: GR-Con NR Ipsi *n* = 88, GR-Con MD Ipsi *n* = 74, GR-Con NR Contra *n* = 88, GR-Con MD Contra *n* = 74, GR-KO NR Ipsi *n* = 96, GR-KO MD Ipsi *n* = 81, GR-KO NR Contra *n* = 96, GR-KO MD Contra *n* = 81.

It has previously been proposed that mature astrocytes close critical periods of heightened plasticity that occur early in postnatal development^11,12,53^. Depriving one eye of visual input for 3–4 days in early life increases the relative strength of responses from the non-deprived eye^1^, a process known as ODP. This same extent of deprivation does not elicit ODP in adult animals^54^. Given our finding that GR signalling promotes astrocyte maturation and the formation and maintenance of PNNs, we examined whether eliminating GR in V1 astrocytes of adult mice induces ODP. We delivered astrocyte-selective AAVs expressing Cre or ΔCre into V1 of P30-P40 *GR*^*fl/fl*^ mice. After allowing sufficient time for viral transduction, we recorded visual responses from the binocular zone of V1 (V1b) using multi-electrode arrays following NR or 3–4 days of monocular deprivation (MD) (Fig. 5a,b). Loss of GR in astrocytes enabled plasticity following MD (Fig. 5c,d), indicated by both increased responsiveness to the open eye and dampened responsiveness to the deprived eye (Extended Data Fig. 19a–d), without affecting visual responses under NR (Fig. 5c,d and Extended Data Fig. 19a–d). Together, these results demonstrate that eliminating astrocyte GR signalling in mature visual circuits is sufficient to re-engage ODP following short-term MD.

## Discussion

Neuronal circuits adapt to experience during critical periods in early life, thus facilitating learning, memory and behaviour. Although mature astrocytes have been shown to contribute to the closure of these critical periods across species^11,12,53^, the specific factors that drive astrocyte maturation to limit neuronal plasticity remain unclear. Here we identify GR as a master regulator of astrocyte maturation and developmental plasticity. We show that upon eye opening, which corresponds with the end of the canonical stress hormone hyporesponsive period in rodents^55^, GR binds chromatin predominantly in astrocytes, inducing an extensive program of gene expression and thereby triggering morphological changes that define a mature astrocyte. Selective disruption of GR in astrocytes induces a neotenic state characterized by diminished PNNs on neighbouring PV neurons, reactivation of the expression of axonal plasticity genes, and reopening of experience-dependent plasticity in mature animals. These findings support a broader model in which the adult brain retains a latent capacity for juvenile plasticity that may be reinstated upon removal of structural brakes enabled by astrocytic GR signalling^45^. Together with our finding of astrocyte GR-dependent gene regulation in other developing brain regions, our results raise the possibility that astrocyte GR signalling promotes neuronal circuit maturation throughout the brain.

Although neuron–astrocyte communication is a major line of research^31^, our results suggest that astrocyte reception of blood-borne factors also serves an essential role in brain development^25^. Light exposure is likely to alter the levels of other circulating hormones^56^, which may, in turn, act on astrocytes to influence brain function. Further work will be needed to understand how astrocytes integrate peripheral and local neuronal-derived signals to modulate neuronal networks and other glial populations. We note that although astrocytes largely lack gonadal hormone receptors, they express a number of other ligand-dependent nuclear receptors, including mineralocorticoid, thyroid, insulin and peroxisome proliferator-activated receptors, which may respond to changes in circulating ligands to regulate astrocyte signalling to other cell types in the brain.

Our observation that GR genomic recruitment occurs predominantly in astrocytes despite the expression of GR within most cortical cell types warrants revisiting long-standing assumptions on the genomic function of nuclear receptors in the brain. In this regard, we speculate that astrocytes may be the primary target for a number of peripheral hormones, and through ensheathing blood vessels, may buffer other cell types from exposure until the circulatory levels of such hormones reach a critical threshold. To that end, the approaches used here may be useful for characterizing astrocyte GR-driven effects on neuronal circuitry under a variety of internal states defined by heightened glucocorticoid levels, including fasting, sleep disturbance and stress.

Our finding that astrocyte GR signalling promotes cortical maturation raises the possibility that early-life stress, which increases CORT production^55,57^ and conveys enduring risk for neuropsychiatric diseases^58^, may disrupt critical periods of plasticity. In this regard, it will be important to examine whether early-life stress alters the closure of ODP by engaging astrocyte GR and, consequently, whether targeted genetic or pharmacological inhibition of this pathway occludes these effects. In support of this possibility, recent work has shown that early-life stress acts via astrocyte GR signalling to disrupt neuronal activity in the lateral hypothalamus^59^. Given that features of this pathway are conserved across human brain development, we anticipate that the outcomes of this research may be of therapeutic value, in addition to furthering our understanding of brain plasticity.

## Methods

### Animals

Animal care and surgical procedures were approved and overseen by the Harvard University Institutional Animal Care and Use Committee and the Harvard Center for Comparative Medicine or the Institutional Animal Care and Use Committee at Boston Children’s Hospital. The following mouse lines were used: C57BL/6 (Jackson Laboratory, 000664), *GR*^*fl/fl*^ (Jackson Laboratory, 021021), *Vglut1*^*cre*^ (Jackson Laboratory, 023527), *Vgat*^*cre*^ (Jackson Laboratory, 028862), *Olig2*^*cre*^ (Jackson Laboratory, 025567), *Sun1-GFP*^*fl/fl*^ (Jackson Laboratory, 021039), and *Pvalb*^*FlpO*^ (Jackson Laboratory, 022730). Mice were maintained in a reverse 12 h light:12 h dark cycle (22:00–10:00) in a temperature- and humidity-controlled environment and provided food and water ad libitum. For DR conditions, mice were maintained in chronic darkness until collection. To control for circadian rhythm and to prevent light stimulation of DR mice, mice were collected under red light between 10:00–12:00 (Zeitgeiber time 12–14 h) for all genomic, imaging, ELISA and LC–MS experiments. Male and female mice were randomly assigned to experimental groups and used in similar proportions. For the developmental genomic experiments, mice were collected between P7 and P35. Details of animal age and sex are indicated in each protocol section. For imaging experiments, mice were collected at P21, P28, P35 and 8–12-weeks of age (adult). For ELISA and LC–MS experiments, serum samples were collected at P10, P14, P21 and P28. For electrophysiology experiments, mice were patched between P33 and P35. For CORT treatment experiments, DR C57BL/6 mice received either 10 mg kg^−1^ CORT or corn oil (vehicle) at P14 via intraperitoneal injection under red light.

### Cell lines

NIH/3T3 cells (ATCC, CRL-1658) and HEK293T cells (ATCC, CRL-3216) were maintained in standard DMEM supplemented with 10% CCS, sodium pyruvate, MEM non-essential amino acids, and penicillin/streptomycin. Before GR CUT&RUN, 3T3 cells were grown in phenol-red-free DMEM containing 10% charcoal-stripped FBS for 48 h and then treated with 200 nM dexamethasone or vehicle (0.01% ethanol) for 2 h. Before Flag immunoprecipitations, HEK293T cells were grown in phenol-red-free DMEM containing 10% charcoal-stripped FBS for 24 h and then treated with 200 nM dexamethasone for 2 h.

### Serum collection

Mice were anaesthetized with 10 mg ml^−1^ ketamine and 1 mg ml^−1^ xylazine in PBS by intraperitoneal injection for terminal blood draws. Blood was collected from the left atrium of the heart with an insulin syringe. To isolate serum, blood was incubated for 30 min at room temperature and then centrifuged at 1,300*g* for 10 min at 4 °C. The supernatant (serum) was isolated and stored at −80 °C prior to corticosterone ELISA and LC–MS.

### Neonatal stereotactic surgery

P0–P1 wild-type, *GR*^*fl/fl*^ or *PV*^*Flp/Flp*^*;GR*^*fl/fl*^ mice were anaesthetized on ice and positioned within a neonatal adapter (Stoelting 51625) on a stereotaxic frame (Kopf) in which the temperature of the mouse was maintained around 4 °C using an ethanol bath containing dry ice. A small patch of skin above the V1 injection site (medial–lateral: ±1.8 mm; anterior–posterior: +0.5 mm from lambda; dorsal–ventral: −0.1 mm) was cut, and a small opening into the skull was drilled with a 30G needle. AAV (500 nl) was injected at approximately 200 nl/min, and the pipette was left in place for 5 min after infusion to allow for viral spreading. For genomic and electrophysiology assays required broad infection, mice were injected bilaterally, whereas for histology experiments, mice were injected unilaterally. Following injection, pups were recovered at 37 °C before being returned to their home cage.

### Adult stereotactic surgery

For the adult PNN staining and ODP experiments, *GR*^*fl/fl*^ mice were anaesthetized by isoflurane inhalation (3–5% induction, 1% maintenance) and positioned within a stereotaxic frame (Kopf). Animal temperature was maintained at 37 °C with a heat pad. Fur around the scalp area was removed with a shaver and sterilized with three alternating washes with betadine and 70% ethanol. A burr hole was drilled through the skull above V1 (medial–lateral: ±2.5 mm; anterior–posterior: −3.5 mm from bregma; dorsal–ventral: −0.5 mm and −0.25 mm). To broadly target superficial and deep cortical layers, 500 nl AAV was injected with a glass pipette at a depth of −0.5 mm (~200 nl/min), and an additional 500 nl was injected at a depth of −0.25 mm. After each injection, the pipette was left in place for 5 min to allow for viral spreading. All mice received postoperative analgesic (buprenorphine slow-release formulation, 1 mg kg^−1^).

### Viral vectors and titre

All AAVs used were prepared in the Boston Children’s Hospital Viral Core or ordered directly from Addgene. For genomic, postnatal and adult immunofluorescent staining, mIPSC/mEPSC recordings, and ODP experiments, viruses were densely injected into mice at 5 × 10^9^ genome copies (gc) per V1 hemisphere. For the astrocyte morphology reconstruction experiment, 2.5 × 10^9^ gc of eGFP-KASH virus was co-injected with 2.5 × 10^9^ gc of lck-smV5 virus per V1 hemisphere. For the PV-evoked inhibitory postsynaptic current electrophysiology experiment, 2.5 × 10^9^ gc of eGFP-CAAX virus was co-injected with 1 × 10^9^ gc of ChrimsonR-tdTomato virus per V1 hemisphere. The viral vectors and original titres are as follows: AAV2/5-GfaABC1D-ΔCre-T2A-eGFP-KASH-4x6T (this paper, 1.27 × 10^14^ gc ml^−1^), AAV2/5-GfaABC1D-Cre-T2A-eGFP-KASH-4x6T (this paper, 1.24 × 10^14^ gc ml^−1^), AAV2/5-GfaABC1D-ΔCre-T2A-eGFP-CAAX-4x6T-WPRE (this paper, 4.37 × 10^13^ gc ml^−1^), AAV2/5-GfaABC1D-Cre-T2A-eGFP-CAAX-4x6T-WPRE (this paper, 6.15 × 10^13^ gc ml^−1^), AAV2/5-GfaABC1D-lck-smV5-4x6T-WPRE (Addgene 196416, 9.26 × 10^13^ gc ml^−1^), and AAV2/8-CAG-FLPX-rc [ChrimsonR-tdTomato] (Addgene 130909, 2.3 × 10^13^ gc ml^−1^).

### SHARE-seq

SHARE-seq was performed on V1 tissue collected from P7, P14, P21, P28 and P35 C57BL/6 mice raised in DR or NR conditions (*n* = 4 mice per condition and time point). Mice were deeply anaesthetized with isoflurane, and V1 was dissected. The tissue was dounce homogenized in 1.5 ml of cold homogenization buffer (250 mM sucrose, 25 mM KCl, 5 mM MgCl_2_, 20 mM tricine-KOH pH 7.8, 1× EDTA-free protease inhibitor cocktail (PIC) tablet (Sigma Aldrich 11873580001), 1 mM dithiothreitol (DTT), 0.15 mM spermine, 0.5 mM spermidine, 0.1 U μl^−1^ Enzymatics RNase inhibitor (Y9240L), 0.05 U μl^−1^ SUPERase inhibitor (AM2696)) with a tight pestle for 20 strokes. The sample was then supplemented with 0.3% IGEPAL CA-630 and further dounced 5 strokes with a tight pestle. Homogenate was filtered through a 40-μm strainer into a 15 ml conical tube, and 3.5 ml of homogenization buffer and 5 ml of 50% OptiPrep solution (50% OptiPrep (Sigma D1556), 25 mM KCl, 5 mM MgCl_2_, 20 mM Tricine-KOH pH 7.8, 1× PIC, 1 mM DTT, 0.15 mM spermine, 0.5 mM spermidine, 0.1 U μl^−1^ Enzymatics RNase inhibitor, 0.05 U μl^−1^ SUPERase inhibitor) were added. Tissue lysate was underlaid with 1 ml of 30% OptiPrep solution and 1 ml of 40% OptiPrep solutions. Samples were then centrifuged at 10,000*g* for 18 min at 4 °C in an ultracentrifuge. Following centrifugation, approximately 250 μl of nuclei were collected at the 30%/40% OptiPrep interface and diluted in NIB-RI buffer (10 mM Tris pH 7.5, 10 mM NaCl, 3 mM MgCl_2_, 0.1% IGEPAL CA-630, 0.1 U μl^−1^ Enzymatics RNase inhibitor, 0.05 U μl^−1^ SUPERase inhibitor). The nuclei were then centrifuged at 500*g* for 10 min at 4 °C in a swinging-bucket centrifuge and subsequently resuspended in NIB-RI buffer containing 0.2% formaldehyde. Samples were incubated at room temperature for 5 min and then quenched on ice for 5 min with the addition of 125 mM glycine, 40 mM Tris pH 8, and 0.08% bovine serum albumin (BSA). Nuclei were then washed two times in NIB-RI buffer by centrifuging at 500*g* for 10 min at 4 °C and replacing the supernatant without disturbing the pellet. After the second wash, the supernatant was removed. Nuclei were flash-frozen in liquid nitrogen and then stored at −80 °C until all samples were collected.

SHARE-seq libraries were prepared following the original protocol^17,60^ on two batches of samples, with each batch consisting of 1 female and 1 male for each time point and condition (20 samples × 2 batches). In brief, approximately 25,000 starting nuclei were used as input for each sample. Nuclei were thawed and resuspended in NIB-RI prior to performing transposition with 6.25 μl of homemade Tn5 at 37 °C for 30 min. After one wash with NIB-RI, samples were then reverse transcribed with 25 U μl^−1^ Maxima H Minus Reverse Transcriptase. After reverse transcription, nuclei were distributed into two 96-well plates, with approximately 2,000–3,000 nuclei per well (9 wells per sample, 10 samples per plate), for 3 consecutive rounds of split-pool barcoding involving simultaneous hybridization of oligonucleotides to tagmented gDNA and cDNA, as previously described^17,60^. After the final barcoding round, the nuclei across all wells within each plate were pooled, washed two times with 1 ml of NIB-RI buffer, and resuspended in 80 μl of NIB-RI. Next, 320 μl of Ligation mix (1.25× T4 ligation buffer, 0.125% IGEPAL CA-630, 25 U μl^−1^ T4 ligase, 0.4 U μl^−1^ Enzymatics RNase inhibitor, 0.0625 U μl^−1^ SUPERase inhibitor) was added, and the samples were shaken at 300 rpm for 30 min at room temperature. Nuclei were diluted with 1 ml of NIB-RI and filtered through a 40-μm strainer into a new 1.5 ml tube. After a final centrifugation step, nuclei were resuspended in 100 μl NIB-RI and counted with a haemocytometer. Individual sub-libraries consisting of 3,000–10,000 nuclei per plate were prepared, and the samples were brought to a total volume of 50 μl with NIB-RI. Crosslinks were reversed with the addition of 50 μl of 2× RCB (100 mM Tris pH 8.0, 100 mM NaCl, 0.04% SDS), 2 μl of 20 mg ml^−1^ proteinase K, and 1 μl of SUPERase RI, followed by incubation at 55 °C for 1 h. At this stage, nuclear lysates were stored at −80 °C for several days before proceeding with the final ATAC and cDNA library preparation.

To each thawed lysate, 5 μl of 100 mM PMSF (Sigma P7626) was added, and samples were incubated at room temperature for 10 min. Pre-washed MyOne Streptavidin C1 Dynabeads (10 μl) were added to each sample, followed by 60 min incubation at room temperature on a rotator, to purify the biotinylated cDNA. ATAC and cDNA libraries were prepared from the supernatant and bead-bound fractions, as described previously^17,60^. In brief, ATAC libraries were prepared with NEBNext High-Fidelity 2× PCR Master Mix (NEB M0541L). After an initial five cycles of amplification, a 5 μl aliquot of each library was amplified in a separate qPCR reaction to determine the optimal number of PCR cycles to add (one-third of maximal SYBRgreen signal). After amplifying for additional cycles (7–8 added cycles per library), libraries were size-selected (0.5×–1.8×) with AMPure XP beads (Beckman Coulter A63880). cDNA libraries were prepared in 4 steps: template switching reverse transcription, cDNA amplification (5 PCR cycles), cDNA tagmentation, and final library amplification (7 PCR cycles). Amplified cDNA libraries were purified by 0.7× AMPure XP beads. SHARE-seq ATAC and cDNA libraries were pooled and sequenced to a depth of approximately 40,000–60,000 reads per cell on an Illumina NovaSeq 6000 using a 200-cycle kit with read configuration 50 bp × 50 bp × 99 bp × 8 bp (read 1 × read 2 × index 1 × index 2).

### MERFISH

To collect brains at P21 and P35 for MERFISH, C57BL/6 mice were deeply anaesthetized with isoflurane. Brains were collected and frozen in Optimal Cutting Temperature (OCT) compound using a dry ice ethanol bath and stored at −80 °C until sectioning. A single coronal section containing posterior V1 was collected for each sample at 10 μm thickness onto MERSCOPE slides (Vizgen 10500001). To minimize batch effects, 4 samples at each time point (1 NR female, 1 NR male, 1 DR female, 1 DR male) were mounted on the same slide. This was accomplished by micro-dissecting one brain hemisphere containing V1 per sample with fine forceps after mounting. Slides were then processed according to the MERSCOPE protocol (Vizgen 10400012), following manufacturer’s instructions. In brief, slides with tissue sections were fixed with 4% paraformaldehyde (PFA), washed 3 times in PBS, and then stored in 70% ethanol at 4 °C for 5–7 days to permeabilize the tissue. Ethanol was aspirated, and slides were washed once in Sample Prep Wash Buffer before incubating with Formamide Wash Buffer at 37 °C for 30 min. Slides were then incubated with the custom 500-Gene Panel Mix (Vizgen 10400006) for 48 h at 37 °C. Following probe hybridization, samples were washed with Formamide Wash Buffer for 30 min at 47 °C two times. After a brief wash in Sample Prep Wash Buffer, samples were embedded in a thin layer of polyacrylamide gel (Gel Embedding Solution) for 90 min at room temperature. Subsequently, slides were incubated in pre-warmed Clearing Premix solution for 24–48 h at 37 °C to clear the tissue, followed by 2 washes in Sample Prep Wash Buffer. DAPI and PolyT Staining Reagent was added, and samples were incubated for 15 min at room temperature on a rocker. Slides were washed once with Formamide Wash Buffer and once with Sample Prep Buffer before being loaded into the MERSCOPE instrument for imaging.

### Isolation of nuclei from mouse brain

The following protocol was used to isolate nuclei from early postnatal mice (P14–P28) prior to downstream CUT&RUN, ATAC-seq, bulk RNA-seq and snRNA-seq experiments. Mice were deeply anaesthetized with isoflurane. Sections of 1 mm spanning V1 were collected in an adult mouse brain matrix (Alto) on ice, and V1 was microdissected. For bulk RNA-seq and snRNA-seq experiments, tissue samples were immediately flash-frozen in a dry ice ethanol bath and stored at −80 °C prior to nuclei extraction, whereas tissue samples were processed the day of collection for CUT&RUN and ATAC-seq. After thawing or dissecting samples, V1 tissue was transferred to a 1.5 ml tube containing 350 μl of cold homogenization buffer (250 mM sucrose, 25 mM KCl, 5 mM MgCl_2_, 20 mM tricine-KOH pH 7.8 supplemented with 1× PIC, 1 mM DTT, 0.15 mM spermine and 0.5 mM spermidine). For RNA experiments, 0.3 U μl^−1^ (bulk RNA-seq) or 1 U μl^−1^ (snRNA-seq) of Protector RNase inhibitor (Sigma) was added. The tissue was dounced in a 2 ml glass homogenizer (Sigma D8938) with a loose pestle for 20 strokes. Samples were supplemented with 0.3% IGEPAL CA-630 and further dounced for an additional 10 strokes with a tight pestle. Homogenate was filtered through a small 40-μm strainer (pluriSelect 43-10040-50) into a 1.5 ml tube before diluting 1:1 with homogenization buffer and adding DRAQ5 (Abcam ab108410) at a 1:500 dilution. For CUT&RUN experiments, samples were additionally supplemented with 4 mM EDTA. Nuclei were then sorted by GFP and/or DRAQ5 signal using the Sony SH800S Cell Sorter (purity mode) with a 100-μm sorting chip, and gating was performed in the Sony SH800S Cell Sorter Software. For CUT&RUN, 175,000 DRAQ5^+^GFP^+^ or DRAQ5^+^GFP^−^ events were collected into 1 ml of cold CUT&RUN Wash Buffer (20 mM HEPES pH 7.5, 150 mM NaCl, 0.2% Tween-20, 0.5 mM spermidine, 0.1% BSA, 1× PIC). For ATAC-seq, 50,000 DRAQ5^+^GFP^+^ events were collected into 1 ml of cold ATAC-RSB (10 mM Tris-HCl pH 7.5, 10 mM NaCl, 3 mM MgCl_2_). For bulk RNA-seq, 80,000 DRAQ5^+^GFP^+^ events were collected into 350 μl of cold RLT buffer (Qiagen 74004) supplemented 1:100 with β-mercaptoethanol. For snRNA-seq, 50,000 DRAQ5^+^GFP^−^ events were collected into a 1.5 ml tube (precoated with 30% BSA) containing 300 μl of cold ATAC-RSB supplemented with 1 mM DTT, 1 U μl^−1^ Protector RNase inhibitor, and 1% BSA (Sigma A8577-10mL).

### CUT&RUN

To perform CUT&RUN^61^ on 3T3 cells, cells were washed twice with cold PBS and then scraped in NE1 lysis buffer (20 mM HEPES pH 7.9, 10 mM KCl, 0.1% Triton X-100, 3 mM MgCl_2_, 0.5 mM spermidine, 1× PIC, 10 mM sodium butyrate). Nuclei were rotated for 10 min at 4 °C and centrifuged at 500*g* for 10 min at 4 °C. The nuclei pellet was resuspended in 1 ml of CUT&RUN Wash Buffer. 50,000 or 250,000 nuclei were then bound to 20 μl of concanavalin A beads (Bangs Laboratories, BP531) for 30 min at 4 °C on a rotator. Samples were then incubated for 16–20 h on a tube nutator with primary antibody (GR: Invitrogen MA1-510 and PA1-511A, IgG: Cell Signaling Technology 2729S) diluted 1:100 in Antibody Buffer (CUT&RUN Wash Buffer containing 2 mM EDTA and 0.1% Triton X-100). After primary antibody incubations, samples were washed twice in Antibody Buffer and then resuspended in Triton wash buffer (CUT&RUN Wash Buffer containing 0.1% Triton X-100). Protein A-MNase (pA-MNase, prepared in-house) was added at a concentration of 700 ng ml^−1^, and samples were incubated at 4 °C for 1 h on a tube nutator. Two washes in Triton wash buffer were performed, after which the tubes were incubated in a metal block on ice for 5 min. pA-MNase digestion was initiated with the rapid addition of 2 mM CaCl_2_. After 60 min, pA-MNase digestion was halted by adding 2× stop buffer (340 mM NaCl, 20 mM EDTA, 4 mM EGTA, 0.04% Triton X-100, 50 μg ml^−1^ RNase A, 50 μg ml^−1^ glycogen, 100 pg yeast spike-in DNA) at a 1:1 dilution. Digested fragments were then released at 37 °C for 10 min, followed by centrifugation at 16,000*g* for 5 min at 4 °C. Released DNA was treated with 0.1% SDS and 0.13 mg ml^−1^ proteinase K at 50 °C for 1 h with gentle shaking. DNA was then purified by phenol-chloroform extraction, using an established protocol^61^. CUT&RUN libraries were prepared with the SMARTer ThruPLEX DNA-seq Kit (Takara Bio R400676), with the following PCR conditions: 72 °C for 3 min, 85 °C for 2 min, 98 °C for 2 min, (98 °C for 20 s, 67 °C for 20 s, 72 °C for 30 s) × 4 cycles, (98 °C for 20 s, 72 °C for 15 s) × 8 cycles. All samples were purified with AMPure XP beads (0.5×–1.7×) and sequenced paired-end on an Illumina NextSeq 500 with a 75-cycle kit.

For astrocyte GR and IgG CUT&RUN experiments on DR and NR mice, each biological replicate consisted of P14 V1 tissue pooled from 3-5 C57BL/6 mice that were injected at P0–P1 with AAV2/5-GfaABC1D-ΔCre-T2A-eGFP-KASH-4x6T. For astrocyte GR and NFIA CUT&RUN experiments on GR-con and GR-KO mice, each biological replicate consisted of P14 V1 tissue pooled from 3-5 *GR*^*fl/fl*^ mice injected at P0–P1 with AAV2/5-GfaABC1D-ΔCre-T2A-eGFP-KASH-4x6T (GR-con) or AAV2/5-GfaABC1D-Cre-T2A-eGFP-KASH-4x6T (GR-KO). For GR, NFIA, and IgG CUT&RUN experiments on excitatory neurons, inhibitory neurons, or oligodendrocytes, each biological replicate consisted of P14 V1 tissue pooled from 3–5 *Vglut1*^*cre/+*^*;Sun1-GFP*^*fl/+*^, *Vgat*^*cre/+*^*;Sun1-GFP*^*fl/+*^, *Olig2*^*cre/+*^*;Sun1-GFP*^*fl/+*^ mice, respectively. NFIA CUT&RUN was performed with Invitrogen PA5-52252 diluted 1:100 in Antibody Buffer. Following fluorescence-activated nuclei sorting (FANS) collection of nuclei into CUT&RUN Wash Buffer, nuclei were bound to concanavalin A beads, and all downstream CUT&RUN steps were performed, as described above.

### ATAC-seq

Each biological replicate consisted of P21 V1 tissue pooled from 3-4 *GR*^*fl/fl*^ mice that were injected at P0–P1 with AAV2/5-GfaABC1D-ΔCre-T2A-eGFP-KASH-4x6T (GR-con) or AAV2/5-GfaABC1D-Cre-T2A-eGFP-KASH-4x6T (GR-KO). After sorting nuclei into ATAC-RSB, 0.1% Tween-20 was added, and the nuclei were centrifuged at 500*g* for 10 min at 4 °C. The nuclei pellet was directly resuspended in the transposition reaction mix, according to the OMNI-ATAC protocol^62^. A 1 μl volume of homemade Tn5 enzyme was used in the transposition reaction. Libraries were prepared with NEBNext High-Fidelity 2× PCR Master Mix (NEB M0541L), following the standard protocol. After an initial five cycles of amplification, another four cycles were added to achieve optimal library concentration, based on qPCR quantification. Libraries were then size-selected (0.5×–1.8×) twice with AMPure XP beads (Beckman Coulter A63880) and sequenced paired-end on an Illumina NextSeq 500 using a 75-cycle kit.

### Bulk RNA-seq

Each biological replicate consisted of flash-frozen V1 tissue pooled from 1 male and 1 female *GR*^*fl/fl*^ mice that were injected at P0–P1 with AAV2/5-GfaABC1D-ΔCre-T2A-eGFP-KASH-4x6T (GR-con) or AAV2/5-GfaABC1D-Cre-T2A-eGFP-KASH-4x6T (GR-KO). After sorting nuclei into RLT buffer, RNA was extracted using the RNeasy Micro Kit (Qiagen 74004). RNA-seq libraries were prepared from 10 ng RNA per sample using the SMARTer Stranded Total RNA-Seq Kit v2 (Takara 634413), according to the manufacturer’s instructions. Libraries were sequenced paired-end on an Illumina NextSeq 500 using a 75-cycle kit.

### snRNA-seq

snRNA-seq was performed on V1 tissue collected from P21 *GR*^*fl/fl*^ mice injected at birth with AAV2/5-GfaABC1D-ΔCre-T2A-eGFP-CAAX-4x6T-WPRE (GR-con) or AAV2/5-GfaABC1D-Cre-T2A-eGFP-CAAX-4x6T-WPRE (GR-KO) and then reared in DR or NR conditions. V1 was flash-frozen and stored at −80 °C until *n* = 2 mice were collected per group, as described above. Nuclei were extracted and sorted into cold ATAC-RSB, as described above. After collection, nuclei were centrifuged two times at 200*g* for 2 min in a swinging-bucket centrifuge at 4 °C. The supernatant was then carefully aspirated from the surface of the liquid until approximately 40 μl remained. Nuclei were gently resuspended in the residual volume and immediately processed with the 10X Genomics Single Cell 3’ Gene Expression Kit v4 (PN-1000686), following the manufacturer’s instructions. The 8 libraries were pooled and sequenced on an Illumina NovaSeq X to a depth of approximately 40,000–90,000 reads per cell.

### Corticosterone ELISA

Total corticosterone concentration in 5 μl of mouse serum was measured with a commercial corticosterone ELISA kit (Arbor Assays K014-H1), following manufacturer’s instructions.

### LC–MS

Mouse serum samples were processed for LC–MS detection of aldosterone, corticosterone, cortisol, and thyroxine (T4) by the Harvard Center for Mass Spectrometry. In brief, 20 μl of mouse serum or hormone standards (a 15 point, 1/3 dilution series with 1 μM for the highest concentration of aldosterone, cortisol, corticosterone, and T4) were mixed with 100 μl of extraction solution (methanol containing internal standards cortisol-D4, corticosterone-D8, and T4-13C6 each at 0.15 nM, Cayman Chemicals). Samples were centrifuged at 18,000*g* at −11 °C to remove proteins. The supernatant was transferred to silanized glass microinserts and evaporated to dryness under nitrogen flow. Samples were then resuspended in 15 μl of 50% methanol solution in water. Samples (5 μl) were run on a Kinetex C18 column (150 × 2.1 mm, Phenomenex) at a column temperature of 35 °C. Mobile phase A was water with 0.1% formic acid, and mobile phase B was acetonitrile with 0.1% formic acid. The gradient was as follows: 5 min at 20% B, then 10 min at 100% B, followed by 5 min at 100% B. The column was then re-equilibrated to 20% B for 5 min. The data were acquired in positive mode on a Sciex 7500 Triple Quad Mass Spectrometer (AB Sciex). The source parameters were: gas 1: 60 psi, gas 2: 70 psi, curtain gas: 40 psi, CAD gas: 8, source temperature: 550 °C, capillary at 4,000 V. The transitions used are shown in Supplementary Table 5. Mouse serum hormone concentrations were calculated using the data from the standard curve.

### Immunoprecipitation of Flag-tagged GR and NFIA in HEK293T cells

Plasmids (pCIG with CAG promoter) containing the sequence for Flag-tagged GR or HA-tagged NLS–GFP, LHX2, or NFIA were co-delivered into HEK293T cells by CaCl_2_-BBS transfection (10 μg of each plasmid, 10 × 10^6^ cells). The same approach was used to co-deliver Flag-tagged NFIA with HA-tagged NLS–GFP or LHX2. After 24 h, the media was replaced with phenol-red-free DMEM containing charcoal-stripped FBS, as described above, and incubated for another 24 h. Cells were then treated with 200 nM dexamethasone for 2 h. Cells were washed twice with ice-cold PBS and then scraped in Nuclear Extraction (NE) Buffer (20 mM HEPES pH 7.9, 10 mM KCl, 3 mM MgCl_2_, 0.1% Triton X-100, 1× PIC, phosphatase inhibitors 2 and 3). Cells were pelleted by gentle centrifugation (500*g* for 10 min at 4 °C). Flag–GR samples were extracted with either benzonase (Sigma Aldrich E8263) or MNase (NEB M0247S), while Flag–NFIA samples were extracted with benzonase. For benzonase extraction, samples were resuspended in 1 ml of NE Buffer, 5 μl of benzonase was added, and samples were incubated for 30 min at 4 °C with rotation. NaCl was added to a concentration of 300 mM, and samples were mixed for an additional 30 min at 4 °C with rotation. Lysates were spun at 16,000*g* for 20 min at 4 °C, and the supernatant was transferred to a fresh tube. Samples were then diluted to 200 mM NaCl in NE Buffer. For MNase extraction, samples were resuspended in NE Buffer, and 1 mM CaCl2 and 24 μl of MNase were added. Samples were incubated at 37 °C for 20 min and then MNase stop buffer (40 mM EGTA, 20 mM EDTA, 3 M NaCl) was added at a 1:20 dilution. Lysates were spun at 16,000*g* for 20 min at 4 °C, and the supernatant was transferred to a fresh tube. For each sample, 5% of lysate was collected for the input fraction. Samples were pre-cleared with 50 μl of washed agarose resin (Sigma Aldrich A0919). Immunoprecipitations were then performed using 100 μl of anti-Flag magnetic agarose resin (Thermo Fisher A36798) for 2 h at 4 °C with rotation. Following immunoprecipitations, samples were washed 4 times (5 min at 4 °C for each wash) with NE Buffer containing 250 mM NaCl. After the final wash, samples were eluted with 3× Flag peptide (Sigma Aldrich F4799) diluted to 0.5 mg ml^−1^ in NE Buffer containing 250 mM NaCl for 30 min at RT at 1,000 rpm and then stored at −80 °C until immunoblotting.

### Immunoblotting

Input or immunoprecipitation fractions were resolved on NuPage 4–12% Bis-Tris gels (Thermo Fisher, NP0336BOX) and transferred to nitrocellulose membranes. Membranes were blocked for 1 h with SuperBlock (Thermo Fisher 37518) and incubated overnight with the following primary antibodies (1:1,000): Flag (Sigma Aldrich F1804) and HA (Cell Signaling Technology 3724). Following washing, membranes were incubated with secondary antibodies against rabbit IgG or mouse IgG conjugated to horseradish peroxidase (Cell Signaling Technology 7074S or 7076S, respectively) at a concentration of 1:5,000, labelled with SuperSignal Pico PLUS reagents (Thermo Fisher A45915), and imaged using a Bio-Rad Gel Doc System. Full scans of western blots are shown in Supplementary Figs. 1 and 2.

### Immunofluorescent staining

Male and female mice were used in equal proportions for histology experiments. Mice were anaesthetized by intraperitoneal injection of 10 mg ml^−1^ ketamine and 1 mg ml^−1^ xylazine. Upon anaesthetization, mice were transcardially perfused with 15–20 ml of cold PBS followed by 15–20 ml of 4% PFA diluted in PBS. Brains were incubated in 4% PFA at 4 °C for 16–20 h, followed by two washes in PBS. Brains were then sectioned at 50 μm or 100 μm (for astrocyte reconstruction experiment) thickness on a vibratome and stored in PBS at 4 °C for 48–72 h prior to staining. Sections were blocked in PBS containing 0.1% Triton X-100 and 10% normal donkey serum (NDS, Sigma D9663) for 1 h at room temperature. Staining in primary antibodies (dilutions listed below) or biotinylated WFA (Vector Laboratories B-1355-2, 1:1,000) was carried out in PBS containing 0.1% Triton X-100 and 0.5% NDS (PDT buffer) at room temperature for 16–20 h with shaking. For labelling astrocyte morphology in 100 μm-thick sections, primary antibody incubation was performed at 4 °C for 48 h. After labelling with primary antibody, sections were washed 3 times (10 min each) in PDT buffer. Secondary antibody labelling was performed with fluorophore-conjugated secondary antibodies (rabbit Dylight 405 (Jackson ImmunoResearch 711-475-152), chicken Alexa Fluor 488 (Jackson ImmunoResearch 703-545-155), rabbit Alexa Fluor 594 (Life Technologies A21207), mouse Alexa Fluor 594 (Jackson ImmunoResearch 715-585-150), guinea pig Alexa Fluor 647 (Jackson ImmunoResearch 706-605-148), goat Alexa Fluor 647 (Life Technologies A21447), mouse Alexa Fluor 647 (Life Technologies A31571)) diluted 1:300 or streptavidin Alexa Fluor 647 (Jackson ImmunoResearch 016-600-084) diluted 1:1,000 in PDT buffer for 2 h at room temperature. For experiments requiring nuclear labelling, DAPI was added at 1 μg ml^−1^ during secondary antibody incubation. Sections were again washed two times (10 min each) in PDT buffer, followed by an additional two washes with PBS containing 0.1% Triton X-100. Sections were then transferred to PBS and mounted with ProLong Diamond Antifade mountant (Invitrogen P36970) and imaged at 16-bit depth on a point scanning confocal microscope (Leica Stellaris 5). Primary antibodies were diluted in PDT buffer as follows: rabbit anti-GR (Cell Signaling Technology 12041S, 1:300), goat anti-SOX9 (R&D Systems AF3075, 1:300), chicken anti-GFP (Aves Labs GFP-1020, 1:1,000), rabbit anti-Acan (Sigma AB1031, 1:500), guinea pig anti-PV (Synaptic Systems 195-308, 1:1,000), rabbit anti-PV (Swant PV27a, 1:1,000), rabbit anti-HOMER1 (Synaptic Systems 160003, 1:500), guinea pig anti-Vglut1 (Sigma AB5905, 1:500), rabbit anti-NeuN (EMD Millipore ABN78, 1:500), mouse anti-SYT2 (ZIRC ZDB-ATB-081002-25, 1:500), and guinea pig anti-CB1R (Frontier Institute MSFR100630, 1:300).

For validating the astrocyte specificity of our viruses and assessing the degree of GR protein KO, one V1 was imaged with the 63× objective at 1,024 × 1,024 pixel resolution with 0.75× optical zoom (11 *z*-planes, 3 μm step size) and quantified per animal. For reconstructing astrocyte morphology, V1 astrocytes within 2–4 fields of view (FOVs) across 2–3 sections were imaged with the 63× objective at 2,048 × 2,048 pixel resolution with 0.75× optical zoom (80–140 *z*-planes, 0.5 μm step size). For P21, P28, P35 and adult PNN and PV staining, V1 was imaged in 2 coronal sections with the 63× objective at 1,024 × 1,024 pixel resolution with 0.75× optical zoom (12 *z*-planes, 2 μm step size) and quantified at the animal level. For adult V1 L5 Acan and PV staining, 3–5 FOVs in 2–3 coronal sections were imaged with the 63× objective at 1,024 × 1,024 pixel resolution with 0.75× optical zoom (37 *z*-planes, 1 μm step size) and quantified at the animal level. For V1 L5 Vglut1/HOMER1 staining experiments, 3–5 FOVs in 2–3 coronal sections were imaged with the 100× objective at 2,048 × 2,048 pixel resolution with 2.5× optical zoom (31 *z*-planes, 0.5 μm step size) and quantified at the animal level. For V1 L5 SYT2/CB1R staining experiments, 3–5 FOVs in 2–3 coronal sections were imaged with the 100× objective at 1,024 × 1,024 pixel resolution with 0.75× optical zoom (41 *z*-planes, 0.5 μm step size) and quantified at the animal level. For P28 SYT2 staining (Extended Data Fig. 17e,f), the same parameters were used, and 51 *z*-planes were collected.

### Electrophysiology

#### Acute slice preparation

Coronal cortical slices were prepared from P33-35 *GR*^*fl/fl*^ mice injected at birth with AAV2/5-GfaABC1D-ΔCre-T2A-eGFP-CAAX-4x6T-WPRE (GR-con) or AAV2/5-GfaABC1D-Cre-T2A-eGFP-CAAX-4x6T-WPRE (GR-KO) for the mIPSC/mEPSC recordings. Slices were also prepared from P33-35 *Pvalb*^*FlpO/FlpO*^*;GR*^*fl/fl*^ mice co-injected at birth with AAV2/8-CAG-FLPX-rc (ChrimsonR-tdTomato) and AAV2/5-GfaABC1D-ΔCre-T2A-eGFP-CAAX-4x6T-WPRE (GR-con) or AAV2/5-GfaABC1D-Cre-T2A-eGFP-CAAX-4x6T-WPRE (GR-KO) to measure PV-evoked IPSCs. Mice were anaesthetized with isofluorane and transcardially perfused with ice-cold choline-based artificial cerebrospinal fluid (choline ACSF, in mM: 110 choline chloride, 25 NaHCO_3_, 1.25 NaH_2_PO_4_, 2.5 KCl, 7 MgCl_2_, 0.5 CaCl_2_, 25 glucose, 11.6 sodium-l-ascorbate and 3.1 sodium pyruvate, 320–330 mOsm) equilibrated with 95% O_2_/5% CO_2_. After perfusion, the brain was rapidly dissected and blocked in ice-cold equilibrated choline ACSF. Tissue was then transferred to a cutting chamber containing ice-cold equilibrated choline ACSF and cut on a Leica VT1200S (300 µm thickness, 0.10 mm s^−1^, 1 mm amplitude). Slices were then collected in a holding chamber containing artificial cerebrospinal fluid (ACSF) (in mM: 127 NaCl, 25 NaHCO_3_, 1.25 NaH_2_PO_4_, 2.5 KCl, 1 MgCl_2_, 2 CaCl_2_ and 10 glucose, 300–310 mOsm). Slices were recovered at 32 °C for 20 min and then maintained at room temperature (20–22 °C) for at least 20 min before the start of recordings. Experiments were performed within 6 h after cutting at room temperature.

#### Ex-vivo slice electrophysiology

For whole-cell voltage-clamp recordings of miniature excitatory and inhibitory currents, patch pipettes made with borosilicate glass with filament (Sutter BF150-86-7.5) and 2–5 MΩ resistance were filled with a Cs^+^-methanesulfonate internal solution consisting of (in mM): 127 CsMeSO3, 10 CsCl, 10 HEPES, 0.5 EGTA, 2 MgCl2, 0.16 CaCl2, 2 MgATP, 0.4 NaGTP, 14 sodium phosphocreatinine, and 2 QX314-Cl (pH 7.2, 295 mOsm). Recordings were made on an upright Olympus BX51 W1 microscope with an infrared CCD camera (Dage-MTI IR-1000) and 60× water immersion objective (Olympus Lumplan FI/IR 60 Å/0.90 NA). Neuronal tissue was visualized with infrared differential interference contrast. GFP-expressing astrocytes were identified by epifluorescence driven by a light-emitting diode (Excelitas XCite LED120). Layer 5 pyramidal neurons in close proximity to GFP signal were identified based on the depth from pia (450–590 µm) and their large somas and apical dendrites. For isolation of excitatory and inhibitory miniature currents, cells were clamped at −65mV and 0 mV, respectively in the presence of 0.5 µM TTX (Tocris 1069). For measuring PV-mediated evoked IPSCs, a CsCl-based internal solution consisting of (in mM): 135 CsCl, 3.3 QX314-Cl, 10 Hepes, 4 MgATP, 0.5 NaGTP, 8 sodium phosphocreatine, 1.1 EGTA and 0.1 CaCl_2_ (pH 7.2, 290 mOsm) was used, and cells were clamped at −70mV. NBQX (Tocris 1044, 10 µM) and (R)-CPP (Tocris 0247, 10 µM) were added to the ACSF perfusion to pharmacologically block AMPA and NMDA receptors, respectively.

#### Ex vivo slice electrophysiology data acquisition and analysis

Synaptic miniature excitatory or inhibitory currents were collected for three minutes in whole-cell voltage-clamp (36 sweeps of 5 s length). At the end of each sweep, a 200 ms long −5 pA current step was added to continuously measure series resistance, membrane resistance and capacitance. For photostimulation of ChR2-expressing boutons, orange-red light at 590 nm was delivered through the 60× objective lens using the light-emitting diode, controlled via TTL pulses synchronized with the electrophysiology acquisition system pCLAMP (10.6) Clampex module. A single light stimulation of 0.5 ms was delivered at increasing light intensities at the slice surface between 1–4 mW mm^−2^, as measured with a photodiode sensor for evoked recordings, and five pulses at 10 Hz were delivered for short-term plasticity experiments. Light intensity was chosen based on prior studies that elicited PV-evoked potentials^63,64^. Cells that needed more than −500 pA of current injection for clamping at desired mV, exceeded 30 mOhm of series resistance, or changed their series resistance by more than 30% before the start and end of the experiment were excluded. An Axon Multiclamp 700B (controlled by Multiclamp 700B commander) was used to perform voltage- and current-clamp experiments and low-pass filtering at 4 kHz. Data were sampled at 10 kHz with an Axon Digidata 1,440 A and controlled using the Clampex module of pCLAMP (10.6). Miniature events were analysed using template event detection in the Clampfit module of pCLAMP (10.6). Evoked responses were analysed using peak amplitude measurements. All measurements are displayed as mean ± s.e.m. Experiments and data analysis were performed blind to genotype. Statistical testing was performed in Prism 10 using the appropriate *t*-test based on normality distribution and equal standard deviation (unpaired *t*-test, Mann–Whitney test, Kolmogorov–Smirnov test) for mEPSC and mIPSC currents. Two-way analysis of variance (ANOVA) testing with Bonferroni correction was performed for PV-mediated evoked currents and short-term plasticity.

### Ocular dominance plasticity

#### Monocular deprivation

MD was performed while mice were under isoflurane anaesthesia (4% for induction and 2% for maintenance). The margins of the left eyelid were trimmed, and the lids were sutured closed (Ethicon 7-0 Perma-Hand Silk Sutures). Mice were monocularly deprived for 3–4 days prior to ocular dominance recordings.

#### In vivo extracellular recordings from primary visual cortex

Recordings were performed in anaesthetized mice using standard acute in vivo electrophysiological procedures^65,66^. Mice were anaesthetized with isoflurane (4% induction, 2% maintenance) and a ~1.5 mm diameter craniotomy was made centred 3 mm lateral to the lambda suture. Mice received 2.5 mg kg^−1^ dexamethasone subcutaneously to reduce oedema. Eyes were lubricated with silicone oil (Sigma) to prevent corneal drying. To stabilize anaesthesia during recordings, mice received chlorprothixene (5 mg kg^−1^, subcutaneous)^65^, and isoflurane was reduced to ~1% during data acquisition. Extracellular signals were recorded using multisite silicon probes (Cambridge Neurotech, ASSY-37-H7b). Data were acquired through a PZ5 digitizer and RZ5P processor using Synapse software (Tucker-Davis Technologies). Up to three probe penetrations were performed in each animal. Recordings were always performed in the right visual cortex—contralateral relative to the sutured eye. Electrodes were coated with DiD (Invitrogen) in the last penetration to enable post hoc visualization of the electrode track. Experimenters were blind to the AAV (Cre versus ΔCre) injected into V1.

#### Visual stimulation

Visual stimuli were presented on a 21 × 12 inch LED monitor with a refresh rate of 60 Hz and mean luminance of 20 lux. The monitor was centred on the binocular visual field, perpendicular to the antero-posterior axis of the mouse. Visual stimuli were generated using the Psychophysics toolbox^67^ in MATLAB (Mathworks). To confirm that receptive fields were in the binocular zone, contrast-modulated white noise movies were presented^65^. To measure evoked responses to visual stimuli, mice were presented with full-field drifting grating moving in 8 different directions with a spatial frequency of 0.02 cycles per degree and a temporal frequency of 2 cycles per second. A blank grey screen stimulus was presented to estimate spontaneous activity. Gratings were presented in pseudorandom order for 40 trials in total, alternating between the ipsilateral and contralateral eyes.

#### Analysis

Adequate AAV targeting of V1, as measured by viral GFP immunofluorescence, was confirmed for each animal post hoc as a criterion for inclusion in the ODP experiment. Spikes were extracted using an amplitude threshold set at 6× s.d. above baseline noise. Recording sites (multi-units) were included for analysis if they were located within the binocular zone, defined as positions within 25° in azimuth. Responses to drifting gratings were subtracted from responses to the blank stimulus to measure evoked firing. Responses to gratings moving in all directions were averaged to calculate the evoked firing rate of each contact site. Ocular dominance index was computed for each recording site as (*R*_contra_ – *R*_ipsi_)/(*R*_contra_ + *R*_ipsi_), where *R*_contra_ and *R*_ipsi_ are the evoked responses to the contralateral and ipsilateral eye, respectively. Ocular dominance index measurements are displayed per recording site (multi-unit) and averaged per animal. Statistical testing was performed in Prism 10 using the Kruskal–Wallis test with Dunn’s multiple comparisons correction or one-way ANOVA test with Tukey’s multiple comparisons correction.

### Bioinformatics and data analysis

#### Imaging data analysis

All image collection and analysis was performed by an investigator blinded to experimental conditions. For validating the astrocyte specificity of our viruses, assessing the degree of GR knockout, and quantifying the heterogeneity of GR expression in V1 astrocytes at P35 and P21 (Extended Data Fig. 8h–m), images were loaded into Fiji/ImageJ, and the proportion of GFP^+^ cells containing SOX9 (SOX9^+^GFP^+^) or GR (GR^+^GFP^+^) as well as the proportion of SOX9^+^ cells expressing GR (GR^+^SOX9^+^) were counted manually using the multi-point tool.

All other image analysis was performed in Imaris v10.2 (Oxford Instruments). To quantify the cell volume and NIV of GR-con and GR-KO astrocytes (Fig. 3a,b), the smV5 fluorescence signal was reconstructed using the surface tool. Individual astrocytes (examples shown in Extended Data Fig. 12a) were selected for quantification: (1) if the cell boundaries in 3D space could be identified; and (2) if the cell contained a GFP-labelled nuclear membrane, indicating co-transduction with AAV2/5-GfaABC1D-ΔCre-T2A-eGFP-KASH-4x6T (GR-con) or AAV2/5-GfaABC1D-Cre-T2A-eGFP-KASH-4x6T (GR-KO). NIV per astrocyte was calculated in Imaris, as previously described^36^. In brief, the astrocyte smV5 surface volume within three randomly selected 10 µm × 10 µm × 10 µm boxes (located within the neuropil) was quantified and averaged per cell.

To quantify PV neuron-bound Acan, the PV fluorescence signal was reconstructed using the surface tool. The mean Acan fluorescence intensity on PV neuron surfaces per FOV was calculated in Imaris and then averaged across all FOVs per animal (Extended Data Fig. 16n,o). To quantify PNN and PV intensity at P35 and in adults, the PV fluorescence signal was reconstructed using the surface tool, and PV neuron surface objects were manually annotated to cortical layers (L2/3, L4, L5 and L6) based on the DAPI channel. The mean WFA or PV fluorescence intensity on PV neuron surfaces for each cortical layer was then calculated in Imaris and averaged between two coronal sections per animal (Fig. 4a–d and Extended Data Fig. 16d,h,j,l).

To measure the effect of astrocyte GR knockout on SYT2^+^ or CB1R^+^ synapses on neuronal somas, the astrocyte mGFP fluorescence signal and NeuN fluorescence signal were reconstructed using the surface tool. NeuN surfaces overlapping astrocyte mGFP surfaces were then selected and duplicated into a new surface object. SYT2^+^ puncta (size = 1.3 μm) and CB1R^+^ puncta (size = 1.3 μm) were reconstructed using the spots tool. SYT2^+^ and CB1R^+^ spots within 0.9 μm of mGFP-contacting NeuN surfaces were identified using the Matlab extension ‘spots close to surface’. The density of SYT2^+^ or CB1R^+^ spots per NeuN surface volume (μm^3^) was then calculated for each NeuN surface per FOV, and the median density was computed across FOVs per animal (Fig. 4e,f and Extended Data Fig. 17a,b,e,f).

To measure the effect of astrocyte GR knockout on Vglut1^+^HOMER1^+^ synapses, the astrocyte mGFP fluorescence signal was reconstructed using the surface tool, and Vglut1^+^ (size = 0.6 μm) and HOMER1^+^ (size = 0.4 μm) puncta were reconstructed using the spots tool. Vglut1^+^ and HOMER1^+^ spots within 0.5 μm of the astrocyte mGFP surface were selected using the Matlab extension ‘spots close to surface’. Vglut1^+^ and HOMER1^+^ puncta within 0.5 μm of each other were then detected using the Matlab extension ‘co-localize spots’. The density of co-localized Vglut1^+^HOMER1^+^ spots per astrocyte mGFP surface volume (μm^3^) was then calculated per FOV, and the median density was computed across FOVs per animal (Fig. 4g,h and Extended Data Fig. 17c,d,g,h).

#### SHARE-seq data processing and analysis

SHARE-seq data were processed using the SHARE-seq-alignmentV2 pipeline (https://github.com/masai1116/SHARE-seq-alignmentV2), generating gene × nucleus matrices for the snRNA assay and fragment files for the single-nucleus ATAC assay. snRNA gene × nucleus matrices were loaded into Seurat^68^ (v5.3.0), and nuclei with fewer than 600 gene counts were removed. Scrublet^69^ was used to predict and remove doublets, and post-doublet removal, nuclei were further filtered on the basis of gene counts, retaining nuclei that have between 750 and 80,000 gene counts. Gene counts were normalized and log-transformed (NormalizeData), and the top 2,000 variable features were identified using FindVariableFeatures. Gene counts were scaled (ScaleData), regressing out the number of RNA counts, number of RNA genes, and percentage of mitochondrial gene counts per nucleus. Dimensionality reduction was performed with principal component analysis (runPCA), and a *k*-nearest-neighbours graph was constructed (FindNeighbors, npcs = 15). Nuclei were clustered to identify principal cell classes with the Louvain algorithm (FindClusters, resolution = 0.2) and visualized by UMAP (runUMAP, dims = 15). Nuclei were annotated to broad cell classes (astrocytes, endothelial cells, excitatory neurons, inhibitory neurons, microglia, oligodendrocytes, OPCs, SMC–Peri, VLMC) on the basis of known marker genes. Following this initial round of clustering, excitatory neurons (*Slc17a7*+ clusters) and inhibitory neurons (*Gad2*+ clusters) were subsetted and re-clustered to identify cell subtypes, using the same procedure described above (FindClusters, resolution = 0.3). After clustering, any nuclei cluster lacking significant marker genes (FindMarkers) were removed, resulting in the final processed dataset (70,907 cells). Single-nucleus ATAC fragment files were loaded into Signac^70^ (v1.14.0), and initial 5,000 bp × cell genome bin matrices were constructed using the GenomeBinMatrix function. Chromatin assay objects were then created using the CreateChromatinAssay function, and these objects were subsequently filtered to keep cell barcodes present in the snRNA assay. Peaks were called on the dataset using MACS2 (CallPeaks). Gene accessibility score (shown in Extended Data Fig. 1e,h,i), calculated as the ATAC signal over the gene body ± 2,000 bp, was quantified with the GeneActivity function.

Pseudobulk differential expression analysis between NR and DR conditions for each cell type and developmental time point was performed using edgeR glmQLFTest (*P*_adj_ < 0.05). Differentially expressed genes were separated into cell type-shared (differential in ≥2 cell types) or cell-type-specific (differential in 1 cell type), and shared genes were then hierarchically clustered by log2(fold change) value using the ward.D2 algorithm in pheatmap for visualization in Fig. 1d. To identify astrocyte experience-responsive genes (Fig. 2f, Extended Data Fig. 9a–c) for comparison to GR binding sites, genes differentially expressed between NR and DR (edgeR *P*_adj_ < 0.05) astrocytes at any time point were grouped and hierarchically clustered using the average algorithm in pheatmap with cutree = 4. Pseudobulk differential accessibility analysis was performed between NR and DR conditions for each cell type and developmental time point using edgeR glmQLFTest (*P*_adj_ < 0.1), after pooling early (P7–P14) and late (P21–P35) time points to increase cell depth.

To identify transcription factors influenced by visual experience (Fig. 2a), ATAC peaks were annotated for the presence of transcription factor binding motifs (JASPAR 2020 database) using the AddMotifs function. ChromVAR^20^ was then used to calculate the background-corrected, genome-wide chromatin accessibility score of all transcription factor binding motifs in individual nuclei. Experience-regulated transcription factor chromVAR scores for each cell type at P21 were then identified using MAST^71^ within the FindMarkers function. The top 2 unique differentially accessible transcription factor binding motifs (by *P*_adj_ value) between NR and DR for each cell type are shown in a heat map in Fig. 2a, in which each row is scaled to the maximal within-row −log10(*P*_adj_) value. To predict cell identity transcription factors from SHARE-seq data (Extended Data Fig. 5a), as done previously^17^, the Pearson correlation coefficient between transcription factor chromVAR score and transcription factor expression was calculated at the cell type level for all JASPAR2020 transcription factors with matched motif and gene names. Transcription factors with a correlation coefficient >0.3 and maximal transcription factor RNA FC >1 between cell types were highlighted in red in Extended Data Fig. 5a (top candidate transcription factors for each cell type shown in Extended Data Fig. 5b). To identify experience-responsive, cell type-enriched transcription factor binding motifs (Extended Data Fig. 5c–g), nuclei were subsetted to late postnatal time points (P21–P35), which display the majority of experience-responsive genes and chromatin regions. Experience-regulated transcription factor chromVAR scores and cell type-enriched transcription factor chromVAR scores were separately computed for each cell type using MAST^71^ within the FindMarkers function. To prioritize transcription factor binding motifs that are strongly influenced by experience and more accessible in specific cell types, the rank-product of these 2 statistical tests was calculated, and the top 2–3 transcription factors per cell type based on rank-product were selected to display in Extended Data Fig. 5c.

#### MERFISH data processing and analysis

MERFISH imaging data were processed on the MERSCOPE instrument with cell segmentation using a watershed algorithm based on DAPI and PolyT staining. Individual images were then loaded into MERSCOPE Visualizer and a region of interest (ROI) around V1 was drawn to subset cells for downstream analysis. STAlign^72^ was used to spatially align the centroid position of individual cells across P21 and P35 tissue sections. MERFISH expression matrix and cell boundary files were then loaded and analysed in Seurat (v5.3.0). Cells with fewer than 20 gene counts were removed. Gene counts were normalized and log-transformed and then scaled (ScaleData), regressing out the number of transcript counts per cell. Dimensionality reduction and clustering were performed, as described above for SHARE-seq. Cells were annotated to broad cell classes on the basis of known marker genes. Subsequently, excitatory neurons (*Slc17a7*+) and inhibitory neurons (*Gad2*+) were subsetted and re-clustered as before to identify neuronal subtypes. Following clustering, any cell cluster lacking significant marker genes (FindMarkers) were removed, resulting in the final processed dataset (49,010 cells). Differential expression analysis between DR and NR cells at each time point was performed on pseudobulk samples and individual cells, using edgeR glmLRT test (*P*_adj_ < 0.1) and MAST (*P*_adj_ < 0.01), respectively.

#### CUT&RUN data processing and analysis

CUT&RUN sequencing data were processed with a custom bash script that involves the following steps: adapter trimming (cutadapt^73^ -q 30), Bowtie2^74^ alignment to mm10 (--dovetail --very-sensitive-local --nounal --no-mixed --no-discordant --phred33), duplicate read removal (Picard MarkDuplicates) with Picard (http://broadinstitute.github.io/picard/), mapping quality (samtools^75^ view -q 40), fragment length filtering (deepTools^76^ alignmentSieve --maxFragmentLength 120), and track generation (deepTools bamCoverage --binSize 1 --normalizeUsing CPM). Peaks were called on individual sample BAM files with MACS2^77^ callpeak at a *q*-value threshold of 0.05 using the IgG BAM file as a local background control.

DiffBind^78^ was used to perform CUT&RUN differential peak-calling, and also to identify consensus peaks present in 2 of 3 biological replicates for GR and NFIA across cortical cell types (Fig. 2g). edgeR^79^ was applied within DiffBind to identify differential GR sites, using the following parameters: 3T3 cell vehicle versus dexamethasone GR CUT&RUN (*P*_adj_ < 0.1); astrocyte DR versus NR GR CUT&RUN (*P*_adj_ < 0.01). Tracks of IgG, GR, or NFIA CUT&RUN signal for individual biological replicates or merged BAM file across replicates were generated using trackViewer^80^. The location of GR and NFIA binding sites (Extended Data Fig. 10e,i) relative to the NCBI RefSeq mm10 gene annotation was calculated using ChIPseeker^81^, and sites were then separated into gene distal (>500 bp from the transcription start site (TSS)) or promoter (±500 bp around the TSS) elements for motif analysis. Motif enrichment analysis of GR and NFIA sites was performed with HOMER^82^, using standard parameters. DeepTools plotHeatmap or plotProfile were used to visualize IgG, GR or NFIA CUT&RUN signal, representing CPM-normalized bigwig files for pooled samples (Fig. 2d, Extended Data Figs. 9g and 10d) or individual replicates (Extended Data Figs. 7e, 8d and 10h). Intersections between astrocyte GR binding sites and mouse GR binding sites detected by GR chromatin immunoprecipitation with sequencing (ChIP–seq) in published datasets (kidney^83^, liver^84^, primary brown preadipocytes^85^, embryonic fibroblasts^86^, mammary gland^87^ and primary bone marrow-derived macrophages^88^) were calculated with BEDtools^89^ intersect, as shown in Extended Data Fig. 8f. BETA^90^ (basic mode, -d 200000) was used to assess whether GR sites and GR-dependent ATAC sites are over-represented near P21 astrocyte GR-regulated genes (*P*_adj_ < 0.05) relative to non-differential, expressed genes (Extended Data Fig. 13a,c,d). In addition, BETA was used to calculate GR binding enrichment score for astrocyte experience-responsive genes identified in the SHARE-seq dataset (Fig. 2f and Extended Data Fig. 9a–c), which were defined as described above. To identify whether GR binding occurs at ATAC sites that correlate with astrocyte experience-responsive genes (Extended Data Fig. 9d–f), the correlation between the accessibility of ATAC sites within 500,000 bp of the TSS of experience-responsive genes and their expression was calculated with Signac LinkPeaks. Pearson correlation coefficients and *P* values were then segregated into 4 groups based on: (1) whether the target gene was suppressed or induced by experience; and (2) whether or not the ATAC site overlapped GR binding. Additionally, these 4 groups of ATAC sites were compared on the basis of their distance to the correlated gene TSS (Extended Data Fig. 9f).

#### ATAC-seq data processing and analysis

ATAC-seq data were processed using a standard pipeline established by ENCODE (https://github.com/ENCODE-DCC/atac-seq-pipeline) with default parameters. ATAC-seq differential peak-calling between GR-con and GR-KO astrocytes was performed with edgeR in DiffBind, using the following parameters: *P*_adj_ < 0.05, abs(log_2_(FC)) > 0.25. DeepTools plotHeatmap was used to plot ATAC CPM for individual biological replicates at GR-regulated ATAC sites (Extended Data Fig. 13e,f). De novo motif analysis of GR-regulated ATAC sites was performed with HOMER, using standard parameters.

#### Bulk nuclear RNA-seq data processing and analysis

RNA-seq reads were mapped to mm10 using STAR^91^ with default parameters. The number of reads mapping to gene bodies (exons and introns) was quantified with Subread featureCounts^92^, using the mm10 UCSC refGene annotation. Differential gene expression between NR GR-con and NR GR-KO astrocytes at each time point (P14, P21 and P28) was performed with DESeq2 (*P*_adj_ < 0.05, abs(log_2_(FC)) > 0.1) and shown in Fig. 3c. Differential gene expression between NR GR-con and DR GR-con astrocytes at each time point (P14, P21 and P28) was performed with DESeq2 (*P*_adj_ < 0.05) and shown in Extended Data Fig. 12d. For comparisons of P21 astrocyte GR-regulated genes with P21 astrocyte experience-regulated genes (Extended Data Fig. 12e), astrocyte maturation genes across other mouse brain regions (Extended Data Fig. 12h), human astrocyte maturation genes (Fig. 3k), or P21 astrocyte GR-regulated genes detected by snRNA-seq (Extended Data Fig. 18c), a relaxed set of GR-regulated genes were called without a log-fold change threshold (DESeq2, *P*_adj_ < 0.05).

To identify genes that change expression across V1 astrocyte development under normal conditions (Fig. 3d and Extended Data Fig. 12g), differential gene expression between P21 GR-con and P14 GR-con astrocytes was performed with DESeq2 (*P*_adj_ < 0.05, abs(log_2_(FC)) > 0.3). Differentially expressed genes were then hierarchically clustered on the basis of mean expression across time points (P14, P21 and P28) and GR status (GR-con, GR-KO) using the ward.D2 algorithm in pheatmap and split into 4 clusters with cutree = 4 (shown in Fig. 3d). The expression of genes in 2 of these 4 clusters was altered in GR-KO astrocytes and classified as GR-dependent. ClusterProfiler^93^ was then used to identify enrichment of GO biological process terms in GR-independent and GR-dependent astrocyte maturation genes (enrichGO, *P*_adj_ < 0.05).

#### Mouse developmental astrocyte snRNA-seq analysis

Mouse brain astrocyte snRNA-seq data^39^ containing 68,485 astrocytes across 5 brain regions (whole cortex, motor cortex, PFC, striatum and thalamus) and 6 time points (E18.5, P4, P14, P32, P90 and 90 weeks old (not shown)) were accessed from the Broad Institute Single Cell Portal (https://singlecell.broadinstitute.org/single_cell/study/SCP2719/a-multi-region-transcriptomic-atlas-of-developmental-cell-type-diversity-in-mouse-brain). Gene counts were normalized and log-transformed (Seurat NormalizeData) and then scaled (ScaleData). Linear dimensionality reduction was performed by principal component analysis (runPCA, npcs = 50), and the snRNA data were visualized with UMAP (runUMAP). The average expression level of P21 astrocyte GR-activated and GR-repressed genes identified by bulk RNA-seq (DESeq2, *P*_adj_ < 0.05, abs(log_2_(FC)) > 0.1) was calculated for each cell in this snRNA-seq dataset using the Seurat AddModuleScore function. The module score expression of GR-activated and GR-repressed genes in astrocytes from different brain regions were shown by UMAP (Fig. 3e) and line plot (Extended Data Fig. 12i,j). To identify genes that change expression across astrocyte development de novo, pseudobulk differential expression analysis comparing the P32 and P4 time point for each brain region was performed using DESeq2, using the following parameters: *P*_adj_ < 0.05, abs(log_2_(FC)) > 1. Significant genes were then intersected with P21 astrocyte GR-activated and GR-repressed genes identified by bulk RNA-seq (DESeq2 *P*_adj_ < 0.05), and the number of overlapping genes was shown in Extended Data Fig. 12h.

#### Human brain multiome analysis

Human brain single-nucleus multiome data^42^ containing 232,328 cells across 38 samples spanning whole cortex (first trimester only), V1, and PFC from 5 time points (first trimester, second trimester, third trimester, infancy and adolescence) were accessed from Dryad (10.5061/dryad.2280gb612) and loaded into Signac/Seurat. Peaks were re-called on the entire dataset after grouping cells by time point using MACS2 (CallPeaks), and the presence of transcription factor binding motifs (JASPAR 2020 database) within these peaks was assigned (AddMotifs). ChromVAR was used to calculate the background-corrected, genome-wide accessibility of all transcription factor binding motifs within individual cells, as done previously for SHARE-seq. Differential ChromVAR transcription factor motif scores between adolescence and first trimester cells for each cell type were calculated using MAST within the FindMarkers function. For cases in which a cell type at adolescence was not present in the first trimester, the precursor cell type was used for analysis (for example, for L2/3 IT, L4IT, L5IT and L6IT neurons, the corresponding first trimester cell types were radial glia, intermediate progenitor cells, newborn excitatory neurons, and immature IT neurons). The top 2 unique differentially accessible transcription factor motifs (by *P*_adj_ value) between adolescence and first trimester for each cell type are shown in a heat map in Fig. 3g, in which each row is scaled to the maximal within-row −log10(*P*_adj_) value.

To identify human astrocyte maturation gene programs, pseudo-bulk differential expression analysis between adolescent and first trimester astrocytes was performed using DESeq2 with the following parameters: *P*_adj_ < 0.01, abs(log_2_(FC)) > 1. Differentially expressed genes were then hierarchically clustered (shown in Fig. 3i and Extended Data Fig. 14f), on the basis of mean gene expression level across time points, using the ward.D2 algorithm in pheatmap and split into 4 clusters with cutree = 4. Human astrocyte differentially expressed genes were then overlapped with P21 mouse astrocyte GR-regulated genes (DESeq2, *P*_adj_ < 0.05) with known human orthologues. Signac LinkPeaks was used to identify ATAC peaks between 5,000 to 300,000 bp of the TSS that positively correlate (Pearson’s correlation, *P* < 0.001) in accessibility with the expression level of genes in each cluster. Motif enrichment analysis was performed on correlated ATAC peaks using Signac FindMotifs, and the top 5 enriched motifs for each gene cluster are shown in Extended Data Fig. 14g. To identify ATAC sites that change accessibility across human astrocyte development, pseudo-bulk differential accessibility analysis between adolescent and first trimester astrocytes was performed using DESeq2 with the following parameters: *P*_adj_ < 0.05, abs(log_2_(FC)) > 1. Differentially accessible regions were then hierarchically clustered (shown in Fig. 3j and Extended Data Fig. 14j), on the basis of mean chromatin accessibility across time points, using the ward.D2 algorithm in pheatmap and split into 4 clusters with cutree = 4. Human astrocyte differentially accessible regions were then overlapped with P14 mouse astrocyte GR binding sites that were lifted over from mm10 to hg38 using UCSC LiftOver.

#### snRNA-seq data processing and analysis

snRNA-seq data were processed using the Cell Ranger Count pipeline (v8.0.1) with a custom mm10 gtf file containing the eGFP sequence for viral read mapping. Default parameters were used to align reads, count transcripts, and demultiplex nuclei. Individual Cell Ranger output files for each sample were loaded into Seurat (Read10X), and nuclei with fewer than 500 gene counts were removed. DoubletFinder^94^ was then used to predict and remove doublets from each sample, and nuclei with greater than 70,000 gene counts were further removed. Seurat objects for each individual sample were then merged, and downstream normalization, scaling, dimensionality reduction, and Louvain clustering steps were performed, as described above for SHARE-seq, to identify principal cell types and excitatory and inhibitory neuron subtypes. During clustering, nuclei clusters lacking significant marker genes (FindMarkers) were removed, resulting in the final processed dataset (102,330 cells). Pseudobulk differential expression analysis between NR GR-con and GR-KO samples for each cell type was performed using the edgeR glmLRT test (*P*_adj_ < 0.05). Differentially expressed genes in excitatory neuron subtypes were separated into cell type-shared (differential in ≥2 cell types) or cell-type-specific (differential in 1 cell type), and ClusterProfiler was used to identify enrichment of GO biological process terms in the differentially expressed genes shared across excitatory neuron subtypes (enrichGO, *P*_adj_ < 0.05) (Fig. 4k,l). To compare the expression of astrocyte GR-dependent genes between NR and DR conditions, the scaled, mean expression of NR astrocyte GR-dependent genes (edgeR glmLRT *P*_adj_ < 0.05) was calculated for each experimental group (NR GR-con, NR GR-KO, DR GR-con and DR GR-KO) and visualized on box plots in Fig. 4n.

### Reporting summary

Further information on research design is available in the Nature Portfolio Reporting Summary linked to this article.

## Online content

Any methods, additional references, Nature Portfolio reporting summaries, source data, extended data, supplementary information, acknowledgements, peer review information; details of author contributions and competing interests; and statements of data and code availability are available at 10.1038/s41586-026-10512-9.

## Supplementary information

Supplementary Figures This file contains Supplementary Figs. 1 and 2. Supplementary Fig. 1. Full scans of blots shown in Extended Data Fig. 11b. Supplementary Fig. 2. Full scans of blots shown in Extended Data Fig. 11d.

Reporting Summary

Supplementary Table 1 Cell numbers for V1 SHARE-seq. Number of cells for each cell type and biological replicate in postnatal V1 SHARE-seq experiment.

Supplementary Table 2 Differential genes for developmental V1 SHARE-seq. edgeR RNA comparisons for each cell type and time point in mouse V1 development.

Supplementary Table 3 Differential peaks for developmental V1 SHARE-seq. edgeR ATAC comparisons for each cell type and time point in mouse V1 development.

Supplementary Table 4 MERFISH gene panel. Custom 500 gene panel for V1 MERFISH assay.

Supplementary Table 5 Mouse serum hormone LC–MS transitions. Transitions used for detection and analysis of aldosterone, cortisol, corticosterone and T4.

Supplementary Table 6 Differential peaks for astrocyte GR CUT&RUN. DiffBind output for GR CUT&RUN from P14 DR and NR V1 astrocytes.

Supplementary Table 7 Consensus peaks for astrocyte NFIA CUT&RUN. Consensus peaks (2 of 3 biological replicates) for NFIA CUT&RUN from P14 NR V1 astrocytes.

Supplementary Table 8 Differential genes for V1 astrocyte development RNA-seq. DESeq2 RNA comparisons between V1 GR-KO and GR-con astrocytes and between V1 NR and DR GR-con astrocytes at 3 time points (P14, P21 and P28) during mouse development.

Supplementary Table 9 Differential peaks for V1 astrocyte ATAC-seq. edgeR ATAC comparisons between V1 NR GR-KO and GR-con astrocytes at P21.

Supplementary Table 10 Differential genes for V1 astrocyte GR-KO snRNA-seq. edgeR snRNA-seq pseudo-bulk comparisons between P21 NR astrocyte GR-KO and GR-con groups for each cell type.

Peer Review File

## Source data

Source Data Fig. 2

Source Data Fig. 3

Source Data Fig. 4

Source Data Fig. 5

## Data Availability

All sequencing data generated in this study have been deposited in GEO (GSE306265). MERFISH data are available at 10.6084/m9.figshare.29971903 (ref. ^95^). The following publicly available datasets were also analysed: mouse kidney GR ChIP–seq (GSE115368), mouse liver GR ChIP–seq (GSE72087), mouse primary brown preadipocyte GR ChIP–seq (GSE76619), mouse embryonic fibroblast GR ChIP–seq (GSE69947), mouse mammary gland GR ChIP–seq (GSE74826), mouse primary bone marrow-derived macrophage GR ChIP–seq (GSE99887), human brain development single-nucleus multiome-seq (10.5061/dryad.2280gb612 (ref. ^96^)), and mouse astrocyte development snRNA-seq (https://singlecell.broadinstitute.org/single_cell/study/SCP2719/a-multi-region-transcriptomic-atlas-of-developmental-cell-type-diversity-in-mouse-brain). The following publicly available databases were used for data processing: NCBI mm10 genome and RefSeq gene annotation (https://www.ncbi.nlm.nih.gov/datasets/genome/GCF_000001635.20), NCBI hg38 genome and RefSeq gene annotation (https://www.ncbi.nlm.nih.gov/datasets/genome/GCF_000001405.26), and UCSC mm10 refGene annotation (http://hgdownload.soe.ucsc.edu/goldenPath/mm10/bigZips/genes). Source data are provided with this paper.

## Code availability

Custom scripts can be found at https://github.com/brunogegenhuber/GR_gene_reg.
