# Supplementary material for: Astrocyte glucocorticoid receptor signalling restricts neuronal plasticity
Source: Nature. 2026 May 20;655(8125):1233–41. doi: 10.1038/s41586-026-10512-9 (PMC13421323; doi:10.1038/s41586-026-10512-9)
Supplement: Supplementary file 1 — This file contains Supplementary Figs. 1 and 2. Supplementary Fig. 1. Full scans of blots shown in Extended Data Fig. 11b. Supplementary Fig. 2. Full scans of blots shown in Extended Data Fig. 11d. [file 41586_2026_10512_MOESM1_ESM.pdf]

nature portfolio

https://doi.org/10.1038/s41586-026-10512-9

Supplementary information

Astrocyte glucocorticoid receptor signalling restricts neuronal plasticity

In the format provided by the authors and unedited

Supplementary Fig. 1

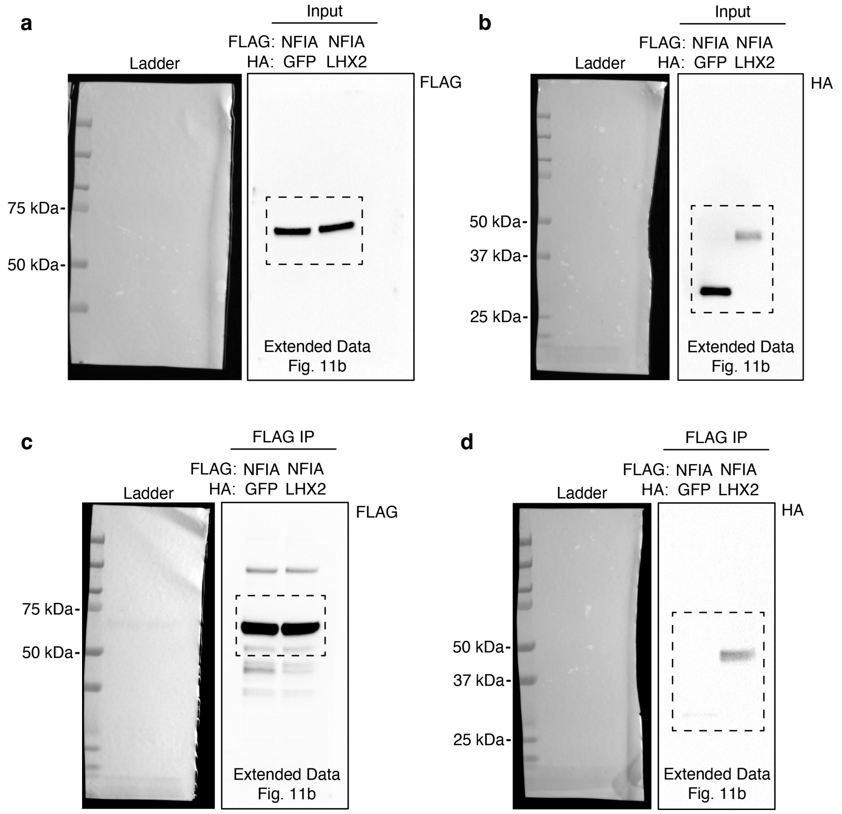

Supplementary Fig. 1. Full scans of blots shown in Extended Data Fig. 11b. a-b, (left) Ladder image; (right) input fractions from HEK293T cells over-expressing FLAG-NFIA/HA-NLS-GFP or FLAG-NFIA/HA-LHX2 immunoblotted for FLAG (**a**) or HA (**b**). **c-d,** (left) Ladder image; (right) FLAG immunoprecipitation (IP) fractions from HEK293T cells over-expressing FLAG-NFIA/HA-NLS-GFP or FLAG-NFIA/HA-LHX2 immunoblotted for FLAG (**c**) or HA (**d**). Insets are cropped images shown in Extended Data Fig. 11b.

Supplementary Fig. 2

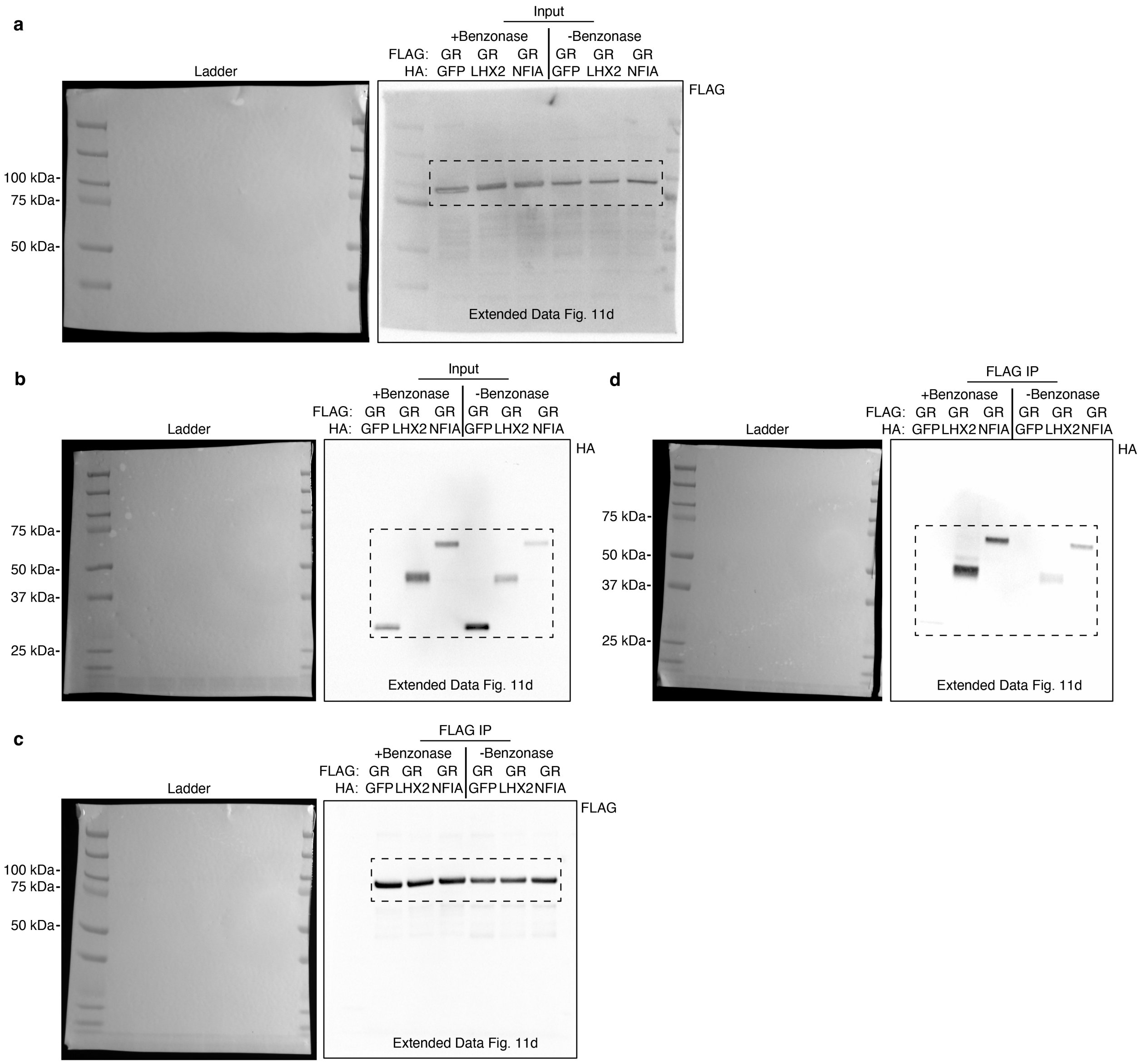

Supplementary Fig. 2. Full scans of blots shown in Extended Data Fig. 11d. a-b, (left) Ladder image; (right) input fractions extracted with (+) or without (-) benzonase from HEK293T cells over-expressing FLAG-GR/HA-NLS-GFP, FLAG-GR/HA-LHX2, or FLAG-GR/HA-NFIA immunoblotted for FLAG (**a**) or HA (**b**). **c-d,** (left) Ladder image; (right) FLAG IP fractions extracted with (+) or without (-) benzonase from HEK293T cells over-expressing FLAG-GR/HA-NLS-GFP, FLAG-GR/HA-LHX2, or FLAG-GR/HA-NFIA immunoblotted for FLAG (**c**) or HA (**d**). Insets are cropped images shown in Extended Data Fig. 11d.
