# Supplementary material for: Astrocyte glucocorticoid receptor signalling restricts neuronal plasticity
Source: Nature. 2026 May 20;655(8125):1233–41. doi: 10.1038/s41586-026-10512-9 (PMC13421323; doi:10.1038/s41586-026-10512-9)
Supplement: Supplementary file 2 — Reporting Summary [file 41586_2026_10512_MOESM2_ESM.pdf]

nature portfolio

Corresponding author(s): Michael E. Greenberg

Last updated by author(s): Mar 20, 2026

Reporting Summary

Nature Portfolio wishes to improve the reproducibility of the work that we publish. This form provides structure for consistency and transparency in reporting. For further information on Nature Portfolio policies, see our Editorial Policies and the Editorial Policy Checklist.

Statistics

For all statistical analyses, confirm that the following items are present in the figure legend, table legend, main text, or Methods section.

n/a	Confirmed	
☐	☒	The exact sample size (*n*) for each experimental group/condition, given as a discrete number and unit of measurement
☐	☒	A statement on whether measurements were taken from distinct samples or whether the same sample was measured repeatedly
☐	☒	The statistical test(s) used AND whether they are one- or two-sided<br>*Only common tests should be described solely by name; describe more complex techniques in the Methods section.*
☐	☒	A description of all covariates tested
☐	☒	A description of any assumptions or corrections, such as tests of normality and adjustment for multiple comparisons
☐	☒	A full description of the statistical parameters including central tendency (e.g. means) or other basic estimates (e.g. regression coefficient) AND variation (e.g. standard deviation) or associated estimates of uncertainty (e.g. confidence intervals)
☐	☒	For null hypothesis testing, the test statistic (e.g. *F*, *t*, *r*) with confidence intervals, effect sizes, degrees of freedom and *P* value noted<br>*Give P values as exact values whenever suitable.*
☒	☐	For Bayesian analysis, information on the choice of priors and Markov chain Monte Carlo settings
☐	☒	For hierarchical and complex designs, identification of the appropriate level for tests and full reporting of outcomes
☐	☒	Estimates of effect sizes (e.g. Cohen's *d*, Pearson's *r*), indicating how they were calculated

Our web collection on statistics for biologists contains articles on many of the points above.

Software and code

Policy information about availability of computer code

Data collection

V1 SHARE-seq: SHARE-seq fastq files were processed using the SHARE-seq-alignmentV2 pipeline (https://github.com/masai1116/SHARE-seq-alignmentV2), using default parameters.

V1 MERFISH: MERFISH data were processed on the MERSCOPE Instrument cell segmentation using a watershed algorithm based on DAPI and PolyT staining. Individual images were then loaded into MERSCOPE Visualizer (v2.3.3330.0) and a region of interest (ROI) around V1 was drawn to subset cells for downstream analysis.

Brain and cell line CUT&RUN: Adapters and low-quality basecalls were trimmed using cutadapt (v4.1). Paired-end reads were aligned to mm10 using bowtie2 (v2.3.4.3). Duplicate reads were removed with picard (v2.27.5). Reads were filtered using SAMtools (v1.15.1). Peaks were called using MACS2 (v2.2.7.1). Bigwig tracks were generated using deepTools (v3.5.1).

P21 astrocyte GR-KO ATAC-seq: ATAC fastq files were processed using the ENCODE ATAC-seq pipeline (v2.2.0), with parameters: atac.auto_detect_adapter = TRUE, atac.multimapping = 0, atac.pval_thresh = 0.01. Bigwig tracks were generated from the filtered BAM files using deepTools (v3.5.1).

V1 astrocyte nuclear RNA-seq: RNA-seq fastq files were mapped using STAR (v2.7.9a). The number of reads mapping to gene bodies (exons and introns) was quantified with Subread featureCounts (v2.0.0).

Single-nucleus RNA-seq: Fastq files were processed using the Cell Ranger pipeline (v8.0.1) with the mm10 ENSEMBL gtf file. Default parameters were used to align reads, count unique transcripts, and filter high-quality nuclei.

Data analysis	The following software packages were used to analyze and visualize the data: Imaris (v10.2), pCLAMP (v10.6), MATLAB (v25.1), GraphPad Prism (v10), deepTools (v3.5.1), HOMER (v4.11.1), BEDTools (v2.27.1), clusterProfiler (v4.8.3), trackViewer (v1.36.2), DESeq2 (v1.40.2), DiffBind (v3.10.1), Seurat (v5.3.0), SingleCellExperiment (v1.22.0), ComplexHeatmap (v2.16.0), ChIPseeker (v1.36.0), eulerr (v7.0.0), ggplot2 (v3.5.2), pheatmap (v1.0.12), BETA (v1.0.7), edgeR (v3.42.4), R stats (v4.3.1), chromVAR (v1.22.1), Signac (v1.14.0), STalign (v1.0), Sony SH800S Cell Sorter Software (v2.1.6). Custom scripts used to analyze data are available at https://github.com/brunogegenhuber/GR_gene_reg.

For manuscripts utilizing custom algorithms or software that are central to the research but not yet described in published literature, software must be made available to editors and reviewers. We strongly encourage code deposition in a community repository (e.g. GitHub). See the Nature Portfolio guidelines for submitting code & software for further information.

Data

Policy information about availability of data

All manuscripts must include a data availability statement. This statement should provide the following information, where applicable:

- Accession codes, unique identifiers, or web links for publicly available datasets
- A description of any restrictions on data availability
- For clinical datasets or third party data, please ensure that the statement adheres to our policy

All sequencing data generated in this study have been deposited in GEO (GSE306265). MERFISH data are available at: https://doi.org/10.6084/m9.figshare.29971903. The following publicly available datasets were also analyzed: mouse kidney GR ChIP-seq (GSE115368), mouse liver GR ChIP-seq (GSE72087), mouse primary brown preadipocyte GR ChIP-seq (GSE76619), mouse embryonic fibroblast GR ChIP-seq (GSE69947), mouse mammary gland GR ChIP-seq (GSE74826), mouse primary bone marrow-derived macrophages (GSE99887), human brain development single-nucleus multiome-seq (https://doi.org/10.5061/dryad.2280gb612), and mouse astrocyte development snRNA-seq (https://singlecell.broadinstitute.org/single_cell/study/SCP2719/a-multi-region-transcriptomic-atlas-of-developmental-cell-type-diversity-in-mouse-brain). The following publicly available databases were used for data processing: NCBI mm10 genome and RefSeq gene annotation (https://www.ncbi.nlm.nih.gov/datasets/genome/GCF_000001635.20), NCBI hg38 genome and RefSeq gene annotation (https://www.ncbi.nlm.nih.gov/datasets/genome/GCF_000001405.26), and UCSC mm10 refGene annotation (http://hgdownload.soe.ucsc.edu/goldenPath/mm10/bigZips/genes).

Research involving human participants, their data, or biological material

Policy information about studies with human participants or human data. See also policy information about sex, gender (identity/presentation), and sexual orientation and race, ethnicity and racism.

Reporting on sex and gender	NA
Reporting on race, ethnicity, or other socially relevant groupings	NA
Population characteristics	NA
Recruitment	NA
Ethics oversight	NA

Note that full information on the approval of the study protocol must also be provided in the manuscript.

Field-specific reporting

Please select the one below that is the best fit for your research. If you are not sure, read the appropriate sections before making your selection.

☒ Life sciences ☐ Behavioural & social sciences ☐ Ecological, evolutionary & environmental sciences

For a reference copy of the document with all sections, see nature.com/documents/nr-reporting-summary-flat.pdf

Life sciences study design

All studies must disclose on these points even when the disclosure is negative.

Sample size	Sample sizes were determined by several factors: 1) number of replicates previously used for these methods in the literature, 2) cost of sequencing and animal maintenance, and 3) expected variability between samples for each assay after preliminary experiments.
Data exclusions	No data were excluded.
Replication	The number of biological replicates per condition for each experiment are listed below. All attempts at replication were successful.<br><br>Postnatal V1 SHARE-seq: n=4<br>Postnatal V1 MERFISH: n=2<br>Serum CORT ELISA: n=6-8

Serum LC-MS: n=8-15
Cell line GR CUT&RUN: n=2
DR vs. NR GR CUT&RUN: n=3
Vgat+, Vglut+, Olig2+ GR and NFIA CUT&RUN: n=3
GR-KO IF staining: n=5-6
GR-KO CUT&RUN: n=2
V1 astrocyte morphology IF staining: n=21-26 cells
V1 astrocyte astrocyte GR-KO RNA-seq: n=3
V1 astrocyte astrocyte GR-KO ATAC-seq: n=3
V1 astrocyte GR-KO PNN IF staining: n=5-6
V1 astrocyte GR-KO Acan IF staining: n=5-6
V1 astrocyte GR-KO Syt2/CB1R staining: n=5-7
V1 astrocyte GR-KO Vglut1/Homer1 staining: n=5-8
V1 astrocyte GR-KO mIPSCs/mEPSCs: n=15-22 cells
V1 astrocyte GR-KO PV-evoked IPSCs: n=15-17 cells
V1 astrocyte GR-KO snRNA-seq: n=2
V1 DR CORT PNN IF staining: n=4-5
V1 astrocyte GR-KO ODP: n=4-5

For V1 CUT&RUN and ATAC experiments, brain tissue was pooled from 3-5 animals per biological replicate. For V1 astrocyte nuclear RNA-seq experiments, brain tissue was pooled from 2 animals per biological replicate.

Randomization

For all experiments, animals or cells were randomly assigned to experimental groups.

Blinding

All imaging, electrophysiology , and in vivo recording experiments were performed by an investigator blinded to experimental condition. Blinding was not relevant for bioinformatic analyses because knowledge of sample identity is required for statistical testing and data visualization. Investigators were blinded to group allocation during data collection of genomic experiments.

Reporting for specific materials, systems and methods

We require information from authors about some types of materials, experimental systems and methods used in many studies. Here, indicate whether each material, system or method listed is relevant to your study. If you are not sure if a list item applies to your research, read the appropriate section before selecting a response.

Materials & experimental systems

n/a	Involved in the study
☐	☒ Antibodies
☐	☒ Eukaryotic cell lines
☒	☐ Palaeontology and archaeology
☐	☒ Animals and other organisms
☒	☐ Clinical data
☒	☐ Dual use research of concern
☒	☐ Plants

Methods

n/a	Involved in the study
☒	☐ ChIP-seq
☐	☒ Flow cytometry
☒	☐ MRI-based neuroimaging

Antibodies

Antibodies used

GR CUT&RUN Ab #1: Invitrogen MA1-510 (Clone BuGR2, Lot 3212042)
GR CUT&RUN Ab #2: Invitrogen PA1-511A (Lot ZK404106)
IgG CUT&RUN Ab: Cell Signaling Technology 2729S (Lot 11)
Nfia CUT&RUN Ab: Invitrogen PA5-52252 (Lot ZF4346665)
GR IF Ab: Cell Signaling Technology 12041S (Lot 7)
Sox9 IF Ab: R&D Systems AF3075 (Lot WL052309L)
GFP IF Ab: Aves Labs GFP-1020 (Lot GFP917979)
Rabbit PV IF Ab: Swant PV27a (Lot 2014)
Guinea Pig PV IF Ab: Synaptic Systems 195-308 (Clone Gp58E1, Lot 1-8)
Acan IF Ab: Sigma AB1031 (Lot 4113382)
NeuN IF Ab: EMD Millipore ABN78 (Lot 4203297)
Syt2 IF Ab: ZIRC ZDB-ATB-081002-25 (Lot 122022)
CB1R IF Ab: Frontier Institute MSFR100630 (No lot #)
Homer1 IF Ab: Synaptic Systems 160003 (Lot 3-96)
Vglut1 IF Ab: Sigma AB5905 (Lot 4141952)
Anti-FLAG WB Ab: Sigma Aldrich F1804 (Clone M2, Lot 0000502386)
Anti-HA WB Ab: Cell Signaling Technology 3724 (Lot 13)
Anti-rabbit IgG, HRP-conjugated WB Ab: Cell Signaling Technology 7074 (lot 34)
Anti-mouse IgG, HRP-conjugated WB Ab: Cell Signaling Technology 7076 (lot 39)
Anti-rabbit Dylight 405:Jackson ImmunoResearch 711-475-152 (Lot 171728)
Anti-chicken Alexa Fluor 488: Jackson ImmunoResearch 703-545-155 (Lot 168728)
Anti-rabbit Alexa Fluor 594: Life Technologies A21207 (Lot 2897798)
Anti-mouse Alexa Fluor 594: Jackson ImmunoResearch 715-585-150 (Lot 160632)

	Anti-guinea pig Alexa Fluor 647: Jackson ImmunoResearch 706-605-148 (Lot 169548)<br>Anti-goat Alexa Fluor 647 : Life Technologies A21447 (Lot 2544596)<br>Anti-mouse Alexa Fluor 647: Life Technologies A31571 (Lot 2892424)
Validation	All antibodies used for WB and IF staining have been validated by the manufacturer for this purpose and have been used in prior publications: FLAG (Wu et al. 2026 Nature), HA (Gu et al. 2023 Science), Syt2 (Jézéquel et al. 2025 Nat Commun), CB1R (Jézéquel et al. 2025 Nat Commun), Sox9 (Burda et al. 2022 Nature), PV (Kobayashi et al. 2015 Neuron), Acan (Nguyen et al. 2021 Cell), Vglut1 (Stogsdill et al. 2017 Nature), Homer1 (Abraira et al. 2017 Cell). The GR antibody used for IF was validated in GR-KO cells in Extended Data Fig. 8 The GR antibodies used for CUT&RUN were first validated in DEX-treated 3T3 cells (Extended Data Fig. 7) and further validated in GR-KO astrocytes (Extended Data Fig. 9). The Nfia antibody used for CUT&RUN was previously validated in a prior publication: Qin et al. 2022 Nat Genet.

Eukaryotic cell lines

Policy information about cell lines and Sex and Gender in Research

Cell line source(s)	NIH/3T3 cells (ATCC, CRL-1658), HEK293T cells (ATCC, CRL-3216)
Authentication	The cell line used was authenticated by the vendor using STR profiling and morphology.
Mycoplasma contamination	3T3 and HEK293T cells were not tested in-house for mycoplasma contamination.
Commonly misidentified lines<br>(See ICLAC register)	No commonly misidentified lines were used.

Animals and other research organisms

Policy information about studies involving animals; ARRIVE guidelines recommended for reporting animal research, and Sex and Gender in Research

Laboratory animals	Animals were maintained in a reverse 12-h light/12-h dark cycle (22:00-10:00) in a temperature- and humidity-controlled environment and provided food and water ad libitum. For DR conditions, animals were maintained in chronic darkness until collection. To control for circadian rhythm and to prevent light stimulation of DR animals, mice were harvested under red light between 10:00-12:00 (Zeitgeiber Time 12-14) for all genomic, imaging, ELISA, and LC-MS experiments.<br><br>The following mouse lines were used: C57BL/6 (Jackson Laboratory, 000664), GRfl/fl (Jackson Laboratory, 021021), Vglut1Cre (Jackson Laboratory, 023527), VgatCre (Jackson Laboratory, 028862), Olig2Cre (Jackson Laboratory, 025567), Sun1-GFPfl/fl (Jackson Laboratory, 021039), and PvalbFlpO (Jackson Laboratory, 022730).<br><br>For the developmental SHARE-seq and MERFISH experiments, animals were harvested between P7 and P35.<br>For V1 astrocyte CUT&RUN, animals were harvested at P14.<br>For V1 astrocyte ATAC-seq, animals were harvested at P21.<br>For V1 astrocyte RNA-seq, animals were harvested at P14, P21, and P28.<br>For V1 snRNA-seq, animals were harvested at P21.<br>For ELISA and LC-MS experiments, animals were harvested at P10, P14, P21, and P28.<br>For imaging experiments, animals were collected at P21, P28, P35, and 8-12-weeks of age (adult).<br>For electrophysiology experiments, animals were patched between P33 and P35.<br>For adult ODP experiments, animals were recorded between P60-P70.
Wild animals	No wild animals were used in this study.
Reporting on sex	Male and female mice were randomly assigned to experimental groups and used in similar proportions for all experiments.
Field-collected samples	No field-collected samples were used in this study.
Ethics oversight	Animal care and surgical procedures were approved and overseen by the Harvard University Institutional Animal Care and Use Committee and the Harvard Center for Comparative Medicine or the Institutional Animal Care and Use Committee at Boston Children's Hospital.

Note that full information on the approval of the study protocol must also be provided in the manuscript.

Plants

Seed stocks: NA

Novel plant genotypes: NA

Authentication: NA

Flow Cytometry

Plots

Confirm that:

☒ The axis labels state the marker and fluorochrome used (e.g. CD4-FITC).

☒ The axis scales are clearly visible. Include numbers along axes only for bottom left plot of group (a 'group' is an analysis of identical markers).

☒ All plots are contour plots with outliers or pseudocolor plots.

☒ A numerical value for number of cells or percentage (with statistics) is provided.

Methodology

Sample preparation: The following protocol was used to isolate nuclei from early postnatal mice (P14-P28) prior to downstream CUT&RUN, ATAC-seq, bulk RNA-seq, and snRNA-seq experiments. Mice were deeply anesthetized with isoflurane. Sections of 1 mm spanning V1 were collected in an adult mouse brain matrix (Alto) on ice, and V1 was microdissected. For bulk RNA-seq and snRNA-seq experiments, tissue samples were immediately flash-frozen in a dry ice ethanol bath and stored at -80°C prior to nuclei extraction, whereas tissue samples were processed the day of collection for CUT&RUN and ATAC-seq. After thawing or dissecting samples, V1 tissue was transferred to a 1.5 mL tube containing 350 µL of cold homogenization buffer (250 mM sucrose, 25 mM KCl, 5 mM MgCl2, 20 mM tricine-KOH pH 7.8 supplemented with 1X PIC, 1 mM DTT, 0.15 mM spermine and 0.5 mM spermidine). For RNA experiments, 0.3 U/µL (bulk RNA-seq) or 1 U/µL (snRNA-seq) of Protector RNase inhibitor (Sigma) was added. The tissue was dounced in a 2 mL glass homogenizer (Sigma D8938) with a loose pestle for 20 strokes. Samples were supplemented with 0.3% IGEPAL CA-630 and further dounced for an additional 10 strokes with a tight pestle. Homogenate was filtered through a small 40-µm strainer (pluriSelect 43-10040-50) into a 1.5 mL tube before diluting 1:1 with homogenization buffer and adding DRAQ5 (Abcam ab108410) at a 1:500 dilution. For CUT&RUN experiments, samples were additionally supplemented with 4 mM EDTA. Nuclei were then sorted by GFP and/or DRAQ5 signal using the Sony SH800S Cell Sorter (purity mode) with a 100-µm sorting chip. For CUT&RUN, 175,000 DRAQ5+/GFP+ or DRAQ5+/GFP- events were collected into 1 mL of cold CUT&RUN Wash Buffer (20 mM HEPES pH 7.5, 150 mM NaCl, 0.2% Tween-20, 0.5 mM spermidine, 0.1% bovine serum albumin [BSA], 1X PIC). For ATAC-seq, 50,000 DRAQ5+/GFP+ events were collected into 1 mL of cold ATAC-RSB (10 mM Tris-HCl pH 7.5, 10 mM NaCl, 3 mM MgCl2). For bulk RNA-seq, 80,000 DRAQ5+/GFP+ events were collected into 350 µL of cold RLT buffer (Qiagen 74004) supplemented 1:100 with β-mercaptoethanol. For snRNA-seq, 50,000 DRAQ5+/GFP- events were collected into a 1.5 mL tube (precoated with 30% BSA) containing 300 µL of cold ATAC-RSB supplemented with 1 mM DTT, 1 U/µL Protector RNase inhibitor, and 1% BSA (Sigma A8577-10mL).

Instrument: Sony SH800S Cell Sorter

Software: Sony SH800S Cell Sorter Software

Cell population abundance:
Astrocyte EGFP-KASH+ population comprised ~4-8% of singlet nuclei.
Olig2-Cre/Sun1-GFP+ population comprised ~20-25% of singlet nuclei.
Vglut1-Cre/Sun1-GFP+ population comprised ~30-40% of singlet nuclei.
Vgat-Cre/Sun1-GFP+ population comprised ~10-20% of singlet nuclei.

Gating strategy:
1) Nuclei were first gated on the BSC-A x DRAQ5-A plot.
2) Singlet nuclei were gated on the linear axis of the FSC-H x FSC-A plot.
3) GFP+ singlet nuclei were gated on the BSC-A x EGFP-A plot.
Post-sort purity was initially validated on a fluorescence microscope.

☒ Tick this box to confirm that a figure exemplifying the gating strategy is provided in the Supplementary Information.
