# Supplementary material for: Astrocyte glucocorticoid receptor signalling restricts neuronal plasticity
Source: Nature. 2026 May 20;655(8125):1233–41. doi: 10.1038/s41586-026-10512-9 (PMC13421323; doi:10.1038/s41586-026-10512-9)
Supplement: Supplementary file 13 — Peer Review File [file 41586_2026_10512_MOESM13_ESM.pdf]

nature portfolio

Peer Review File

Astrocyte glucocorticoid receptor signaling restricts neuronal plasticity

Corresponding Author: Dr Michael Greenberg

Any redactions in this file are there to maintain patient confidentiality, the confidentiality of unpublished data, or to remove third-party material.

This file contains all reviewer reports in order by version, followed by all author rebuttals in order by version.

Attachments originally included by the reviewers as part of their assessment can be found at the end of this file.

Version 0:

Reviewer comments:

Referee #1

(Remarks to the Author)
Summary

This is a well-designed and rigorously executed study that addresses a longstanding question in developmental neurobiology: how systemic signals interface with local cortical circuits to regulate critical period plasticity. By combining transcriptomic and epigenomic profiling (SHARE-seq, MERFISH, CUT&RUN, ATAC-seq, RNA-seq, snRNA-seq) with astrocyte-specific manipulations, the authors identify glucocorticoid receptor (GR) signaling in astrocytes as a driver of astrocyte maturation, extracellular matrix formation, and restriction of plasticity.

The dataset is exceptionally rich, technically sophisticated, and of broad value to the neuroscience community. The findings will interest not only developmental and glial biologists but also those working on endocrinology, stress, and psychiatric disease. The conceptual advance—linking systemic corticosterone signaling to astrocyte-driven closure of cortical plasticity—is novel and compelling.

At the same time, the manuscript makes causal claims that go beyond the presented data. In particular, the relationship between corticosterone surge and astrocyte GR activation is correlative, and the functional demonstration that GR restricts ocular dominance plasticity (ODP) is lacking. While the enthusiasm for the dataset and analysis is high, additional experiments would be necessary to fully support the central claims.

Major Concerns

Causality of corticosterone–GR signaling: The light-induced rise in corticosterone at eye opening is a key link in the proposed mechanism, but necessity and sufficiency are not tested. Adrenalectomy, pharmacological CORT supplementation, or GR antagonism would directly test this causal link.

Direct demonstration of critical period closure: The study infers effects on ODP from dark rearing and molecular markers. However, ODP requires competitive manipulation (e.g., monocular deprivation) with physiological or behavioral readouts. Without this, the claim that astrocyte GR “restricts plasticity” is premature.

Specificity of astrocyte GR action: While GR binding appears enriched in astrocytes, GR is broadly expressed. Stronger evidence is needed to exclude indirect circuit-level effects, especially since many of the neuronal changes could be secondary.

Circuit-level function: Altered PNNs and bouton densities are observed, but PV-evoked IPSCs are unchanged. Presynaptic measures (e.g., paired-pulse ratio) would more directly assess synaptic changes. The mechanistic link between astrocytic ECM regulation and PV function requires clarification.

Human data interpretation: The human extension is valuable but correlative. The claim of conserved GR-driven astrocyte maturation should be tempered unless supported by functional human tissue or organoid data.

Speculation on stress and psychiatric disease: The discussion extends to early-life stress and psychiatric vulnerability. While intriguing, these claims are not experimentally supported in the present work and should be phrased more cautiously.

Detailed Comments:

Figure 1:
- The patterns of changes in experience-dependent gene counts are remarkably unique in different cell types: the peak (P28) in non-neuronal cell types (astros, oligos, and microglia) is distinct from several types of excitatory neurons (P21 and P35) and PV interneurons (P35). This finding seems to fit well with the authors' hypothesis that "astrocytes…might be uniquely poised to take up peripheral hormones such as glucocorticoids in response to visual experience" and would benefit from further discussion and interpretation in the main text.
- The authors include very little comparative analysis of the p21 and p35 MERFISH data. Additional plots and/or text discussion of age-specific differences in MERFISH detection of experience-responsive genes would further strengthen the argument that diverse V1 cell types respond to visual input on distinct time scales.

Figure 2:
- The authors suggest that GR interacts with chromatin through tethering and cooperativity with NFIA and LHX transcription factors. However, the data supporting this claim is largely correlative and would be better supported by causal studies:
- Probe the impact of NFIA deletion on GR chromatin interactions by performing GR cut & run in astrocyte-specific NFIA-cKO vs control conditions.
- Probe the putative protein-protein interaction between NFIA and GR by performing co-immunoprecipitation in astrocytes or heterologous cell lines.

Figure 3:
- Correlate RNAseq repressed/activated genes (RNAseq, 3c-d) and repressed/activated sites (ATACseq, 2f) in GR-KO vs CON
- Does 3g show only motifs that gained accessibility (vs. lost accessibility?)
- The discussion (lines 238-247) and figures pertaining to human astrocyte developmental gene programs, it is not clear whether differentially expressed (Fig 3i) and differentially accessible (Fig 3j) gene programs c1-c4 represent a common set of 4 programs or distinct programs identified through separate hierarchical clustering of snRNAseq and ATACseq data. Based on the methods (lines 1010-1028), it seems that the latter is true. This should be clarified in the text and figures.

Figure 4:
- Since Acan is PV-specific (Fig S12d-e), how do the authors interpret the finding that an astrocyte-specific manipulation (GR-cKO) impacts Acan abundance? Perhaps other astrocyte-specific proteins (e.g., proteases) modulate its stability? If so, this point would be well-supported by direct manipulation of an astrocyte-specific ECM modulator (e.g., Mmp2) and readout of Acan abundance. This portion of the study would also be strengthened by immunohistochemical staining for other PNN components (e.g., Vcan or Tnc), which are directly changing expression in astrocytes.
- GR-cKO reduces WFA intensity on a per-cell basis in both adolescents and adults (Fig. 4a- d, Fig. S12), but the authors do not directly test whether GR-cKO impacts PVI differentiation/maturation or survival. Mean PV intensity is used as a proxy (Fig. S12f-h); however, the findings of reduced PV intensity in adolescents but not in adults are not discussed in the text. This may be relevant to interpret the reduced Syt2+ synapse counts (i.e., are individual PVIs forming fewer Syt2+ synaptic inputs, or are there fewer PVIs?). We recommend PVI and PNN counting analyses, such as the number/density of PVIs and PNNs, and the proportion of PVIs with PNNs.
- Is the intensity of light stimulation (1-4 mW/mm2) a good approximation of physiologic PV firing (Fig. 4g-h, S13f-k)? Please cite evidence.
- Given that immunohistochemical analysis showed changes in presynaptic bouton density, would paired-pulse ratio be a better electrophysiological measure of presynaptic strength changes? As the authors point out, evoked IPSCs could be compensated by postsynaptic strengthening and are thus a less specific measure of the observed presynaptic changes.
- The authors suggest that "astrocyte-dependent suppression of neuronal gene expression might be critical for circuit maturation and the closure of critical periods" (lines 310-312). This claim would be better supported by monocular deprivation experiments in animals during and post-critical period.
- Based on snRNAseq at p21, the authors suggest that the effects of GR-cKO on V1 pyramidal neurons mimic the effect of dark-rearing. However, intracortical excitatory synapses are only tested at p35 and are unchanged at this later time point. Additional assays of V1 neuron maturation/functon(e.g., synaptic staining or electrophysiological recordings) at p21 would strengthen the authors' suggestion that the neuronal effects of astrocyte-specific GR-cKO mimic dark-rearing.
- The authors show reduced Syt2+ inputs to L5 PyNs at P35. Could a similar phenomenon be present earlier in development (e.g., P21) and thereby underlie the astrocyte-dependent changes in L5IT gene expression at P21? Staining and e-phys analysis of PVI inputs to L5 neurons at an earlier age would be informative on this point.

Figure S7:
- The validation of GR-cKO is performed in p35, whereas C&R experiments are all in P14, and astrocyte morphology at p21. GR-cKO should be performed at the earliest time point (P14) at which the model is used.
Figure S8:
- How heterogeneous is the expression of Nr3c1/GR in astrocytes? This can be addressed through further analysis of

snRNAseq data, as well as the imaging dataset presented in Figure S7c (quantify the proportion of Sox9+ cells labeled with GR).

Figure S10:
- There is existing evidence that glucocorticoid signaling induces Zbtb16 upregulation in other cell types. Please see the recent paper The glucocorticoid receptor is affected by its target ZBTB16 in a dissociated manner (Galuh et al., 2025) and its cited studies.

Minor concerns:

Figure S10: Please clarify in the text whether the GR binding sites analyzed in panel A were identified from the CUT&RUN experiments performed in this study or through motif-based predictive analysis.

(Remarks on code availability)

Referee #2

(Remarks to the Author)
I co-reviewed this manuscript with one of the reviewers who provided the listed reports.

(Remarks on code availability)

Referee #3

(Remarks to the Author)
Here Gegenhuber and colleagues present data suggesting that astrocyte GR signaling is integral for the modulation of extracellular matrix (ECM) components, and as a consequence modulate neuron network experience-dependent specification of the mouse primary visual cortex during postnatal development. There have been numerous studies over the years that implicate astrocytes in synaptic plasticity and modulation of the ECM - largely focusing on early live development (synapse formation/function) or in response to acute injury (ECM component changes). The concept of bringing these two together here is compelling, and the authors provide some compelling evidence for the role of astrocytes in mediating this specific visual cortical plasticity.
As would be expected from this group, the manuscript is well written, and presents a narrative that is easy to follow, and is compelling. I have a number of concerns however (see below) largely focused on a gross oversight of providing sequencing data (even in light of Editorial Checklist confirmation that it is available), and missing code for analysis. While the validation experiments themselves look well completed, that they are based on these expansive datasets (from SHARE-seq, single nucleus RNAseq, CUT&RUN, bulk RNAseq, and MERSCOPE spatial transcriptomics) but only 6 of the more than 60 (estimated) samples are available is concerning. These, if presented and made available would elevate this manuscript from an interesting and important biological mechanism, to a toolset for the community to mine and further a great number of exciting future discoveries. Additional concerns such as a lack of QC metrics across most of these datasets (owing to their absence except for key figure panels integral to the narrative) dampen enthusiasm for the impact of these part of the overall study.

Points for consideration:
1. model of developmental plasticity - how important is neural circuit maturation in light of synaptic and network plasticity in the adult brain? Are the developmental and plasticity mechanisms different, or do the authors think that these mechanisms are similarly used in these different contexts?

2. Data availability concerns - the authors state that they completed new SHARE-seq (multiome) on mouse V1, however the only uploaded and available datasets in GSE299347 at 6 (3KO, 3CTL) bulk ATACseq datasets on purified astrocytes. Can the authors confirm if the SHARE-seq data was re-analyzed from the earlier Regev lab study, or if the data availability statement needs to be corrected and this new data uploaded the GEO and reviewed? (note this would mean 38 individual samples are missing from the GEO repository).
A review of the Editorial Policy Checklist shows that both the data availability statement and mandated accession code boxes have been ticked, but this is incorrect.
The code availability statement box has also been checked, but the custom git repo link pulls a 404 error message.
Additional sequencing data not available but presented in the manuscript and described in the methods include:
> CUT&RUN (both 3T3 cells, and isolated CNS cells). It should be noted there is a large number of samples missing here, as CUT&RUN was perform on "GR, NFIA, and IgG CUT&RUN experiments on excitatory neurons, inhibitory neurons, or oligodendrocytes, each biological replicate consisted of P14 V1 tissue pooled from 3-5 Vglut1Cre/+; Sun1-GFPfl/+ , VgatCre/+; Sun1-GFPfl/+ , Olig2Cre/+; Sun1-GFPfl/+ animals" - this is 6 genotypes, 4 (?) cell types, and an unknown number of biological replicates.
> snRNAseq - additional concerns about the power of these experiments, as only n=2 animals were sequenced per group, drastically decreasing the validity of differential gene expression testing. This is not current best practice for such experiments

> bulkRNAseq - additional concerns about the power of these experiments, each sample contains a mixture of a single male and a single female animal (ie. sex was not considered as a biological variable). The authors should bioinformatically disentangle the representation of each sex, and determine if the responses reported are driven by a single sex.
> MERFISH spatial data are not deposited on the GEO, and as such unavailable for review. An estimation from Extended Data Fig 4 is that an additional 8 samples are missing from the GEO

3. effects of peripheral hormones - peripheral cortisol effects on astrocytes following visual experience is an interesting mechanism. Can the authors comment about other peripheral hormones that are affected by visual experience - e.g. melatonin, estrogen, serotonin, dopamine - all of which have receptors expressed by astrocytes. Why was cortisol chosen above other hormones?

4. staining of ECM components of perineuronal nets (PNNs) - WFA has been used to label PNNs, which is fairly standard in the field. However, the authors should be careful to consider that WFA can also bind heparin, n-glyocoproteins, and non-sulfated tetrasaccharides. In light of this, how would non-PNN staining of these components alter their interpretation of these data?
This concern is somewhat mitigated by the measurement of specific PNN proteins such as aggregan (see Extended Data Fig 12d)

5. the extension of interpretation from visual critical period to developmental and early life stressors leading to neurological/neuropsychiatric dysfunction is intriguing, but perhaps a little premature. I would suggest toning down these statements, or more clearly stating these are hypotheses and not a direct interpretation of the data shown in the mouse visual plasticity critical period experiments presented here.

6. lack of exclusion criteria for sequencing methods - methods for SHARE-seq describe 40 samples (5 ages, n=4 mice per timepoint and condition (dark rear, and normal)), however the figures only report 38 samples. What were the exclusion criteria used here? (these are not described in the methods)

(Remarks on code availability)
custom scripts noted at line 1063 are provided in a github repo that is incomplete or not accessible.
ATACseq code appears fine as these are standard ENCODE pipelines. I did not run the raw data generated here through the pipeline to determine if the same results could be obtained.

additional concerns over the missing dozens of samples of sequencing data (see comments to authors) mean that even in the presence of such code, no output files can be validated.

Referee #4

(Remarks to the Author)
Gegenhuber and colleagues set out to answer a fundamental question in developmental neuroscience, “What restrains early-life plasticity?” They examine the role of glucocorticoid receptor (GR) signalling in cortical astrocytes during critical periods of neural development and plasticity. Previous work has implicated astrocytes as key players in the closure of developmental windows, but this study stands out due to the detailed examination of the influence of GR signalling on astrocytes and the consequences for nearby neuronal and glial counterparts.
Here, the authors rear animals in standard lighting conditions (12h light:12h dark), or constant darkness across several developmental milestones in order to assess the impact of visual experience on gene expression. They use multiple approaches including, single-cell transcriptomics, chromatin accessibility sequencing, as well as morphological evaluation of astrocytes, immunohistochemistry and electrophysiological recordings to make a compelling case for GCR signaling in regulating critical period closure by promoting development of the extracellular matrix.

The primary observation is that GR deletion in astrocytes in the visual cortex resulted in an immature-like phenotype as assessed using transcriptomics, morphological assays, and measures of PNN development. The data presented strongly suggest that GR signalling is a master regulator of astrocyte development and that disruption of this process has important downstream consequences on neural circuit development.
While astrocytic GR signalling and its impact on astrocyte morphology, gliotransmission, neuronal activity, and behaviour has been studied within the context of stress this manuscript is the first to causally link GR signalling in astrocytes and to critical period closure during development.

Technically, the authors employed a tour de force approach using a robust array of experiments to describe the role of GR signaling in astrocytes and they should be commended for their efforts on this front. Nevertheless, certain connections remain somewhat speculative, and additional data and experiments would strengthen the overall framework of the study.

Major
1) The authors make causal links between sensory experience and cellular plasticity in the visual cortex using Light:Dark or Dark:Dark rearing. To be certain this is due to experience dependent plasticity within the visual cortex there are several important confounding factors that must be addressed, as this may not be a direct consequence of visual experience in V1 astrocytes. Specifically, how can the authors be certain that the changes described are induced by visual experience and not the result of global changes in the brain that are entrained by light via subcortical brain structures. It is critical for the authors to determine whether this is a V1 -specific phenotype, or more likely, a brain-wide phenomenon of astrocyte development entrained by maturation of the glucocorticoid system which occurs in parallel to eye opening and visual cortex

development.

To address this the authors need would need to determine how SCN, or other similarly light-entrained brain regions, regulate glucocorticoid levels and how this impacts astrocyte development specifically in visual cortex compared to other structures. SCN receives light cues and is entrained by light independently of the visual cortex. The authors appear to make links between activity in the visual cortex and blood glucocorticoids, but this observation most likely stems from subcortical brain regions.

2) The authors are aware of the stress hyposensitive period and the fact that that the brain is essentially non-responsive to glucocorticoids until around P15, as they reference Sapolsky and Meaney 1986. Despite this, there is important astrocytic development before and after this time window with astrocyte numbers and gene expression reaching mature levels by roughly P14 (Rurak et al. Cell Reports 2022). How do the data presented here, showing in immature phenotype, align with these previous reports?

3) The experiment to determine whether visual experience recruits GR to astrocyte chromatin to regulate gene expression (Fig. 2D-F and supplementary figures), including astrocyte-specific deletion of GR in V1 with analysis at P14 is formidable, one cannot rule out that the changes in GR related gene expression are simply due to differential glucocorticoid levels, rather than visual experience per se. Further explanation is required.

4) The validation of the viral approach to delete GR from astrocytes is impressive, it is very rare to observe a 100% KD efficiency. In the representative extended data figure Fig 7C, it is striking that there does not appear to be any GR expression outside of the nuclear area. As GR translocates from the cytosol to the nucleus upon binding to its ligand, these representative images suggest that the entirety of GR is ligand bound (only found in nuclear compartment) which would be indicative of an important stress response (very high CORT levels). Can the authors comment further on this observation?

5) Additionally, low magnification images to demonstrate specific targeting of visual cortex and viral spread would be important additions to the manuscript for future reproducibility.

6) The authors should determine whether all astrocytes express GR or whether it exists only in a subset of cells. Other work suggests enrichment of GR in specific populations of astrocytes (Chung et al. Nature 2025). Indeed, other stress-related receptors e.g. oxytocin receptors are only expressed in a subset of cells (Wahis et al. Nat Neurosci 2021), and astrocytes are highly diverse within cortical structures (Bayraktar et al. Nat Neurosci 2020). The authors already have the single-cell data available so this would be highly interesting and valuable insight to add. If the authors do observe this diversity within their region of interest, does this contribute to the higher variability in astrocyte morphology in controls (GR+ and potentially GR-) compared to GR-KO mice (which they show results in a 100% KO efficiency)?

7) In figure 4C-D, the authors set out to dissect the relationship between circadian fluctuations in glucocorticoids and astrocytic regulation of PNNs. To do this they used their GR KO approach, which is a permanent manipulation, but it's not clear whether this specific experiment addresses their question. To determine whether "daily astrocyte GR activation might be required to sustain PNNs in the mature brain," the authors should manipulate CORT levels. Following this initial characterisation of daily fluctuations on CORT on PNNs, the deletion of GR in this instance would add causality.

8) Is it surprising that PNN maturity had no effect on IPSCs or EPSCs onto Pyramidal neurons? From my understanding PNNs are highly implicated in synaptic plasticity. As such, a synaptic plasticity manipulation, rather than investigating basal transmission, might be more appropriate.

9) Regarding these electrophysiological manipulations, are the neurons recorded adjacent to, or within the domain of GR KO astrocytes or not?

10) In the introduction, the authors indicate that, "the specific mechanisms by which glia promote critical period closure have not been identified." This is not entirely accurate, as an earlier study (cited by the authors) went into considerable detail showing a potential role for astrocyte connexins and regulation of the extracellular matrix through MMP-9 (Ribot et al, Science, 2021). How do the current findings fit with these previous observations, given that the GCR and connexins have been linked in the stress literature.

Minor
1) Please verify the statistics on Figure 2C (P10 timepoint) as it does not appear to be significant.

2) In the final sentence of the results the authors state that "As dark-rearing has been shown to delay the physiological, transcriptional, and structural maturation of V1, we conclude that eliminating astrocyte GR function likewise shifts V1 neurons to an immature state." How can the authors definitely state that this remains immature and is not simply delayed as they previous literature they cite has demonstrated?

3) The authors state that "these data indicate light exposure around eye-opening induces systemic CORT production". Based on their data in figure 2C, this appears to be an overstatement. While light-exposure enhanced systemic CORT production around eye-opening, CORT was still elevated at this time point, compared to P10, and both conditions had identical blood CORT levels by P21.

4) The authors discuss the relevance of targeting GR in astrocytes in early life stress. A recent publication on this subject

has been published showing how deletion of GR in astrocytes rescues the effects of early life stress (Depaauw-Holt et al. Nat Comms 2025) and should be discussed in the context of this work.

5) Can the authors comment on the trends observed in Figure 1C. Why do experience-dependent genes decrease in glial cell types following P28, while neuronal cells continue to increase. Is this not surprising? Shouldn't all cell types scale similarly as glial activity has been shown to be critical for neuronal plasticity across development and in adult mice? One interpretation of these data is that over time the difference between DD and LD rearing narrows suggesting a slowed development that might reach equivalence with time.

6) It doesn't appear that sex as a biological variable has been addressed. Although the authors used both sexes, considering recent data (Depaauw-Holt et al. Nat Comms 2025) showing dramatic sex differences in astrocytic responses to stress, there could be interesting findings in their datasets. This is not essential but appears to be a missed opportunity.

(Remarks on code availability)

Referee #5

(Remarks to the Author)
I co-reviewed this manuscript with one of the reviewers who provided the listed reports.

(Remarks on code availability)
Couldn't access code on this link.

Version 1:

Reviewer comments:

Referee #1

(Remarks to the Author)
Summary:

In the revised manuscript, the addition of monocular deprivation experiments demonstrating reopening of ocular dominance plasticity following astrocyte-specific GR deletion in adults represents an important functional advance. This experiment directly addresses a central concern in the initial review and significantly improves the manuscript's mechanistic depth. The additional analyses of PV interneuron density, PNN prevalence, paired-pulse measurements, and earlier validation of GR-cKO further enhance the study's rigor and completeness.
Overall, this remains an exceptionally rich and technically sophisticated dataset of broad interest to developmental neurobiology and glial biology. The integration of epigenomic profiling with circuit-level manipulation is a major strength.
At the same time, several conceptual and interpretational issues remain that should be addressed before publication. These largely concern the strength of causal claims and the scope of certain conclusions. The additional data support a model in which astrocyte GR signaling contributes to ECM maturation and limits plasticity; however, some statements continue to extend beyond what has been directly demonstrated.
The manuscript would benefit from further refining the language to ensure that the conclusions align precisely with the experimental evidence.

Remaining Concerns:

1- Causality of corticosterone–GR signaling: The manuscript continues to frame the light-induced corticosterone surge as a driver of GR activation in astrocytes and downstream maturation programs. While the dark-rearing plus CORT supplementation experiments support a role for glucocorticoids in ECM maturation, they do not directly demonstrate that endogenous corticosterone is necessary for astrocyte GR chromatin recruitment, nor that CORT is sufficient to induce GR genomic binding in vivo.
Thus, the causal step linking light to systemic CORT and, in turn, to astrocyte GR chromatin recruitment remains inferred rather than demonstrated.

I recommend revising the language throughout to clarify that:
- Light enhances systemic corticosterone production, which is consistent with GR activation in astrocytes.
- The data support, but do not establish, the necessity and sufficiency of endogenous CORT for astrocyte GR genomic recruitment.
Framing this step as a supported model rather than a demonstrated causal chain would more accurately reflect the evidence presented. Otherwise, additional experiments are needed to support this claim.

2- Critical period closure versus restriction of plasticity: The newly added adult monocular deprivation experiments convincingly demonstrate that astrocyte GR deletion reopens ocular dominance plasticity in adulthood. This is an important finding. However, the manuscript continues to state, in several places (including the Abstract), that astrocyte GR signaling "drives critical period closure." The developmental closure of the critical period itself was not directly tested (e.g., through developmental MD paradigms across the CP window). Instead, the data show that GR signaling promotes ECM/PNN

maturation and constrains plasticity, and that its removal permits plasticity in adults.

I suggest modifying the language from:
“promotes critical period closure.”
to
“promotes ECM maturation and limits plasticity,” or
“contributes to the restriction of plasticity associated with critical period closure.”
This distinction is important and will enhance the manuscript’s precision.

3. Human data and evolutionary conservation
Human analyses show compelling cross-species concordance in motif accessibility and gene program overlap. However, the data remain correlative. There is no functional manipulation in human tissue (which I agree is not trivial), demonstrating that GR drives astrocyte maturation programs in an analogous manner.
Statements referring to an “evolutionarily conserved GR-driven maturation program” should therefore be tempered to reflect concordance or conservation at the level of chromatin accessibility and gene overlap, rather than functional conservation of mechanism.

4. Interpretation of neuronal transcriptional effects: The snRNA-seq dataset (n = 2 animals per condition) provides suggestive evidence that astrocyte GR-cKO induces neuronal transcriptional signatures reminiscent of those observed in dark-reared animals. However, given the limited sample size and absence of functional neuronal measurements at the same developmental time point, claims regarding astrocyte-dependent suppression of neuronal maturation programs should be framed cautiously.
A brief acknowledgment of these limitations in the Results or Discussion is needed.

5. Broader implications for stress and psychiatric disease: The Discussion and Abstract continue to extend the findings toward early-life stress and psychiatric vulnerability. While these are intriguing and plausible hypotheses, they are not experimentally tested here.
Maintaining explicit hypothesis language (“may,” “could,” “raises the possibility”) and avoiding definitive phrasing in the Abstract would improve conceptual balance.

Minor Clarifications
- Where GR genomic recruitment is described as astrocyte-selective, it would be helpful to briefly acknowledge potential technical differences in cell-type isolation or detection sensitivity that may influence peak counts.
- Ensure consistent use of causal language (e.g., “supports a model in which” rather than “reveals” or “demonstrates”) when referring to multi-step mechanistic inferences.

(Remarks on code availability)

Referee #3

(Remarks to the Author)
The authors have addressed all my original concerns.

(Remarks on code availability)

Referee #4

(Remarks to the Author)
The revised manuscript by Gegenhuber and colleagues has been significantly strengthened following input from all three reviewers. The authors have performed a commendable amount of experimental work to address all core issues raised by the reviewers and made important text edits to improve clarity of the manuscript.

In the current form, this manuscript represents an important addition to the field, meeting the high standards expected from an article published in Nature. I wish to congratulate the authors on this work and look forward to seeing the manuscript in print.

(Remarks on code availability)

Open Access This Peer Review File is licensed under a Creative Commons Attribution 4.0 International License, which permits use, sharing, adaptation, distribution and reproduction in any medium or format, as long as you give appropriate credit to the original author(s) and the source, provide a link to the Creative Commons license, and indicate if changes were made.
In cases where reviewers are anonymous, credit should be given to 'Anonymous Referee' and the source.
The images or other third party material in this Peer Review File are included in the article's Creative Commons license, unless indicated otherwise in a credit line to the material. If material is not included in the article's Creative Commons license and your intended use is not permitted by statutory regulation or exceeds the permitted use, you will need to obtain permission directly from the copyright holder.
To view a copy of this license, visit https://creativecommons.org/licenses/by/4.0/

Referee #1 (Remarks to the Author):

Summary

This is a well-designed and rigorously executed study that addresses a longstanding question in developmental neurobiology: how systemic signals interface with local cortical circuits to regulate critical period plasticity. By combining transcriptomic and epigenomic profiling (SHARE-seq, MERFISH, CUT&RUN, ATAC-seq, RNA-seq, snRNA-seq) with astrocyte-specific manipulations, the authors identify glucocorticoid receptor (GR) signaling in astrocytes as a driver of astrocyte maturation, extracellular matrix formation, and restriction of plasticity.

The dataset is exceptionally rich, technically sophisticated, and of broad value to the neuroscience community. The findings will interest not only developmental and glial biologists but also those working on endocrinology, stress, and psychiatric disease. The conceptual advance—linking systemic corticosterone signaling to astrocyte-driven closure of cortical plasticity—is novel and compelling.

We are grateful that this reviewer recognizes the many strengths in our study. And we thank the reviewer for their many helpful suggestions.

At the same time, the manuscript makes causal claims that go beyond the presented data. In particular, the relationship between corticosterone surge and astrocyte GR activation is correlative, and the functional demonstration that GR restricts ocular dominance plasticity (ODP) is lacking. While the enthusiasm for the dataset and analysis is high, additional experiments would be necessary to fully support the central claims.

We have addressed this major concern by conducting an experiment that measures the effect of GR loss in astrocytes on ocular dominance plasticity (see below). We have also addressed the concern raised by the reviewer that our findings are correlative (see below).

Major Concerns

Causality of corticosterone–GR signaling: The light-induced rise in corticosterone at eye opening is a key link in the proposed mechanism, but necessity and sufficiency are not tested. Adrenalectomy, pharmacological CORT supplementation, or GR antagonism would directly test this causal link.

We appreciate the reviewer's point that we have not directly shown that the rise in CORT is responsible for the activation of GR in astrocytes. However, we demonstrate that upon eye-opening, there is a surge in CORT, and GR is activated in astrocytes, and that when we dark-rear mice, which suppresses this surge, GR is not activated. It has been established for at least 30 years that the primary mode of GR activation is through glucocorticoid binding. So, it stands to reason that the increase in circulating CORT activates GR in astrocytes. To partially address the reviewer's concern, we asked if pharmacological CORT supplementation to dark-reared (DR) animals around eye-opening (P14) restores perineuronal nets (PNNs) to levels observed in normal-reared (NR) animals. Notably, pharmacological CORT supplementation to DR animals elevates PNNs and partially restored them to levels observed in NR mice. We conclude that CORT promotes PNN assembly, as previously suggested by our astrocyte GR-KO experiments in neonatal and adult animals (Fig. 4a-d). We have included these new findings in Extended Data. Fig. 16 and added the corresponding text to the Results section on page 7 of the manuscript.

We further point out that the experiments performed in our manuscript involving AAV-mediated genetic deletion of GR specifically in V1 astrocytes are not simply correlative and do test the necessity of GR signaling for the reported phenotypes. To the best of our knowledge, this is the most rigorous approach for testing the necessity of CORT-GR signaling, as it allows us to block this pathway within a specific cell type and brain region without incurring the widespread secondary and often deleterious effects that are known to occur upon systemic manipulation of CORT levels.

We note that in adult mice the adrenalectomy experiments suggested by the reviewer, in addition to eliminating CORT, cause weight loss (Green et al. 1992 Endocrinology), impaired physical activity (Veldhuis et al. 1982 Horm Behav), impaired learning and memory (Vaher et al. 1994 Brain Res), reduced glucose and insulin levels (Van Lan et al. 1974 Endocrinology), reduced epinephrine levels (Pohorecky & Wurtman 1971 Pharmacol Rev), reduced aldosterone levels (Stanton et al. 1985 J Clin Invest), elevated cytokine levels (Goujon et al. 1996 Brain Res Mol Brain Res), and elevated renin levels (Welch et al. 1985 Am J Physiol) among other phenotypes, making it challenging to assign the causality of this manipulation to the loss of CORT specifically rather than another affected signaling pathway. Moreover, while blocking light-induced CORT release may be feasible in adult mice via virally-mediated ablation of intrinsically photosensitive retinal ganglion cells (ipRGCs) that project to the suprachiasmatic nucleus (SCN) (Meng et al. 2023 Cell), complete SCN ablation (Ishida et al. 2005 Cell Metab) or severing of the retinohypothalamic tract (Moore and Klein 1974 Brain Res), we could not find any papers that have applied these methods to neonatal (<P14) mice, as the required surgeries cannot be performed at a young age. For these reasons we have

chosen to not perform the adrenalectomy experiments proposed by the reviewer, and hope the reviewer will accept that such an analysis is well beyond the scope of the current study.

Direct demonstration of critical period closure: The study infers effects on ODP from dark rearing and molecular markers. However, ODP requires competitive manipulation (e.g., monocular deprivation) with physiological or behavioral readouts. Without this, the claim that astrocyte GR "restricts plasticity" is premature.

We agree that this experiment is essential to the primary conclusions of the manuscript and thus have investigated the effects of astrocyte GR signaling on ocular dominance plasticity (ODP) in collaboration with the laboratory of Dr. Chinfei Chen. We delivered astrocyte-selective AAVs expressing Cre or catalytically-inactive ΔCre into V1 in adult (P35-P40) $GR^{fl/fl}$ mice and, after waiting three weeks for viral transduction, recorded visual responses from binocular V1 using multi-electrode arrays following normal rearing (NR) or 3-4 days of monocular deprivation (MD). We found that eliminating GR in V1 astrocytes restores ODP in adult mice, as indicated by an increase in the relative strength of visual responses from the non-deprived eye following MD without affecting responses in NR animals. These findings are now reported in Figure 5 and Extended Data Fig. 19 and described on page 8 of the revised manuscript.

Specificity of astrocyte GR action: While GR binding appears enriched in astrocytes, GR is broadly expressed. Stronger evidence is needed to exclude indirect circuit-level effects, especially since many of the neuronal changes could be secondary.

We appreciate the reviewer's comment regarding how astrocyte GR signaling influences neuronal phenotypes. Although GR is indeed broadly expressed across most cortical cell types (Extended Data Fig. 10a-b), our viral approach uses the combination of the GfaABC1D promoter and a miRNA targeting cassette (Gleichman et al. 2023 Nat Commun) to effectively restrict GR deletion specifically to astrocytes (Extended Data Fig. 8h-k). Thus, phenotypes that emerge in neighboring neurons (PNNs, synapse numbers, gene expression, ODP) are secondary to the loss of the astrocyte GR signaling pathway. We have clarified this point in our revised manuscript on page 6.

Circuit-level function: Altered PNNs and bouton densities are observed, but PV-evoked IPSCs are unchanged. Presynaptic measures (e.g., paired-pulse ratio) would more directly assess synaptic changes. The mechanistic link between astrocytic ECM regulation and PV function requires clarification.

We appreciate the reviewer's comment on examining the effects of astrocyte GR deletion on PV-mediated presynaptic electrophysiology. In the revised manuscript, we have

measured paired-pulse ratio of PV-evoked currents onto L5 pyramidal neurons using 5 stimulation pulses (reported in Extended Data Fig. 17k) and observed no effect of astrocyte GR-KO on this form of short-term plasticity. We have included a description of this result in the main text of our revised paper on page 7.

More broadly, the relationship between PNNs and neural circuit physiology remains poorly understood, as effects likely depend on brain region, method of PNN elimination, and timing of recordings following PNN removal. However, our finding of reduced PNNs in the absence of changes in miniature excitatory or inhibitory postsynaptic currents (mEPSCs or mIPSCs, respectively) is consistent with a recent comprehensive study examining the effects of chondroitinase ABC (ChABC) treatment on electrophysiological properties of V1 layer 4 (L4) pyramidal neurons (PyNs) and PV+ interneurons (Faini et al. 2018 Elife). In this study, the authors report that short-term enzymatic removal of PNNs in vivo – a manipulation previously shown to re-open ODP in adult V1 (Pizzorusso et al. 2002 Science) – does not alter mEPSCs or mIPSCs onto PyNs (Faini et al. 2018 Elife Figure 2—figure supplement 4) nor intrinsic firing properties of PV+ interneurons (Faini et al. 2018 Elife Figure 2—figure supplement 2). Rather the primary direct consequence of PNN removal is an increase in thalamocortical synapses onto PV+ interneurons that requires sensory input (Faini et al. 2018 Elife FIgure 2). These findings suggest that PNNs restrict the turnover of thalamocortical synapses onto PV+ interneurons, such that combining PNN removal with monocular deprivation enables synaptic plasticity onto these cells. We now cite these findings in our revised manuscript on page 7.

Human data interpretation: The human extension is valuable but correlative. The claim of conserved GR-driven astrocyte maturation should be tempered unless supported by functional human tissue or organoid data.

We have tempered our conclusions on the finding of conserved GR binding sites and target genes across human astrocyte development in the absence of functional data on page 6 of our revised manuscript.

Speculation on stress and psychiatric disease: The discussion extends to early-life stress and psychiatric vulnerability. While intriguing, these claims are not experimentally supported in the present work and should be phrased more cautiously.

We have re-written the final paragraph of the Discussion in a more cautious manner, softening our predictions on how early-life stress may influence V1 plasticity.

Detailed Comments:

Figure 1:
- The patterns of changes in experience-dependent gene counts are remarkably unique in different cell types: the peak (P28) in non-neuronal cell types (astros, oligos, and microglia) is distinct from several types of excitatory neurons (P21 and P35) and PV interneurons (P35). This finding seems to fit well with the authors' hypothesis that "astrocytes…might be uniquely poised to take up peripheral hormones such as glucocorticoids in response to visual experience" and would benefit from further discussion and interpretation in the main text.

We agree that this is an interesting finding that would benefit from additional discussion in the text. We have now analyzed the genes altered by visual experience in glial cell types – oligodendrocytes, microglia, and astrocytes – at P28 and found strong enrichment for genes involved in cell type-specific maturational processes occurring at this stage in development.

In oligodendrocytes, visual experience at P28 induces a broad gene program that promotes maturation and myelination, including Olig1, Olig2, Sox10, Myrf, Mbp, Plp1, Mog, Mobp, Cnp, Ugt8a, Fa2h, Gal3st1, Mal, Ermn, and Cldn11, among others. Notably, this timing coincides with the onset of oligodendrocyte-axon contact and myelination in V1 (McGee et al. 2005 Science) and aligns with the well-established role of neuronal activity in promoting myelination (Bonetto et al. 2021 Science).

Likewise, in microglia, visual experience induces genes characteristic of the transition from a developmental phagocytic state to a mature surveillance state, including Tgfb1, Tgfbr1 and downstream TGF-b signaling targets such as Tmem119, Hexb, Fcrls, P2ry12, Golm1, Mertk, among others (Butovsky et al. 2013 Nat Neurosci; Spittau et al. 2020 Trends Immunol), as well as Csf1r (Kierdorf et al. 2013 Nat Neurosci). Consistent with this observation, dark-rearing is known to occlude the transition of V1 microglia from a phagocytic state to a mature state (Tremblay et al. 2010 PLoS Biol). We also observed experience-induced expression of metabolic genes in microglia, including P2ry12 and Entpd1/CD39, which mediate negative feedback control of neuronal activity (Badimon et al. 2020 Nature).

We have now included a brief interpretation of these findings in the Results on *page 2 of the revised manuscript.*

- The authors include very little comparative analysis of the p21 and p35 MERFISH data. Additional plots and/or text discussion of age-specific differences in MERFISH detection of experience-responsive genes would further strengthen the argument that diverse V1 cell types respond to visual input on distinct time scales.

We thank the reviewer for raising this point. We re-examined our MERFISH and SHARE-seq datasets to identify experience-responsive genes unique to the P21 and P35 time points across modalities. This analysis revealed a high number of experience-responsive genes that emerge at P35, particularly in excitatory neuron types. These include Cdh13 in L5PT neurons, which recruits PV-mediated perisomatic inhibition onto these cells (Jézéquel et al. 2025 Nat Commun) as well as several genes that encode synaptic adapter proteins that may regulate synapse stabilization late in development: Iqgap2 (L5PT, L6CT), Tspan13 (L5PT), and Mpp3 (L5PT). We also observe induction of synaptic plasticity genes Vgf (Alder et al. 2003 J Neurosci) and Syt12 (Kaeser-Woo et al. 2013 J Neurosci), which could support adult forms of LTP. We have now included these findings in the text and plotted the expression of these genes across datasets in Extended Data Figure 4 and described on page 2 of the revised manuscript.

Figure 2:
- The authors suggest that GR interacts with chromatin through tethering and cooperativity with NFIA and LHX transcription factors. However, the data supporting this claim is largely correlative and would be better supported by causal studies:
- Probe the impact of NFIA deletion on GR chromatin interactions by performing GR cut & run in astrocyte-specific NFIA-cKO vs control conditions.
- Probe the putative protein-protein interaction between NFIA and GR by performing co-immunoprecipitation in astrocytes or heterologous cell lines.

We appreciate the reviewer's comment regarding the proposed mechanism of cooperative binding between GR and NFIA and/or LHX2. To address this point, we have tempered our conclusions throughout this section to avoid overinterpretation. We acknowledge that these data are correlative. Although not central to the main conclusions of the manuscript, we nonetheless believe it is important to include this analysis.

As suggested by the reviewer, we proceeded to test whether GR can interact with NFIA or LHX2 in a heterologous system by co-transfecting FLAG- and HA-tagged constructs into HEK293T cells, followed by FLAG immunoprecipitations and Western blotting. We first validated the previously reported NFIA-LHX2 interaction (Cheng et al. 2023 Nature), observing that HA-LHX2 co-immunoprecipitates with FLAG-NFIA but not with a FLAG-NLS-GFP control. We then found that FLAG-GR, but not FLAG-NLS-GFP, co-immunoprecipitates with both HA-NFIA and HA-LHX2, independent of DNA, as these interactions persisted following benzonase treatment. These results indicate GR has the capacity to interact with either NFIA or LHX2.

We have modified this section of the Results on page 4 of our revised manuscript and included these findings in Extended Data Fig. 11.

Figure 3:
- Correlate RNAseq repressed/activated genes (RNAseq, 3c-d) and repressed/activated sites (ATACseq, 2f) in GR-KO vs CON

We examined whether GR-dependent ATAC sites preferentially localize near GR-dependent genes. We found that GR-activated ATAC sites are enriched near GR-activated genes, whereas GR-repressed ATAC sites are enriched near GR-repressed genes. We have included these results in Extended Data Fig. 13c-d and on pages 5-6 of our revised manuscript.

- Does 3g show only motifs that gained accessibility (vs. lost accessibility?)

Fig. 3g only shows the motifs that gain accessibility across human brain development.

- The discussion (lines 238-247) and figures pertaining to human astrocyte developmental gene programs, it is not clear whether differentially expressed (Fig 3i) and differentially accessible (Fig 3j) gene programs c1-c4 represent a common set of 4 programs or distinct programs identified through separate hierarchical clustering of snRNAseq and ATACseq data. Based on the methods (lines 1010-1028), it seems that the latter is true. This should be clarified in the text and figures.

The differentially expressed genes and differentially accessible ATAC sites are distinct programs identified through separate hierarchical clustering of snRNA-seq and snATAC-seq data. We have clarified this now in the Results section on page 6.

Figure 4:
- Since Acan is PV-specific (Fig S12d-e), how do the authors interpret the finding that an astrocyte-specific manipulation (GR-cKO) impacts Acan abundance? Perhaps other astrocyte-specific proteins (e.g., proteases) modulate its stability? If so, this point would be well-supported by direct manipulation of an astrocyte-specific ECM modulator (e.g., Mmp2) and readout of Acan abundance. This portion of the study would also be strengthened by immunohistochemical staining for other PNN components (e.g., Vcan or Tnc), which are directly changing expression in astrocytes.

As discussed below, the experiments proposed by the reviewer – while worthwhile - to be performed properly would require a detailed analysis that would be most appropriate for a separate paper.

The abundance of chondroitin sulfate proteoglycans (CSPGs) that constitute PNNs, such as Acan, depends on the balance between protein expression and degradation. We find that none of the known PNN-associated glycoproteins (Acan, Bcan, Ncan, Ptprz1/Phosphacan, Tnr/Tenascin-R, or Vcan V2) are regulated transcriptionally by GR in astrocytes (Supplementary Table 8), nor do they change expression in neurons following astrocyte GR-KO (Supplementary Table 10). However, a number of metalloprotease enzymes (Adamts5, Adamts9, Adamts12, Adamts16, Mmp2, Mmp14; shown in Extended Data Fig. 15c) increase expression in astrocytes upon GR-KO, and several of these enzymes have been shown to cleave PNN-associated proteoglycans in biochemical assays in vitro (ADAMTS5 cleavage of aggrecan: Tortorella et al. 2002 Matrix Biol; ADAMTS9 cleavage of versican: Martin et al. 2025 J Biol Chem; ADAMTS12 cleavage of neurocan: Fontanil et al. 2019 Cell Physiol Biochem; ADAMTS16 cleavage of aggrecan: Zeng et al. 2006 Biochim Biophys Acta; MMP2 cleavage of aggrecan: Fosang et al. 1992 J Biol Chem; MMP14 cleavage of aggrecan: Fosang et al. 1998 FEBS Lett; MMP14 cleavage of pro-MMP2: Sato et al. 1994 Nature). Thus, we predict that, after eye-opening, GR represses a family of secreted metalloproteases in astrocytes, enabling the condensation of PNNs on nearby PV+ interneurons. Notably, this model is consistent with the suppression of metalloproteases that is known to occur during astrocyte maturation (Dallérac et al. 2018 Nat Rev Neurosci – Figure 2). We now highlight this point on page 6 of our revised manuscript.

We agree with the reviewer's comment on the importance of characterizing the mechanism underlying astrocyte GR regulation of Acan/PNNs. However, to date, none of the six enzymes listed above have yet been shown to regulate PNNs in the brain. Therefore, characterizing the mechanism by which astrocyte GR signaling regulates PNNs would likely require a comprehensive shRNA or CRISPR genetic screen starting with these six metalloprotease candidates, which we feel is beyond the scope of the current study.

The reviewer suggests that we immunostain for Tn/Tenascin-C and the Vcan V0/V1 isoforms, as the genes that encode these proteins are regulated by GR (Extended Data Fig. 15c and see below). However, we feel that such an analysis will not particularly add to the current study given that Tn/Tenascin-C and Vcan V0/V1 are not components of PNNs (Deepa et al. 2006 J Biol Chem), and thus immunohistochemical staining for these proteins would likely not provide any additional information regarding the mechanism of GR-dependent PNN formation. Moreover, aggrecan is required for the assembly of other CSPGs (Bcan, Ncan, Phosphacan, Vcan V2) into PNNs (Rowlands et al. 2018 J Neurosci). As astrocyte GR-KO reduces PV-associated aggrecan (Extended Data Fig. 16n-o), we would expect a corresponding reduction in the somatic localization of these other CSPGs, regardless of their degradation status.

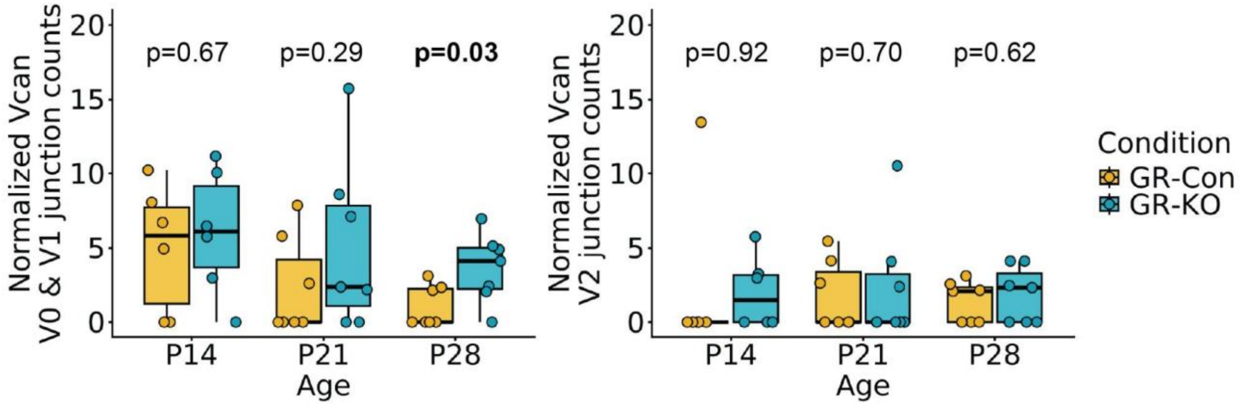

- GR-cKO reduces WFA intensity on a per-cell basis in both adolescents and adults (Fig. 4a- d, Fig. S12), but the authors do not directly test whether GR-cKO impacts PVI differentiation/maturation or survival. Mean PV intensity is used as a proxy (Fig. S12f-h); however, the findings of reduced PV intensity in adolescents but not in adults are not discussed in the text. This may be relevant to interpret the reduced Syt2+ synapse counts (i.e., are individual PVIs forming fewer Syt2+ synaptic inputs, or are there fewer PVIs?). We recommend PVI and PNN counting analyses, such as the number/density of PVIs and PNNs, and the proportion of PVIs with PNNs.

In response to the reviewer's suggestion, we quantified PVIs and PNNs at P35 and in adult mice. At both time points, we did not observe any change in the number of PVIs between astrocyte GR-KO and GR-Con animals. These results are consistent with prior reports showing that eliminating PNNs through ChABC treatment or Acan deletion (Yamada et al. 2015 Eur J Neurosci – Fig. 5; Rowlands et al. 2018 J Neurosci – Fig. 2) shifts PVIs to a low PV-expressing state without affecting PVI number. Together with our inhibitory synaptic staining data, these findings suggest that individual PVIs form fewer Syt2+ synaptic inputs onto pyramidal neurons following astrocyte GR KO.

We did not observe any change in the proportion of PVIs ensheathed by PNNs at P35 between astrocyte GR-KO and GR-Con animals. In adult mice, however, we observed small reductions in PNN+ PVIs in V1 L5 and L6.

These results have been incorporated into a revised Extended Data Fig. 16 and are discussed on page 7 of the revised manuscript.

- Is the intensity of light stimulation (1-4 mW/mm2) a good approximation of physiologic PV firing (Fig. 4g-h, S13f-k)? Please cite evidence.

The intensity of light stimulation (1-4 mW/mm2) used for evoking PV activity in our optogenetic stimulation experiments were selected based on previously published standards in the field for inducing PV fast-spiking activity and evoking small IPSCs on pyramidal neurons (Gunaydin et al. 2010 Nat Neurosci; Zhu et al. 2015 Nat Commun; Iaccarino et al. 2016 Nature; Yap et al. 2020 Nature). We now cite this evidence in our Methods on page 19 of the revised manuscript.

- Given that immunohistochemical analysis showed changes in presynaptic bouton density, would paired-pulse ratio be a better electrophysiological measure of presynaptic strength changes? As the authors point out, evoked IPSCs could be compensated by postsynaptic strengthening and are thus a less specific measure of the observed presynaptic changes.

As addressed above, we have measured paired-pulse ratio of PV-evoked currents onto L5 pyramidal neurons (reported in Extended Data Fig. 17k) and observed no effect of astrocyte GR-KO on this form of short-term plasticity. We have now mentioned this finding in the main text on page 7.

- The authors suggest that “astrocyte-dependent suppression of neuronal gene expression might be critical for circuit maturation and the closure of critical periods” (lines 310-312). This claim would be better supported by monocular deprivation experiments in animals during and post-critical period.

We agree with the reviewer and have performed this key experiment, as described above. We think the inclusion of this experiment is a major addition to our study that greatly enhances its significance.

- Based on snRNAseq at p21, the authors suggest that the effects of GR-cKO on V1 pyramidal neurons mimic the effect of dark-rearing. However, intracortical excitatory synapses are only tested at p35 and are unchanged at this later time point. Additional assays of V1 neuron maturation/functon(e.g., synaptic staining or electrophysiological recordings) at p21 would strengthen the authors’ suggestion that the neuronal effects of astrocyte-specific GR-cKO mimic dark-rearing.

To test the possibility that astrocyte GR-KO differentially affects excitatory synapse number at P21 vs. P35, we delivered astrocyte-specific Cre- or ΔCre-expressing AAVs into $GR^{fl/fl}$ animals at birth and performed immunofluorescent staining for Vglut1 and Homer1 at P21. As previously observed at P35, we observed no effect of astrocyte GR-KO number on the density of excitatory synapses at this earlier time point. We include

these findings in Extended Data Fig. 17c-d and to the Results section on page 7 of our revised manuscript.

In this regard, several studies suggest intracortical excitatory synapse number, measured either via electron microscopy or electrophysiology as a proxy (mEPSC frequency), is not affected by dark-rearing (Vrensen & de Groot 1974 Brain Res; Cragg 1975 Exp Neurol; Bakkum et al. 1991 J Neurosci Res; Desai et al. 2002 Nat Neurosci; Goel et al. 2006 Nat Neurosci; Goel & Lee 2007 J Neurosci; Larsen et al. 2014 Neuron; Han et al. 2017 Cell Rep).

Moreover, our new finding of ODP reopening with astrocyte GR-KO (Fig. 5) provides an additional line of functional evidence that eliminating this signaling pathway mimics the effects of dark-rearing, which prolongs the critical period for ODP across species (Mower 1991 Brain Res Dev Brain Res; Fagiolini et al. 1994 Vision Res; Gianfranceschi et al. 2003 PNAS).

- The authors show reduced Syt2+ inputs to L5 PyNs at P35. Could a similar phenomenon be present earlier in development (e.g., P21) and thereby underlie the astrocyte-dependent changes in L5IT gene expression at P21? Staining and e-phys analysis of PVI inputs to L5 neurons at an earlier age would be informative on this point.

We investigated whether astrocyte GR signaling influences Syt2+/PV or CB1R+/CCK inputs onto L5 neurons by immunofluorescent staining at P21. Eliminating astrocytic GR led to a reduction in the density of perisomatic Syt2+ puncta on L5 neurons at P21, matching our previous observations at P35, but did not influence CB1R+ density (Extended Data Fig. 17a-b). We have included these findings in the Results section on page 7 of our revised manuscript.

It is possible that the reduction in PVI inputs at this earlier age contributes to the effects of astrocyte GR deletion on L5IT gene expression. We interpret these results as evidence that eliminating astrocytic GR produces a more permissive ECM, which in turn signals to neurons to upregulate a broad set of developmental axonal outgrowth genes – including Gap43 (Bomze et al. 2001 Nat Neurosci), Robo1 (Hivert et al. 2002 Mol Cell Neurosci), Syt17 (Ruhl et al. 2019 Nat Commun), Epha3 (Gao et al. 1999 PNAS), and others. A similar mechanism has previously been demonstrated in the spinal cord, where enzymatic removal of chondroitin sulfate proteoglycans via ChABC promotes axonal outgrowth and induces GAP-43 expression (Bradbury et al. 2002 Nature).

Figure S7:

- The validation of GR-cKO is performed in p35, whereas C&R experiments are all in P14, and astrocyte morphology at p21. GR-cKO should be performed at the earliest time point (P14) at which the model is used.

We have repeated GR immunofluorescent staining in astrocyte GR-KO and astrocyte GR-control animals at P21 – the same time point that we used to characterize other phenotypes, including astrocyte morphology (Fig. 3a-b), astrocyte chromatin accessibility (Fig. 3f), neuronal gene expression (Fig. 4i-n), and now inhibitory and excitatory synapse density (Extended Data Fig. 17a-d). Consistent with our prior observation at P35, Cre-T2a-GFP+ cells predominantly (~97%) express Sox9 and do not express GR protein at P21, whereas essentially all ΔCre-T2a-GFP+ cells express nuclear GR. These findings are reported in Extended Data Fig. 8i, 8k of our revised manuscript. In addition, we performed GR CUT&RUN and RNA-seq on astrocyte GR-KO nuclei at P14 and observed loss of GR binding to DNA (Fig. 2e, Extended Data Fig. 9g-h) and down-regulation of GR-target genes at this timepoint (Fig. 3c, Supplementary Table 8) and note these points on pages 4 and 5, respectively.

Figure S8:
- How heterogeneous is the expression of Nr3c1/GR in astrocytes? This can be addressed through further analysis of snRNAseq data, as well as the imaging dataset presented in Figure S7c (quantify the proportion of Sox9+ cells labeled with GR).

To address the reviewer's question, we have added plots to Extended Data Fig. 8 demonstrating that ~100% of Sox9+ cells (astrocytes) co-express GR protein, thus indicating minimal heterogeneity in GR expression across V1 astrocytes. We did not perform this type of analysis on our snRNA-seq dataset due to the likelihood of technical dropout precluding an accurate estimation of Nr3c1/GR expression. We have included a brief mention of this finding on page 5 of the revised manuscript.

Figure S10:
- There is existing evidence that glucocorticoid signaling induces Zbtb16 upregulation in other cell types. Please see the recent paper The glucocorticoid receptor is affected by its target ZBTB16 in a dissociated manner (Galuh et al., 2025) and its cited studies.

We thank the reviewer for making us aware of the literature on ZBTB16. We have mentioned the connection between GR and Zbtb16 in our revised manuscript on page 6.

Minor concerns:

Figure S10: Please clarify in the text whether the GR binding sites analyzed in panel A were identified from the CUT&RUN experiments performed in this study or through motif-based predictive analysis.

The astrocyte GR binding sites in panel A were identified experimentally by CUT&RUN. This point is clarified in our revised manuscript on page 5.

Referee #2 (Remarks to the Author):

I co-reviewed this manuscript with one of the reviewers who provided the listed reports.

Referee #3 (Remarks to the Author):

Here Gegenhuber and colleagues present data suggesting that astrocyte GR signaling is integral for the modulation of extracellular matrix (ECM) components, and as a consequence modulate neuron network experience-dependent specification of the mouse primary visual cortex during postnatal development. There have been numerous studies over the years that implicate astrocytes in synaptic plasticity and modulation of the ECM - largely focusing on early live development (synapse formation/function) or in response to acute injury (ECM component changes). The concept of bringing these two together here is compelling, and the authors provide some compelling evidence for the role of astrocytes in mediating this specific visual cortical plasticity.
As would be expected from this group, the manuscript is well written, and presents a narrative that is easy to follow, and is compelling. I have a number of concerns however (see below) largely focused on a gross oversight of providing sequencing data (even in light of Editorial Checklist confirmation that it is available), and missing code for analysis.

We appreciate the careful review of our manuscript and thank the reviewer for their many helpful suggestions.

All of the missing code is now included in the revised manuscript. We apologize for this oversight.

While the validation experiments themselves look well completed, that they are based on these expansive datasets (from SHARE-seq, single nucleus RNAseq, CUT&RUN, bulk RNAseq, and MERSCOPE spatial transcriptomics) but only 6 of the more than 60 (estimated) samples are available is concerning.

The missing datasets have been included in the revised manuscript.

These, if presented and made available would elevate this manuscript from an interesting and important biological mechanism, to a toolset for the community to mine and further a great number of exciting future discoveries. Additional concerns such as a lack of QC metrics across most of these datasets (owing to their absence except for key figure panels integral to the narrative) dampen enthusiasm for the impact of these part of the overall study.

We agree that it is essential that all of this information is included and have done so in the revised manuscript. We have included panels on key QC metrics for SHARE-seq (Extended Data Fig. 1a-i), MERSCOPE (Extended Data Fig. 3a-d), CUT&RUN (Extended Data Fig. 8c-d, 10h), bulk RNA-seq (Extended Data Fig. 12b), ATAC-seq (Extended Data Fig. 13b, e-f), and snRNA-seq (Extended Data Fig. 18a-b).

Points for consideration:
1. model of developmental plasticity - how important is neural circuit maturation in light of synaptic and network plasticity in the adult brain? Are the developmental and plasticity mechanisms different, or do the authors think that these mechanisms are similarly used in these different contexts?

We thank the reviewer for highlighting this important point regarding plasticity mechanisms in the developing and adult brain. Fundamental forms of neuronal plasticity – structural, Hebbian, spike-timing-dependent plasticity (STDP), among others – are generally thought to be similar during the critical period and in adulthood; however, the capacity for such plasticity declines over development due to the emergence of cell-extrinsic structural barriers and cell-intrinsic molecular programs. For example, spine turnover (Grutzendler et al. 2002 Nature), axonal plasticity (LeVay et al. 1980 J Comp Neurol), capacity for long-term potentiation (LTP) (Kirkwood et al. 1995 Nature), capacity for long-term depression (LTD) (Dudek and Friedlander 1996 Neuron), cholinergic sensitivity (Morishita et al. 2010 Science), and the duration of NMDA-dependent excitatory postsynaptic currents (EPSCs) (Carmignoto and Vicini 1992 Science; Hestrin 1992 Nature), among other processes, all decline markedly between the developing and adult brain.

In our revised manuscript, we show that elimination of GR in astrocytes similarly disrupts PNNs in both the developing and adult brain and, in addition, restores juvenile-like ocular dominance plasticity in adulthood as shown in Fig. 5 of our revised manuscript. We have added a sentence to the Discussion to highlight this point on mechanisms of developmental and adult plasticity on page 9.

2. Data availability concerns - the authors state that they completed new SHARE-seq (multiome) on mouse V1, however the only uploaded and available datasets in GSE299347 at 6 (3KO, 3CTL) bulk ATACseq datasets on purified astrocytes. Can the authors confirm if the SHARE-seq data was re-analyzed from the earlier Regev lab study, or if the data availability statement needs to be corrected and this new data uploaded the GEO and reviewed? (note this would mean 38 individual samples are missing from the GEO repository).
A review of the Editorial Policy Checklist shows that both the data availability statement and mandated accession code boxes have been ticked, but this is incorrect.
The code availability statement box has also been checked, but the custom git repo link pulls a 404 error message.
Additional sequencing data not available but presented in the manuscript and described in the methods include:
> CUT&RUN (both 3T3 cells, and isolated CNS cells). It should be noted there is a large number of samples missing here, as CUT&RUN was perform on "GR, NFIA, and IgG CUT&RUN experiments on excitatory neurons, inhibitory neurons, or oligodendrocytes, each biological replicate consisted of P14 V1 tissue pooled from 3-5 Vglut1Cre/+; Sun1-GFPfl/+ , VgatCre/+; Sun1-GFPfl/+ , Olig2Cre/+; Sun1-GFPfl/+ animals" - this is 6 genotypes, 4 (?) cell types, and an unknown number of biological replicates.
> snRNAseq - additional concerns about the power of these experiments, as only n=2 animals were sequenced per group, drastically decreasing the validity of differential gene expression testing. This is not current best practice for such experiments
> bulkRNAseq - additional concerns about the power of these experiments, each sample contains a mixture of a single male and a single female animal (ie. sex was not considered as a biological variable). The authors should bioinformatically disentangle the representation of each sex, and determine if the responses reported are driven by a single sex.
> MERFISH spatial data are not deposited on the GEO, and as such unavailable for review. An estimation from Extended Data Fig 4 is that an additional 8 samples are missing from the GEO

We apologize for the delay in uploading the remaining datasets to GEO and updating the access to our GitHub page. The GitHub page with all code used to analyze the data is now accessible at:

https://github.com/brunogegenhuber/GR_gene_reg

All sequencing data are now available on GEO under the new SuperSeries GSE306265 (token = gfwfgaysxvgrhez). Individual SubSeries for each sequencing method are listed

*below. The MERFISH data cannot be uploaded to GEO because it is not sequencing data. We have made the MERFISH dataset publicly available at FigShare**:***

https://doi.org/10.6084/m9.figshare.29971903

GSE299347: V1 ATAC-seq
P21 astrocyte GR-KO ATAC-seq, 3 replicates
P21 astrocyte GR-Con ATAC-seq, 3 replicates

GSE306261: V1 and 3T3 cell CUT&RUN
P14 astrocyte GFP+ DR GR CUT&RUN, 3 replicates
P14 astrocyte GFP+ NR GR CUT&RUN, 3 replicates
P14 astrocyte GFP- DR GR CUT&RUN, 2 replicates
P14 astrocyte GFP- NR GR CUT&RUN, 2 replicates
P14 astrocyte GFP+ DR IgG CUT&RUN, 1 replicate
P14 astrocyte GFP+ NR IgG CUT&RUN, 1 replicate
P14 astrocyte GFP+ NR GR-Con GR CUT&RUN, 2 replicates
P14 astrocyte GFP+ NR GR-KO GR CUT&RUN, 2 replicates
P14 astrocyte GFP+ NR NFIA CUT&RUN, 3 replicates
P14 oligodendrocyte GFP+ NR GR CUT&RUN, 3 replicates
P14 oligodendrocyte GFP+ NR NFIA CUT&RUN, 3 replicates
P14 oligodendrocyte GFP+ NR IgG CUT&RUN, 1 replicate
P14 inhibitory neuron GFP+ NR GR CUT&RUN, 3 replicates
P14 inhibitory neuron GFP+ NR NFIA CUT&RUN, 3 replicates
P14 inhibitory neuron GFP+ NR IgG CUT&RUN, 1 replicate
P14 excitatory neuron GFP+ NR GR CUT&RUN, 3 replicates
P14 excitatory neuron GFP+ NR NFIA CUT&RUN, 3 replicates
P14 excitatory neuron GFP+ NR IgG CUT&RUN, 1 replicate
3T3 cells, 50K Veh GR CUT&RUN, 2 replicates
3T3 cells, 50K DEX GR CUT&RUN, 2 replicates
3T3 cells, 250K Veh GR CUT&RUN, 2 replicates
3T3 cells, 250K DEX GR CUT&RUN, 2 replicates
3T3 cells, 250K Veh IgG CUT&RUN, 1 replicate
3T3 cells, 250K DEX IgG CUT&RUN, 1 replicate

GSE306262: V1 RNA-seq
P14 DR GR-Con RNA-seq, 3 replicates
P14 NR GR-Con RNA-seq, 3 replicates
P14 DR GR-KO RNA-seq, 3 replicates
P14 NR GR-KO RNA-seq, 3 replicates

P21 DR GR-Con RNA-seq, 4 replicates
P21 NR GR-Con RNA-seq, 3 replicates
P21 DR GR-KO RNA-seq, 4 replicates
P21 NR GR-KO RNA-seq, 3 replicates
P28 DR GR-Con RNA-seq, 4 replicates
P28 NR GR-Con RNA-seq, 3 replicates
P28 DR GR-KO RNA-seq, 4 replicates
P28 NR GR-KO RNA-seq, 3 replicates

GSE306263: V1 snRNA-seq
P21 NR GR-KO snRNA-seq, 2 replicates
P21 NR GR-Con snRNA-seq, 2 replicates
P21 DR GR-KO snRNA-seq, 2 replicates
P21 DR GR-Con snRNA-seq, 2 replicates

GSE306582: V1 SHARE-seq
P7 DR, 4 replicates
P7 NR, 4 replicates
P14 DR, 4 replicates
P14 NR, 4 replicates
P21 DR, 4 replicates
P21 NR, 4 replicates
P28 DR, 4 replicates
P28 NR, 4 replicates
P35 DR, 4 replicates
P35 NR, 4 replicates

For the bulk RNA-seq experiments, we have no evidence that differences in gene expression between samples are driven by a single sex (see Figure below), based on expression levels of sex chromosome genes (Ddx3x, Ddx3y). We note that two animals needed to be pooled for each sample to achieve a sufficient number of GFP-labeled nuclei for RNA-seq, and thus we pooled 1 female and 1 male for this experiment. As outlined in our response to reviewer #4 below, we find similar induction of visual experience-dependent genes in astrocytes across females and males in our SHARE-seq dataset (Extended Data Fig. 9). Moreover, we observed similar circulating levels of CORT between sexes in our ELISA and LC-MS data (Extended Data Fig. 6). We also examined whether the astrocyte GR-dependent gene program, which we previously observed to be conserved across mouse brain regions (Fig. 3e) and in the developing human brain (Fig. 3h-k), differs by sex and found similar results between females and

males. These analyses are now incorporated into Extended Data Figs. 12 and 14, respectively.

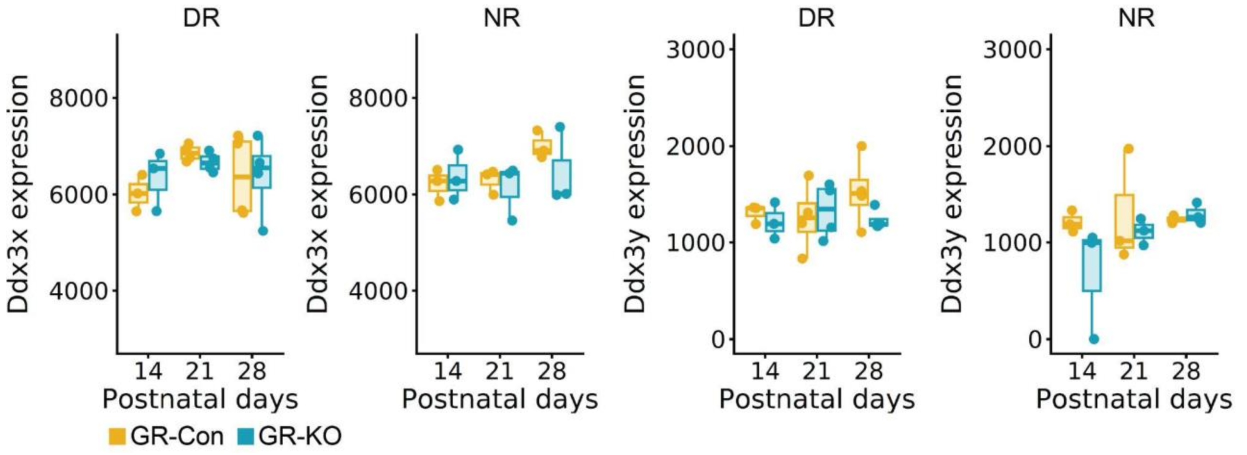

For the snRNA-seq experiments, we are likely under-powered to detect the complete repertoire of genes affected by eliminating GR in astrocytes. However, the genes that we do detect as differentially expressed are called at a pseudo-bulk (not single-cell) level and meet a standard FDR cutoff (Padj < 0.05). This point is clarified on page 26 of the revised manuscript.

3. effects of peripheral hormones - peripheral cortisol effects on astrocytes following visual experience is an interesting mechanism. Can the authors comment about other peripheral hormones that are affected by visual experience - e.g. melatonin, estrogen, serotonin, dopamine - all of which have receptors expressed by astrocytes. Why was cortisol chosen above other hormones?

Although we did not systematically assess whether other peripheral hormones are influenced by visual experience, we did measure thyroxine (T4) and report a subtle effect at P28 (Extended Data Fig. 6). We recognize that visual experience likely impacts the levels of additional peripheral hormones beyond glucocorticoids and, to address the reviewer's point, have made note of this on page 9 of our Discussion.

In our study, we focused on corticosterone (CORT), the primary glucocorticoid in most rodents, because our unbiased multiome analysis uncovered evidence for light-dependent changes in the activity of the glucocorticoid receptor – the nuclear hormone receptor for corticosterone – specifically in astrocytes.

4. staining of ECM components of perineuronal nets (PNNs) - WFA has been used to label PNNs, which is fairly standard in the field. However, the authors should be careful to consider that WFA can also bind heparin, n-glyocoproteins, and non-sulfated

tetrasaccharides. In light of this, how would non-PNN staining of these components alter their interpretation of these data?
This concern is somewhat mitigated by the measurement of specific PNN proteins such as aggregan (see Extended Data Fig 12d)

While WFA labeling has been a standard in the field for labeling PNNs (Härtig et al. 1992 Neuroreport), we take the reviewer's point that the specific molecular target for WFA is a non-sulfated tetrasaccharide of the O-O type, which are dimers of glucuronic acid and N-acetylgalactosamine [GlcA–GalNAc]$_2$ that comprise the chondroitin sulfate chains on PNN-associated glycoproteins aggrecan, brevican, neurocan, phosphacan, and versican V2. Beyond PNNs, select heparan sulfate proteoglycans (Hspg2/perlecan, Sdc1-4) contain a small number of non-sulfated O–O tetrasaccharides and could potentially be labeled by WFA. Notably, however, they contain an order of magnitude fewer chondroitin sulfate chains per core protein (Hspg2 = ~1-3 chains, aggrecan = ~100 chains), reducing the chances that they significantly contribute to the observed staining. One report has detected WFA recognition of heparin in a biochemical assay (Nadanaka et al. 2020 Biomolecules); however, given that heparin is largely absent in brain parenchyma, we do not believe that it would be labeled in our staining experiments. We have been unable to locate any reports of WFA labeling GalNAc N-glycoproteins in the brain. Given these considerations, and the measurement of the specific PNN protein aggrecan, we believe that we have met current accepted standards for PNN labeling.

5. the extension of interpretation from visual critical period to developmental and early life stressors leading to neurological/neuropsychiatric dysfunction is intriguing, but perhaps a little premature. I would suggest toning down these statements, or more clearly stating these are hypotheses and not a direct interpretation of the data shown in the mouse visual plasticity critical period experiments presented here.

We agree with this point and have accordingly re-written the final paragraph of the Discussion in a more cautious manner. We note, however, that reviewer #4 suggested we discuss the following paper on astrocyte GR signaling in early-life stress (Depaauw-Holt et al. 2025 Nat Commun).

6. lack of exclusion criteria for sequencing methods - methods for SHARE-seq describe 40 samples (5 ages, n=4 mice per timepoint and condition (dark rear, and normal)), however the figures only report 38 samples. What were the exclusion criteria used here? (these are not described in the methods)

We apologize for any confusion. There are 40 samples in the figures (Extended Data Fig. 1). No exclusion criteria were used.

Referee #3 (Remarks on code availability):

custom scripts noted at line 1063 are provided in a github repo that is incomplete or not accessible.
ATACseq code appears fine as these are standard ENCODE pipelines. I did not run the raw data generated here through the pipeline to determine if the same results could be obtained.

additional concerns over the missing dozens of samples of sequencing data (see comments to authors) mean that even in the presence of such code, no output files can be validated.

We apologize for the delay in uploading the remaining datasets to GEO and updating the access to our GitHub page. The GitHub page with all code used to analyze the data is now accessible at:

https://github.com/brunogegenhuber/GR_gene_reg

All sequencing and MERFISH data have been made available on GEO and FigShare, respectively, as indicated in our response above.

Referee #4 (Remarks to the Author):

Gegenhuber and colleagues set out to answer a fundamental question in developmental neuroscience, "What restrains early-life plasticity?" They examine the role of glucocorticoid receptor (GR) signalling in cortical astrocytes during critical periods of neural development and plasticity. Previous work has implicated astrocytes as key players in the closure of developmental windows, but this study stands out due to the detailed examination of the influence of GR signalling on astrocytes and the consequences for nearby neuronal and glial counterparts.
Here, the authors rear animals in standard lighting conditions (12h light:12h dark), or constant darkness across several developmental milestones in order to assess the impact of visual experience on gene expression. They use multiple approaches including, single-cell transcriptomics, chromatin accessibility sequencing, as well as morphological evaluation of astrocytes, immunohistochemistry and electrophysiological recordings to make a compelling case for GCR signaling in regulating critical period closure by promoting development of the extracellular matrix.

The primary observation is that GR deletion in astrocytes in the visual cortex resulted in an immature-like phenotype as assessed using transcriptomics, morphological assays,

and measures of PNN development. The data presented strongly suggest that GR signalling is a master regulator of astrocyte development and that disruption of this process has important downstream consequences on neural circuit development. While astrocytic GR signalling and its impact on astrocyte morphology, gliotransmission, neuronal activity, and behaviour has been studied within the context of stress this manuscript is the first to causally link GR signalling in astrocytes and to critical period closure during development.

Technically, the authors employed a tour de force approach using a robust array of experiments to describe the role of GR signaling in astrocytes and they should be commended for their efforts on this front. Nevertheless, certain connections remain somewhat speculative, and additional data and experiments would strengthen the overall framework of the study.

We thank the reviewer for their strong support of our study and for their many helpful suggestions.

Major
1) The authors make causal links between sensory experience and cellular plasticity in the visual cortex using Light:Dark or Dark:Dark rearing. To be certain this is due to experience dependent plasticity within the visual cortex there are several important confounding factors that must be addressed, as this may not be a direct consequence of visual experience in V1 astrocytes. Specifically, how can the authors be certain that the changes described are induced by visual experience and not the result of global changes in the brain that are entrained by light via subcortical brain structures. It is critical for the authors to determine whether this is a V1 -specific phenotype, or more likely, a brain-wide phenomenon of astrocyte development entrained by maturation of the glucocorticoid system which occurs in parallel to eye opening and visual cortex development.

To address this the authors need would need to determine how SCN, or other similarly light-entrained brain regions, regulate glucocorticoid levels and how this impacts astrocyte development specifically in visual cortex compared to other structures. SCN receives light cues and is entrained by light independently of the visual cortex. The authors appear to make links between activity in the visual cortex and blood glucocorticoids, but this observation most likely stems from subcortical brain regions.

We thank the reviewer for raising this point regarding how visual experience influences astrocyte development. To clarify, our model proposes that loss of light exposure during dark-rearing reduces circulating CORT levels. This reduction in circulating CORT levels

impairs GR genomic recruitment and downstream gene expression changes in V1 astrocytes. We did not intend to suggest that V1 neuronal activity modulates astrocyte GR binding or systemic glucocorticoid levels. Therefore, to address the reviewer's concern, we have modified the manuscript, so it is now clear that the mechanism by which light around eye-opening induces the increase in CORT has not yet been established but that it is likely due to activation of the SCN. In particular, we no longer use the term "visual experience" to describe the induction of CORT but instead describe the induction as due to "light" on pages 3-4 of our revised manuscript.

While our virus-mediated GR loss-of-function studies demonstrate that the reported V1 phenotypes are driven by a local loss of V1 astrocyte GR signaling, we believe that the effects of light deprivation on astrocyte GR signaling are not confined to V1 but may occur throughout the brain as a result of changes in systemic glucocorticoid levels. In support of this model, we show that the same GR-dependent gene program found in V1 astrocytes emerges in astrocytes of the motor cortex, prefrontal cortex, striatum, and thalamus at the time of eye-opening (P14) (Fig. 3e, Extended Data Fig. 12h-j).

We investigated whether it would be feasible to specifically block light-induced CORT release to assign causality of light deprivation to the subcortical pathway. While blocking release is technically feasible in adult mice via virally-mediated ablation of intrinsically photosensitive retinal ganglion cells (ipRGCs) that project to the suprachiasmatic nucleus (SCN) (Meng et al. 2023 Cell), complete SCN ablation (Ishida et al. 2005 Cell Metab) or severing the retinohypothalamic tract (Moore and Klein 1974 Brain Res), we were unable to find any studies that have applied these methods in neonatal (<P14) mice, as the required surgeries cannot be performed at this age.

2) The authors are aware of the stress hyposensitive period and the fact that the brain is essentially non-responsive to glucocorticoids until around P15, as they reference Sapolsky and Meaney 1986. Despite this, there is important astrocytic development before and after this time window with astrocyte numbers and gene expression reaching mature levels by roughly P14 (Rurak et al. Cell Reports 2022). How do the data presented here, showing in immature phenotype, align with these previous reports?

We believe our data align well with previous reports on the developmental timing of astrocyte maturation. Astrocyte development proceeds through three distinct phases: differentiation (late embryonic), proliferation (late embryonic to the first postnatal week), and maturation (second to third postnatal week). While the gene regulatory mechanisms underlying the first two phases are well-characterized (Deneen et al. 2006 Neuron; Kang et al. 2012 Neuron), transcription factors that coordinate astrocyte maturation have

remained elusive (as noted in Lattke et al. 2021 Nat Commun). We speculate that this gap in knowledge may stem from early studies that relied heavily on astrocyte culture systems, which lack the full cellular diversity and signaling environment of the intact brain.

Prior rodent studies have reported a sharp induction of mature astrocyte marker genes at ~P14 (Cahoy et al. 2008 J Neurosci; Rurak et al. 2022 Cell Rep). Subsequently, between P14 and P28, astrocytes undergo critical cellular changes that define their mature identity, including volume expansion (Stogsdill et al. 2017 Nature), process elaboration (Morel et al. 2014 J Neurosci), and territory refinement (Bushong et al. 2004 Int J Dev Neurosci). Importantly, our data show that P14 coincides with the rise in circulating CORT levels (Fig. 2c), recruitment of GR to chromatin in astrocytes (Fig. 2d), and the onset of GR-dependent gene expression (Fig. 3c). Furthermore, ablation of GR in astrocytes occludes both the downstream transcriptional and morphological maturation of these cells (Fig. 3a-d) and now reproduces the effect of immature astrocytes on neuronal plasticity (Fig. 5 and Ribot et al. 2021 Science).

Together, these findings support a model in which, around P14 – the end of the stress hyporesponsive period in mice – GR is activated in astrocytes for the first time, thus triggering gene expression programs that drive their morphological maturation over the subsequent 1-2 weeks. To address the reviewer's point, we have added this description to the revised manuscript on page 9.

3) The experiment to determine whether visual experience recruits GR to astrocyte chromatin to regulate gene expression (Fig. 2D-F and supplementary figures), including astrocyte-specific deletion of GR in V1 with analysis at P14 is formidable, one cannot rule out that the changes in GR related gene expression are simply due to differential glucocorticoid levels, rather than visual experience per se. Further explanation is required.

We thank the reviewer for raising this point. As stated above, we believe that GR binding and downstream gene expression changes are driven by differential circulating CORT levels that arise from loss of light exposure during dark-rearing. We did not intend to suggest that visual experience acting via local neuronal activity modulates astrocyte GR binding.

To avoid ambiguity, we have replaced "visual experience" with "light" in the Results section, where we discuss the effects of light deprivation on circulating CORT and GR binding in astrocytes. In addition, we have also clarified that the effect of light on CORT

levels could likely be driven via the SCN subcortical pathway on page 3 of our revised manuscript.

4) The validation of the viral approach to delete GR from astrocytes is impressive, it is very rare to observe a 100% KD efficiency. In the representative extended data figure Fig 7C, it is striking that there does not appear to be any GR expression outside of the nuclear area. As GR translocates from the cytosol to the nucleus upon binding to its ligand, these representative images suggest that the entirety of GR is ligand bound (only found in nuclear compartment) which would be indicative of an important stress response (very high CORT levels). Can the authors comment further on this observation?

All mice were harvested at ZT12-14, the peak of circadian CORT levels, to maximize nuclear localization of GR for improved detection by immunofluorescent staining. The high efficiency of Cre-Lox-mediated genetic deletion in cells expressing Cre recombinase is consistent with our observations in other floxed lines in our lab (Bloodgood et al. 2013 Nature; Cheadle et al. 2020 Neuron; Yap et al. 2021 Nature; Pollina et al. 2023 Nature). To address the reviewer's concern, this point is now clarified in the Methods of our revised manuscript on page 10.

5) Additionally, low magnification images to demonstrate specific targeting of visual cortex and viral spread would be important additions to the manuscript for future reproducibility.

As requested by the reviewer, we have now provided low-magnification images of GFP immunofluorescent staining in V1 of P35 Cre- or ΔCre-injected animals in Extended Data Figure 8g.

6) The authors should determine whether all astrocytes express GR or whether it exists only in a subset of cells. Other work suggests enrichment of GR in specific populations of astrocytes (Chung et al. Nature 2025). Indeed, other stress-related receptors e.g. oxytocin receptors are only expressed in a subset of cells (Wahis et al. Nat Neurosci 2021), and astrocytes are highly diverse within cortical structures (Bayraktar et al. Nat Neurosci 2020). The authors already have the single-cell data available so this would be highly interesting and valuable insight to add. If the authors do observe this diversity within their region of interest, does this contribute to the higher variability in astrocyte morphology in controls (GR+ and potentially GR-) compared to GR-KO mice (which they show results in a 100% KO efficiency)?

Although we are hesitant to draw strong conclusions on GR mRNA expression from snRNA-seq data, as these methods often cannot distinguish between technical dropout

and true lack of gene expression, we have re-analyzed our IF staining data to quantify the proportion of GR+/Sox9+ cells in ΔCre-T2a-GFP-injected control mice (Extended Data Fig. 8l-m). We find that essentially all Sox9+ nuclei co-express GR protein, indicating minimal heterogeneity in GR expression across V1 astrocytes. We have included a brief mention of this finding in the revised text on page 5.

7) In figure 4C-D, the authors set out to dissect the relationship between circadian fluctuations in glucocorticoids and astrocytic regulation of PNNs. To do this they used their GR KO approach, which is a permanent manipulation, but it's not clear whether this specific experiment addresses their question. To determine whether "daily astrocyte GR activation might be required to sustain PNNs in the mature brain," the authors should manipulate CORT levels. Following this initial characterisation of daily fluctuations on CORT on PNNs, the deletion of GR in this instance would add causality.

We thank the reviewer for raising this issue. We have amended the text, differentiating between "daily astrocyte GR activation" and "astrocyte GR signaling" to better reflect the nature of this experiment, as we did not directly seek to examine whether circadian CORT regulates PNNs.

In addition, in response to a point raised by reviewer #1, we have examined whether pharmacological CORT administration to dark-reared (DR) animals around eye-opening (P14) alters the kinetics of PNN formation. We found that raising the level of CORT promotes PNN assembly, as previously suggested by our astrocyte GR-KO experiments in neonatal and adult animals (Fig. 4a-d).

We have included these results in Extended Data Fig. 16 and added the corresponding text to the Results section on page 7 of our revised manuscript.

8) Is it surprising that PNN maturity had no effect on IPSCs or EPSCs onto Pyramidal neurons? From my understanding PNNs are highly implicated in synaptic plasticity. As such, a synaptic plasticity manipulation, rather than investigating basal transmission, might be more appropriate.

We agree that it is not surprising that changes in PNN levels do not affect mIPSCs or mEPSCs. However, because we lacked a priori expectations about the role of astrocyte GR signaling in neuronal development, we initially measured these parameters based on the well-established role of astrocytes in synapse formation.

As the reviewer points out, PNNs are certainly more linked to synaptic plasticity than basal synaptic transmission. To examine the effect of GR KO on synaptic plasticity, we

invested considerable time and effort together with the laboratory of Dr. Chinfei Chen to test the requirement of astrocytic GR for the closure of the ocular dominance critical period in mice. We delivered astrocyte-selective AAVs expressing Cre or catalytically-inactive ΔCre into V1 in adult (P35-P40) GR$^{fl/fl}$ mice and, after waiting three weeks for viral transduction, recorded visual responses from binocular V1 using multi-electrode arrays following normal rearing (NR) or 3-4 days of monocular deprivation (MD). We found that eliminating GR in V1 astrocytes restores ODP in adult mice, as indicated by an increase in the relative strength of visual responses from the non-deprived eye following MD without affecting responses in NR animals.

We have added these findings to the final section of the Results on page 8 and included the data in Figure 5 and Extended Data Figure 19.

9) Regarding these electrophysiological manipulations, are the neurons recorded adjacent to, or within the domain of GR KO astrocytes or not?

Neurons were recorded within the domain of astrocyte GFP expression (used as a marker of viral transduction), as visualized on our electrophysiology rig epifluorescent microscope. This point is now described in the revised manuscript on page 7.

10) In the introduction, the authors indicate that, “the specific mechanisms by which glia promote critical period closure have not been identified.” This is not entirely accurate, as an earlier study (cited by the authors) went into considerable detail showing a potential role for astrocyte connexins and regulation of the extracellular matrix through MMP-9 (Ribot et al, Science, 2021). How do the current findings fit with these previous observations, given that the GCR and connexins have been linked in the stress literature.

We thank the reviewer for raising this point. We have resolved this by removing this sentence and combining the two preceding sentences on page 1.

Our data are consistent with Ribot et al. 2021 Science – Gjb6/Cx30 is one of the top astrocyte GR-activated genes in our datasets with a strong, conserved (across humans and mice) GR binding site in the first intron (shown in Extended Data Fig. 12c and Extended Data Fig. 14m-n). We now include this point on page 5 of our revised manuscript.

Minor

1) Please verify the statistics on Figure 2C (P10 timepoint) as it does not appear to be significant.

The statistics for the P10 CORT comparison in Figure 2C are significant ($p < 0.05$ with multiple hypothesis correction), though the CORT levels at this time (~7.6 ng/mL for NR and ~3.7 ng/mL for DR at P10) are an order of magnitude lower than the later time points.

2) In the final sentence of the results the authors state that “As dark-rearing has been shown to delay the physiological, transcriptional, and structural maturation of V1, we conclude that eliminating astrocyte GR function likewise shifts V1 neurons to an immature state.” How can the authors definitely state that this remains immature and is not simply delayed as they previous literature they cite has demonstrated?

We thank the reviewer for raising this point. We have removed this statement on page 8 to avoid over-interpreting the snRNA-seq data, given that we did not profile additional time points or comprehensively measure other aspects of neuronal maturity.

3) The authors state that “these data indicate light exposure around eye-opening induces systemic CORT production”. Based on their data in figure 2C, this appears to be an overstatement. While light-exposure enhanced systemic CORT production around eye-opening, CORT was still elevated at this time point, compared to P10, and both conditions had identical blood CORT levels by P21.

We agree that “induces” is over-stating. We have replaced this word with “enhances” to more accurately reflect the data.

4) The authors discuss the relevance of targeting GR in astrocytes in early life stress. A recent publication on this subject has been published showing how deletion of GR in astrocytes rescues the effects of early life stress (Depaauw-Holt et al. Nat Comms 2025) and should be discussed in the context of this work.

We thank the reviewer for bringing this publication to our attention, and we agree with its relevance to the final paragraph of the Discussion. We now mention this work on page 9 of the revised manuscript, while acknowledging the request of reviewers #1 and #3 to tone down this section.

5) Can the authors comment on the trends observed in Figure 1C. Why do experience-dependent genes decrease in glial cell types following P28, while neuronal cells continue to increase. Is this not surprising? Shouldn’t all cell types scale similarly as glial activity has been shown to be critical for neuronal plasticity across development and in adult mice? One interpretation of these data is that over time the difference between DD and LD rearing narrows suggesting a slowed development that might reach equivalence with time.

We thank the reviewer for raising this point. While we had not previously examined the surge in glial responses at P28, we agree that it is notable. To address the reviewer's point, we have now analyzed the genes altered by visual experience in glial cell types – oligodendrocytes, microglia, and astrocytes – at P28 and found strong enrichment for genes involved in cell type-specific maturational processes occurring at this stage in development.

In oligodendrocytes, visual experience at P28 induces a broad gene program that promotes maturation and myelination, including Olig1, Olig2, Sox10, Myrf, Mbp, Plp1, Mog, Mobp, Cnp, Ugt8a, Fa2h, Gal3st1, Mal, Ermn, and Cldn11, among others. Notably, this timing coincides with the onset of oligodendrocyte-axon contact and myelination in V1 (McGee et al. 2005 Science) and aligns with the well-established role of neuronal activity in promoting myelination (Bonetto et al. 2021 Science).

Likewise, in microglia, visual experience induces genes characteristic of the transition from a developmental phagocytic state to a mature surveillance state, including Tgfb1, Tgfbr1 and downstream TGF-b signaling targets such as Tmem119, Hexb, Fcrls, P2ry12, Golm1, Mertk, among others (Butovsky et al. 2013 Nat Neurosci; Spittau et al. 2020 Trends Immunol), as well as Csf1r (Kierdorf et al. 2013 Nat Neurosci). Consistent with this observation, dark-rearing is known to occlude the transition of V1 microglia from a phagocytic state to a mature state (Tremblay et al. 2010 PLoS Biol). We also observed experience-induced expression of metabolic genes in microglia, including P2ry12 and Entpd1/CD39, which mediate negative feedback control of neuronal activity (Badimon et al. 2020 Nature).

The precise reason why glial responses to visual deprivation surge at P28 and decline by P35 remains unclear. We speculate that the reduction in experience-dependent responses at P35 may reflect either a transient induction of these gene programs following initial activation of the circuit at P21 or a delayed response under dark-rearing, which has previously been shown to postpone other aspects of V1 development (Fagiolini et al. 1994 Vision Res; Tropea et al. 2010 J Neurosci).

To address the reviewer's point, we have now included a brief description of these findings in the Results on page 2 of the revised manuscript.

6) It doesn't appear that sex as a biological variable has been addressed. Although the authors used both sexes, considering recent data (Depaauw-Holt et al. Nat Comms 2025) showing dramatic sex differences in astrocytic responses to stress, there could be interesting findings in their datasets. This is not essential but appears to be a missed opportunity.

We agree that sex is an important biological variable that was not comprehensively addressed in our experiments. One prior study (Liu et al. 2020 Neurobiol Stress) examined ocular dominance plasticity in female and male mice across development (P21-P22, P28-P29, P37-P38) and in adulthood (>P60) and found no sex differences at any time point (Fig. 3). These findings suggest that, if baseline astrocyte GR signaling differs between sexes, such differences do not appear to influence the overall timing of critical period closure.

For our CORT ELISA and LC-MS data as well as our SHARE-seq dataset, we believe our experiments were sufficiently powered to examine potential sex differences. We now include additional panels in Extended Data Fig. 6 (ELISA and LC-MS) and Extended Data Fig. 9 (SHARE-seq astrocyte visual experience-dependent gene program), which show comparable CORT levels and astrocyte experience-dependent gene expression across sexes, respectively. We also examined whether the astrocyte GR-dependent gene program, which we previously observed to be conserved across mouse brain regions (Fig. 3e) and in the developing human brain (Fig. 3h-k), differs by sex and found similar results between females and males. These analyses are now incorporated into Extended Data Figs. 12 and 14, respectively. In the future, it will be important to more deeply characterize GR-dependent gene regulation in the brain between sexes across the circadian cycle and following early-life stress.

Referee #1 (Remarks to the Author):

Summary:

In the revised manuscript, the addition of monocular deprivation experiments demonstrating reopening of ocular dominance plasticity following astrocyte-specific GR deletion in adults represents an important functional advance. This experiment directly addresses a central concern in the initial review and significantly improves the manuscript's mechanistic depth. The additional analyses of PV interneuron density, PNN prevalence, paired-pulse measurements, and earlier validation of GR-cKO further enhance the study's rigor and completeness.
Overall, this remains an exceptionally rich and technically sophisticated dataset of broad interest to developmental neurobiology and glial biology. The integration of epigenomic profiling with circuit-level manipulation is a major strength.
At the same time, several conceptual and interpretational issues remain that should be addressed before publication. These largely concern the strength of causal claims and the scope of certain conclusions. The additional data support a model in which astrocyte GR signaling contributes to ECM maturation and limits plasticity; however, some statements continue to extend beyond what has been directly demonstrated.

We thank the reviewer for recognizing the improvements in our study and for continuing to provide valuable feedback.

The manuscript would benefit from further refining the language to ensure that the conclusions align precisely with the experimental evidence.

Remaining Concerns:

1- Causality of corticosterone–GR signaling: The manuscript continues to frame the light-induced corticosterone surge as a driver of GR activation in astrocytes and downstream maturation programs. While the dark-rearing plus CORT supplementation experiments support a role for glucocorticoids in ECM maturation, they do not directly demonstrate that endogenous corticosterone is necessary for astrocyte GR chromatin recruitment, nor that CORT is sufficient to induce GR genomic binding in vivo.
Thus, the causal step linking light to systemic CORT and, in turn, to astrocyte GR chromatin recruitment remains inferred rather than demonstrated.

I recommend revising the language throughout to clarify that:
- Light enhances systemic corticosterone production, which is consistent with GR activation in astrocytes.
- The data support, but do not establish, the necessity and sufficiency of endogenous CORT for astrocyte GR genomic recruitment.
Framing this step as a supported model rather than a demonstrated causal chain would more accurately reflect the evidence presented. Otherwise, additional experiments are needed to support this claim.

We thank the reviewer for raising this important point regarding the mechanism of GR activation in astrocytes and for their useful suggestions for how to improve the text. In our revised manuscript, we have been careful not to claim that light-induced CORT drives GR recruitment in astrocytes. Rather, we say that light enhances systemic CORT levels and that light also promotes GR recruitment to chromatin. We have modified two sentences on pages 6 and 9 of the manuscript that inferred a role for endogenous CORT in astrocyte maturation or developmental plasticity, replacing "CORT" or "glucocorticoid" with "GR signaling".

2- Critical period closure versus restriction of plasticity: The newly added adult monocular deprivation experiments convincingly demonstrate that astrocyte GR deletion reopens ocular dominance plasticity in adulthood. This is an important finding. However, the manuscript continues to state, in several places (including the Abstract), that astrocyte GR signaling "drives critical period closure." The developmental closure of the critical period itself was not directly tested (e.g., through developmental MD paradigms across the CP window). Instead, the data show that GR signaling promotes ECM/PNN maturation and constrains plasticity, and that its removal permits plasticity in adults.

I suggest modifying the language from:
"promotes critical period closure."
to
"promotes ECM maturation and limits plasticity," or
"contributes to the restriction of plasticity associated with critical period closure."
This distinction is important and will enhance the manuscript's precision.

We thank the reviewer for drawing our attention to this point. We have not examined whether astrocyte GR signaling restrains ocular dominance plasticity during postnatal development. In line with the reviewer's suggestions, we have modified the text in the Abstract (page 1) and Main (page 2) sections so that it no longer states that astrocyte GR signaling closes the V1 critical period.

3. Human data and evolutionary conservation
Human analyses show compelling cross-species concordance in motif accessibility and gene program overlap. However, the data remain correlative. There is no functional manipulation in human tissue (which I agree is not trivial), demonstrating that GR drives astrocyte maturation programs in an analogous manner.
Statements referring to an "evolutionarily conserved GR-driven maturation program" should therefore be tempered to reflect concordance or conservation at the level of chromatin accessibility and gene overlap, rather than functional conservation of mechanism.

We agree with this point and have replaced the phrase in the Abstract that says "evolutionarily conserved GR-driven maturation program" with the words "an extensive gene regulatory program that is partially conserved during human brain development".

4. Interpretation of neuronal transcriptional effects: The snRNA-seq dataset (n = 2 animals per condition) provides suggestive evidence that astrocyte GR-cKO induces neuronal transcriptional signatures reminiscent of those observed in dark-reared animals. However, given the limited sample size and absence of functional neuronal measurements at the same developmental time point, claims regarding astrocyte-dependent suppression of neuronal maturation programs should be framed cautiously.
A brief acknowledgment of these limitations in the Results or Discussion is needed.

We thank the reviewer for raising this point. We now acknowledge in the Results on page 9 that additional functional measurements are required to conclusively show that the astrocyte GR-dependent effects on neuronal gene expression impact neuronal maturation.

5. Broader implications for stress and psychiatric disease: The Discussion and Abstract continue to extend the findings toward early-life stress and psychiatric vulnerability. While these are intriguing and plausible hypotheses, they are not experimentally tested here.
Maintaining explicit hypothesis language ("may," "could," "raises the possibility") and avoiding definitive phrasing in the Abstract would improve conceptual balance.

We have modified the final sentence of the Abstract to say that our study's relevance to disease is speculative ("may contribute", "may increase susceptibility"). We feel that the final paragraph of the Discussion acknowledges that early-life stress is an area of future investigation and not examined in the current work. Throughout this section, we have been careful to phrase these ideas as hypotheses ("raises the possibility", "may provide an entry point", "it will be important to examine").

Minor Clarifications
- Where GR genomic recruitment is described as astrocyte-selective, it would be helpful to briefly acknowledge potential technical differences in cell-type isolation or detection sensitivity that may influence peak counts.
- Ensure consistent use of causal language (e.g., "supports a model in which" rather than "reveals" or "demonstrates") when referring to multi-step mechanistic inferences.

We have included a statement at the end of page 4 acknowledging that GR binding may still occur in sparse cell subtypes that were not detected in our analyses. We have also reviewed the text and have eliminated causal language and have softened our conclusions by using the words "indicates", "suggests", "supports a model in which" where appropriate.

Referee #3 (Remarks to the Author):

The authors have addressed all my original concerns.

We thank the reviewer for their constructive feedback on our manuscript.

Referee #4 (Remarks to the Author):

The revised manuscript by Gegenhuber and colleagues has been significantly strengthened following input from all three reviewers. The authors have performed a commendable amount of experimental work to address all core issues raised by the reviewers and made important text edits to improve clarity of the manuscript.

In the current form, this manuscript represents an important addition to the field, meeting the high standards expected from an article published in Nature. I wish to congratulate the authors on this work and look forward to seeing the manuscript in print.

We are grateful that this reviewer recognizes the strengths in our study, and we thank the reviewer for their helpful suggestions.
